# Supplementary material for: TNFR1 Suppression by XPro1595 Reduces Peripheral Neuropathies Associated with Perineural Invasion in Female Mice
Source: Cells. 2025 Nov 7;14(22):1749. doi: 10.3390/cells14221749 (PMC12651030; doi:10.3390/cells14221749)
Supplement: Supplementary file 1 [file cells-14-01749-s001.zip › cells-3924103-supplementary.pdf]

Upregulated DEGs

Rank	Gene	Manhattan distance	baseMean	log2FoldChange	FC	lfcSE	padj	minus_log10_padj	Gene Name found in the DAVID Knowledgebase	ENTREZ Gene ID NO.
		|ΔX| +|ΔY|		ΔX				ΔY		
1	**Serping1**	135.22	2693	2.814	7.03	0.1133	3.93E-133	132.405	serine (or cysteine) peptidase inhibitor, clade G, member 1 (Serping1)	12258
2	**Hp**	127.92	1403	3.432	10.79	0.1429	3.22E-125	124.492	Haptoglobin (Hp)	15439
3	**Apod**	92.43	3678	3.387	10.46	0.1669	9.02E-90	89.045	apolipoprotein D (Apod)	11815
4	**Mmp3**	77.68	557	3.559	11.79	0.1917	7.52E-75	74.124	matrix metallopeptidase 3 (Mmp3)	17392
5	**C4b**	64.40	1499	2.419	5.35	0.1421	1.04E-62	61.985	complement C4B (Chido blood group) (C4b)	12268
6	**C3**	56.54	4340	2.482	5.59	0.1562	8.74E-55	54.058	complement component 3 (C3)	12266
7	**Pmp22**	48.09	984	2.417	5.34	0.1649	2.13E-46	45.672	peripheral myelin protein 22 (Pmp22)	18858
8	**Cilp**	43.05	745	3.044	8.25	0.2228	9.90E-41	40.004	cartilage intermediate layer protein, nucleotide pyrophosphohydrolase(Cilp)	214425
9	**Mpz**	41.97	389	4.152	17.78	0.3155	1.52E-38	37.819	myelin protein zero (Mpz)	17528
10	**Gapdh**	40.65	10928	1.366	2.58	0.1004	5.17E-40	39.287	glyceraldehyde-3-phosphate dehydrogenase (Gapdh)	14433
11	**Serpina3n**	37.80	1255	2.875	7.34	0.2255	1.19E-35	34.925	serine (or cysteine) peptidase inhibitor, clade A, member 3N (Serpina3n)	20716
12	**Rcn3**	37.41	836	1.823	3.54	0.1408	2.62E-36	35.582	reticulocalbin 3, EF-hand calcium binding domain (Rcn3)	52377
13	**Lox**	37.02	4231	1.442	2.72	0.1115	2.62E-36	35.582	lysyl oxidase (Lox)	16948
14	**Trim63**	36.57	360	4.558	23.55	0.3725	9.81E-33	32.008	tripartite motif-containing 63 (Trim63)	433766
15	**Mfap4**	36.24	427	2.403	5.29	0.1906	1.45E-34	33.839	microfibrillar-associated protein 4 (Mfap4)	76293
16	**C1qtnf3**	35.85	1389	2.249	4.75	0.1793	2.50E-34	33.602	C1q and tumor necrosis factor related protein 3 (C1qtnf3)	81799
17	**Cox7b**	32.46	935	1.684	3.21	0.1400	1.68E-31	30.774	cytochrome c oxidase subunit 7B (Cox7b)	66142
18	**Fabp4**	31.96	677	2.958	7.77	0.2553	9.94E-30	29.003	fatty acid binding protein 4, adipocyte (Fabp4)	11770
19	**Igfn1**	31.92	409	4.225	18.71	0.3666	2.02E-28	27.695	immunoglobulin-like and fibronectin type III domain containing 1 (Igfn1)	226438
20	**Serpine1**	31.91	1783	1.679	3.20	0.1409	5.90E-31	30.229	serine (or cysteine) peptidase inhibitor, clade E, member 1 (Serpine1)	18787

21	**Gpc3**	31.34	340	2.752	6.74	0.2384	2.56E-29	28.592	glypican 3 (Gpc3)	14734
22	**Dcn**	30.25	6390	2.109	4.31	0.1842	7.19E-29	28.143	Decorin (Dcn)	13179
23	**Eef1a2**	30.10	304	5.257	38.24	0.4905	1.42E-25	24.847	eukaryotic translation elongation factor 1 alpha 2 (Eef1a2)	13628
24	**Mfap5**	29.86	719	1.858	3.63	0.1625	9.85E-29	28.007	microfibrillar associated protein 5 (Mfap5)	50530
25	**Rpl26**	29.81	2781	1.392	2.63	0.1204	3.87E-29	28.413	ribosomal protein L26 (Rpl26)	19941
26	**C1s1**	29.35	1373	2.086	4.25	0.1845	5.41E-28	27.267	complement component 1, s subcomponent 1 (C1s1)	50908
27	**Serpinh1**	29.27	5662	1.146	2.21	0.0997	7.44E-29	28.128	serine (or cysteine) peptidase inhibitor, clade H, member 1 (Serpinh1)	12406
28	**Mypn**	29.01	257	5.320	39.93	0.5037	2.03E-24	23.692	Myopalladin (Mypn)	68802
29	**Tcap**	27.24	381	5.121	34.80	0.4775	7.60E-23	22.119	titin-cap (Tcap)	21393
30	**Dpt**	27.19	881	2.294	4.90	0.2139	1.27E-25	24.895	Dermatopontin (Dpt)	56429
31	**Nme2**	25.84	2293	1.226	2.34	0.1140	2.41E-25	24.618	NME/NM23 nucleoside diphosphate kinase 2 (Nme2)	18103
32	**Scn7a**	25.75	836	2.520	5.74	0.2434	5.95E-24	23.226	sodium channel, voltage-gated, type VII, alpha (Scn7a)	20272
33	**B2m**	25.68	8298	1.187	2.28	0.1107	3.23E-25	24.491	beta-2 microglobulin (B2m)	12010
34	**C1ra**	25.51	485	2.033	4.09	0.1946	3.30E-24	23.481	complement component 1, r subcomponent A (C1ra)	50909
35	**Col14a1**	25.14	2267	2.167	4.49	0.2106	1.07E-23	22.971	collagen, type XIV, alpha 1 (Col14a1)	12818
36	**F13a1**	25.10	1491	1.878	3.67	0.1811	6.01E-24	23.221	coagulation factor XIII, A1 subunit (F13a1)	74145
37	**Mgp**	25.03	2004	1.230	2.35	0.1162	1.58E-24	23.800	matrix Gla protein (Mgp)	17313
38	**Postn**	24.98	20711	1.525	2.88	0.1455	3.52E-24	23.454	periostin, osteoblast specific factor (Postn)	50706
39	**Rpl29**	24.72	2243	1.159	2.23	0.1101	2.72E-24	23.566	ribosomal protein L29(Rpl29)	19944
40	**Lpl**	24.43	880	2.086	4.25	0.2054	4.53E-23	22.344	lipoprotein lipase (Lpl)	16956
41	**Ubb-ps**	24.40	2789	1.174	2.26	0.1126	6.01E-24	23.221	ubiquitin B, pseudogene (Ubb-ps)	218963
42	**Myo18b**	24.33	408	4.410	21.26	0.4495	1.19E-20	19.923	myosin XVIIIb (Myo18b)	74376
43	**Ubb**	24.06	8381	1.244	2.37	0.1205	1.54E-23	22.813	ubiquitin B (Ubb)	22187
44	**Laptm4a**	23.56	4446	0.988	1.98	0.0958	2.65E-23	22.577	lysosomal-associated protein transmembrane 4A (Laptm4a)	17775
45	**Ccdc80**	23.53	1592	1.701	3.25	0.1693	1.50E-22	21.824	coiled-coil domain containing 80 (Ccdc80)	67896
46	**Scd1**	23.45	4350	2.001	4.00	0.2005	3.52E-22	21.453	stearoyl-Coenzyme A desaturase 1 (Scd1)	20249
47	**Pdlim3**	23.45	492	3.615	12.25	0.3763	1.47E-20	19.834	PDZ and LIM domain 3 (Pdlim3)	53318
48	**Cfb**	23.44	1186	2.145	4.42	0.2170	5.07E-22	21.295	complement factor B (Cfb)	14962

49	**Rps7**	23.41	5067	0.976	1.97	0.0950	3.68E-23	22.434	ribosomal protein S7 (Rps7)	20115
50	**Rpl31**	23.14	2975	1.088	2.13	0.1071	8.97E-23	22.047	ribosomal protein L31 (Rpl31)	114641
51	**Aspn**	23.13	704	1.916	3.77	0.1930	6.06E-22	21.217	asporin (Aspn)	66695
52	**Rpl10**	22.71	4876	0.957	1.94	0.0946	1.75E-22	21.757	ribosomal protein L10 (Rpl10)	110954
53	**Rps26**	22.56	5850	1.070	2.10	0.1066	3.25E-22	21.488	ribosomal protein S26 (Rps26)	27370
54	**Mmrn1**	22.54	301	3.027	8.15	0.3196	3.04E-20	19.517	multimerin 1 (Mmrn1)	70945
55	**Rny3**	22.47	797	2.202	4.60	0.2278	5.41E-21	20.267	RNA, Y3 small cytoplasmic (associated with Ro protein) (Rny3)	19874
56	**Cstb**	22.44	1029	1.302	2.47	0.1312	7.30E-22	21.137	cystatin B (Cstb)	13014
57	**Cfh**	22.36	3032	1.262	2.40	0.1272	8.02E-22	21.096	complement component factor h (Cfh)	12628
58	**Lbp**	22.27	869	2.309	4.96	0.2420	1.09E-20	19.963	lipopolysaccharide binding protein (Lbp)	16803
59	**Adgrd1**	22.07	398	2.206	4.61	0.2309	1.36E-20	19.867	adhesion G protein-coupled receptor D1 (Adgrd1)	243277
60	**Sfrp4**	21.98	614	2.253	4.77	0.2372	1.87E-20	19.729	secreted frizzled-related protein 4 (Sfrp4)	20379
61	**Rpl27**	21.86	2019	1.106	2.15	0.1126	1.77E-21	20.752	ribosomal protein L27 (Rpl27)	19942
62	**Ccl8**	21.56	578	2.565	5.92	0.2761	1.01E-19	18.995	C-C motif chemokine ligand 8 (Ccl8)	20307
63	**Oat**	21.07	1500	1.162	2.24	0.1205	1.25E-20	19.904	ornithine aminotransferase (Oat)	18242
64	**Islr**	20.72	606	1.479	2.79	0.1567	5.72E-20	19.242	immunoglobulin superfamily containing leucine-rich repeat (Islr)	26968
65	**Rpl32**	20.70	2993	0.966	1.95	0.1005	1.86E-20	19.732	ribosomal protein L32 (Rpl32)	19951
66	**Cxcl14**	20.64	2713	1.351	2.55	0.1431	5.12E-20	19.291	C-X-C motif chemokine ligand 14 (Cxcl14)	57266
67	**C1qb**	20.28	2704	0.983	1.98	0.1034	5.09E-20	19.293	complement component 1, q subcomponent, beta polypeptide (C1qb)	12260
68	**Pdpn**	19.93	1298	1.598	3.03	0.1734	4.65E-19	18.333	Podoplanin (Pdpn)	14726
69	**Rps27l**	19.84	799	1.472	2.77	0.1594	4.24E-19	18.373	ribosomal protein S27-like (Rps27l)	67941
70	**Sulf1**	19.46	2215	1.444	2.72	0.1579	9.62E-19	18.017	sulfatase 1 (Sulf1)	240725
71	**Efemp1**	19.43	452	2.127	4.37	0.2386	4.99E-18	17.302	epidermal growth factor-containing fibulin-like extracellular matrix protein 1 (Efemp1)	216616
72	**Ppib**	19.40	2750	0.981	1.97	0.1057	3.84E-19	18.415	peptidylprolyl isomerase B (Ppib)	19035
73	**Cd36**	19.22	488	2.391	5.25	0.2723	1.49E-17	16.828	CD36 molecule (Cd36)	12491
74	**Rmrp**	19.16	13062	1.256	2.39	0.1378	1.26E-18	17.901	RNA component of mitochondrial RNAase P (Rmrp)	19782

75	**Rnase4**	19.14	1850	1.227	2.34	0.1344	1.22E-18	17.915	ribonuclease, RNase A family 4 (Rnase4)	58809
76	**Lgals1**	19.02	10797	0.892	1.86	0.0965	7.48E-19	18.126	lectin, galactose binding, soluble 1 (Lgals1)	16852
77	**Acta2**	19.01	1979	1.051	2.07	0.1148	1.10E-18	17.957	actin alpha 2, smooth muscle, aorta (Acta2)	11475
78	**Slc25a5**	18.65	2603	1.028	2.04	0.1135	2.36E-18	17.626	solute carrier family 25 (mitochondrial carrier, adenine nucleotide translocator), member 5 (Slc25a5)	11740
79	**Myot**	18.65	464	5.955	62.03	0.7870	2.03E-13	12.692	Myotilin (Myot)	58916
80	**Steap4**	17.82	533	2.371	5.17	0.2814	3.58E-16	15.446	STEAP family member 4 (Steap4)	117167
81	**Rps4x**	17.79	5369	0.832	1.78	0.0932	1.11E-17	16.956	ribosomal protein S4, X-linked (Rps4x)	20102
82	**Gm10275**	17.66	817	1.241	2.36	0.1422	3.81E-17	16.419	predicted pseudogene 10275 (Gm10275)	19897
83	**Mgst1**	17.65	643	1.406	2.65	0.1621	5.66E-17	16.247	microsomal glutathione S-transferase 1 (Mgst1)	56615
84	**Actn3**	17.63	2613	4.987	31.72	0.6619	2.26E-13	12.646	actinin alpha 3 (Actn3)	11474
85	**Rpl12**	17.29	7159	0.911	1.88	0.1040	4.20E-17	16.376	ribosomal protein L12(Rpl12)	269261
86	**C4a**	17.27	249	2.153	4.45	0.2593	7.72E-16	15.113	complement C4A (Rodgers blood group) (C4a)	625018
87	**Gm23547**	17.24	216	2.036	4.10	0.2440	6.32E-16	15.199	predicted gene, 23547 (Gm23547)	115490496
88	**Rps29**	17.09	2385	1.060	2.08	0.1228	9.28E-17	16.033	ribosomal protein S29 (Rps29)	20090
89	**Srpx**	17.04	246	1.949	3.86	0.2342	8.05E-16	15.094	sushi-repeat-containing protein (Srpx)	51795
90	**Ms4a6d**	16.86	787	1.249	2.38	0.1470	2.46E-16	15.608	membrane-spanning 4-domains, subfamily A, member 6D (Ms4a6d)	68774
91	**Myl1**	16.81	1664	6.279	77.67	0.8722	2.97E-11	10.528	myosin, light polypeptide 1 (Myl1)	17901
92	**Rpl39**	16.78	2757	1.140	2.20	0.1337	2.28E-16	15.642	ribosomal protein L39 (Rpl39)	67248
93	**Mrc1**	16.74	5143	1.081	2.12	0.1268	2.20E-16	15.657	mannose receptor, C type 1 (Mrc1)	17533
94	**Art1**	16.65	141	4.938	30.66	0.6769	1.95E-12	11.710	ADP-ribosyltransferase 1 (Art1)	11870
95	**Rpl6**	16.61	6848	0.836	1.78	0.0971	1.70E-16	15.771	ribosomal protein L6 (Rpl6)	19988
96	**Lum**	16.59	1630	1.791	3.46	0.2172	1.59E-15	14.798	Lumican (Lum)	17022
97	**Tmem182**	16.51	131	4.540	23.26	0.6103	1.07E-12	11.970	transmembrane protein 182 (Tmem182)	381339
98	**Ccl21a**	16.48	245	2.719	6.58	0.3458	1.74E-14	13.759	C-C motif chemokine ligand 21 (serine) (Ccl21a)	18829
99	**Ccdc107**	16.44	699	2.033	4.09	0.2508	3.92E-15	14.406	coiled-coil domain containing 107 (Ccdc107)	622404
100	**Rpl24**	16.37	2238	1.115	2.17	0.1326	5.59E-16	15.253	ribosomal protein L24 (Rpl24)	68193
101	**Fabp5**	16.33	728	1.229	2.34	0.1469	7.86E-16	15.104	fatty acid binding protein 5, epidermal (Fabp5)	16592

102	**Gm2606**	16.11	720	1.269	2.41	0.1532	1.44E-15	14.842	predicted pseudogene 2606 (Gm2606)	100040109
103	**Fcgrt**	16.07	679	1.583	3.00	0.1947	3.25E-15	14.488	Fc fragment of IgG receptor and transporter (Fcgrt)	14132
104	**Rny1**	16.04	873	1.875	3.67	0.2339	6.87E-15	14.163	RNA, Y1 small cytoplasmic, Ro-associated (Rny1)	19872
105	**Cd209f**	16.00	104	2.990	7.95	0.3896	9.88E-14	13.005	CD209f antigen (Cd209f)	69142
106	**Svep1**	15.85	2539	1.546	2.92	0.1905	4.98E-15	14.303	sushi, von Willebrand factor type A, EGF and pentraxin domain containing 1 (Svep1)	64817
107	**Rpl36**	15.81	1023	1.647	3.13	0.2054	6.89E-15	14.162	ribosomal protein L36 (Rpl36)	54217
108	**Tubb6**	15.78	1890	1.054	2.08	0.1273	1.89E-15	14.722	tubulin, beta 6 class V (Tubb6)	67951
109	**Srpx2**	15.65	461	1.839	3.58	0.2311	1.55E-14	13.809	sushi-repeat-containing protein, X-linked 2 (Srpx2)	68792
110	**Cd68**	15.45	916	1.249	2.38	0.1543	6.22E-15	14.206	CD68 antigen (Cd68)	12514
111	**Bgn**	15.45	15894	1.186	2.28	0.1460	5.50E-15	14.260	Biglycan (Bgn)	12111
112	**Ckm**	15.39	3950	5.068	33.55	0.7510	4.81E-11	10.318	creatine kinase, muscle (Ckm)	12715
113	**Thy1**	15.28	2324	0.863	1.82	0.1051	3.86E-15	14.413	thymus cell antigen 1, theta (Thy1)	21838
114	**Atp5o**	15.24	1223	1.032	2.04	0.1270	6.14E-15	14.212	No gene name found in the DAVID Knowledgebase	0
115	**Rps18**	15.16	3037	0.904	1.87	0.1108	5.53E-15	14.257	ribosomal protein S18 (Rps18)	20084
116	**Serpinf1**	15.13	3071	1.406	2.65	0.1772	1.88E-14	13.726	serine (or cysteine) peptidase inhibitor, clade F, member 1 (Serpinf1)	20317
117	**Grb10**	15.07	1649	0.927	1.90	0.1143	7.23E-15	14.141	growth factor receptor bound protein 10 (Grb10)	14783
118	**Tpi1**	14.99	4607	0.770	1.71	0.0943	6.06E-15	14.218	triosephosphate isomerase 1 (Tpi1)	21991
119	**Smpdl3a**	14.93	324	1.638	3.11	0.2109	5.14E-14	13.289	sphingomyelin phosphodiesterase, acid-like 3A (Smpdl3a)	57319
120	**Lgmn**	14.92	4342	0.843	1.79	0.1038	8.43E-15	14.074	Legumain (Lgmn)	19141
121	**Tyrobp**	14.80	845	1.243	2.37	0.1577	2.79E-14	13.554	TYRO protein tyrosine kinase binding protein (Tyrobp)	22177
122	**Vcan**	14.72	15533	1.084	2.12	0.1363	2.30E-14	13.638	Versican (Vcan)	13003
123	**C2**	14.71	167	2.160	4.47	0.2862	2.82E-13	12.550	complement C2 (C2)	12263
124	**Rcn1**	14.69	2030	0.957	1.94	0.1199	1.85E-14	13.732	reticulocalbin 1 (Rcn1)	19672
125	**Ogn**	14.65	963	1.351	2.55	0.1736	5.06E-14	13.296	Osteoglycin (Ogn)	18295
126	**Tagln**	14.64	858	1.234	2.35	0.1572	3.97E-14	13.401	Transgelin (Tagln)	21345
127	**Gas6**	14.62	1511	1.453	2.74	0.1880	6.80E-14	13.167	growth arrest specific 6 (Gas6)	14456
128	**Gstm2**	14.62	281	1.895	3.72	0.2505	1.89E-13	12.724	glutathione S-transferase, mu 2 (Gstm2)	14863

129	**Atp2a1**	14.54	9680	5.152	35.56	0.8224	4.11E-10	9.386	ATPase, Ca++ transporting, cardiac muscle, fast twitch 1 (Atp2a1)	11937
130	**Cfd**	14.47	679	6.068	67.10	1.0317	3.92E-09	8.407	complement factor D (Cfd)	11537
131	**Hjv**	14.46	140	4.520	22.94	0.6580	1.14E-10	9.942	hemojuvelin BMP co-receptor (Hjv)	69585
132	**Suclg1**	14.29	895	1.064	2.09	0.1362	5.96E-14	13.225	succinate-CoA ligase, GDP-forming, alpha subunit (Suclg1)	56451
133	**Pcolce**	14.25	4559	0.870	1.83	0.1104	4.13E-14	13.384	procollagen C-endopeptidase enhancer protein (Pcolce)	18542
134	**Maged2**	14.11	891	1.044	2.06	0.1346	8.49E-14	13.071	MAGE family member D2 (Maged2)	80884
135	**Hspa5**	14.02	13470	0.804	1.75	0.1024	6.06E-14	13.217	heat shock protein 5 (Hspa5)	14828
136	**C1qc**	14.01	2620	0.906	1.87	0.1163	7.87E-14	13.104	complement component 1, q subcomponent, C chain (C1qc)	12262
137	**Ap2m1**	13.95	3386	0.784	1.72	0.1000	6.80E-14	13.167	adaptor-related protein complex 2, mu 1 subunit (Ap2m1)	11773
138	**Lyve1**	13.89	316	2.375	5.19	0.3334	3.05E-12	11.515	lymphatic vessel endothelial hyaluronan receptor 1 (Lyve1)	114332
139	**Gpx8**	13.85	1151	1.001	2.00	0.1300	1.43E-13	12.845	glutathione peroxidase 8 (putative) (Gpx8)	69590
140	**Nrap**	13.84	873	4.779	27.46	0.7093	8.78E-10	9.056	nebulin-related anchoring protein (Nrap)	18175
141	**C1rb**	13.80	151	2.146	4.43	0.2963	2.21E-12	11.655	complement component 1, r subcomponent B (C1rb)	667277
142	**Hnrnpf**	13.77	3396	0.764	1.70	0.0980	9.88E-14	13.005	heterogeneous nuclear ribonucleoprotein F (Hnrnpf)	98758
143	**Slc25a4**	13.58	5114	0.804	1.75	0.1042	1.69E-13	12.772	solute carrier family 25 (mitochondrial carrier, adenine nucleotide translocator), member 4 (Slc25a4)	11739
144	**Thbs4**	13.54	841	1.596	3.02	0.2187	1.13E-12	11.948	thrombospondin 4 (Thbs4)	21828
145	**H3c2**	13.54	972	1.169	2.25	0.1555	4.22E-13	12.375	H3 clustered histone 2(H3c2)	319150
146	**Acta1**	13.50	5363	5.849	57.63	0.9670	2.24E-08	7.650	actin alpha 1, skeletal muscle (Acta1)	11459
147	**Sec13**	13.42	1315	0.935	1.91	0.1231	3.26E-13	12.487	SEC13 homolog, nuclear pore and COPII coat complex component (Sec13)	110379
148	**Igfbp6**	13.37	169	2.204	4.61	0.3125	6.82E-12	11.166	insulin-like growth factor binding protein 6 (Igfbp6)	16012
149	**Tmem100**	13.36	358	1.676	3.19	0.2302	2.06E-12	11.685	transmembrane protein 100 (Tmem100)	67888
150	**Cox8a**	13.36	1326	0.961	1.95	0.1270	3.98E-13	12.400	cytochrome c oxidase subunit 8A (Cox8a)	12868
151	**Rps23**	13.24	3772	0.769	1.70	0.1007	3.39E-13	12.469	ribosomal protein S23 (Rps23)	66475
152	**H2ac20**	13.22	528	1.206	2.31	0.1628	9.68E-13	12.014	H2A clustered histone 20 (H2ac20)	319176

153	**Fmod**	13.21	626	1.861	3.63	0.2621	4.47E-12	11.349	Fibromodulin (Fmod)	14264
154	**Ccn3**	13.16	244	1.778	3.43	0.2490	4.11E-12	11.386	cellular communication network factor 3 (Ccn3)	18133
155	**Ccl21b**	13.16	129	2.672	6.37	0.3966	3.22E-11	10.492	C-C motif chemokine ligand 21B (leucine) (Ccl21b)	10004249<br>3
156	**Gm13304**	13.14	128	2.666	6.35	0.3960	3.36E-11	10.474	No gene name found in the DAVID Knowledgebase	0
157	**Cycs**	13.07	923	1.002	2.00	0.1344	8.47E-13	12.072	cytochrome c, somatic (Cycs)	13063
158	**Wfdc17**	13.04	309	1.806	3.50	0.2534	5.86E-12	11.232	WAP four-disulfide core domain 17 (Wfdc17)	10003425<br>1
159	**Folr2**	13.02	123	2.634	6.21	0.3894	4.15E-11	10.382	folate receptor beta (Folr2)	14276
160	**Gm12033**	12.95	714	1.177	2.26	0.1606	1.67E-12	11.778	predicted gene 12033 (Gm12033)	10004274<br>6
161	**Rpl35a**	12.91	2415	0.903	1.87	0.1211	9.77E-13	12.010	ribosomal protein L35A (Rpl35a)	57808
162	**Eif3l**	12.91	2563	0.780	1.72	0.1037	7.39E-13	12.131	eukaryotic translation initiation factor 3, subunit L (Eif3l)	223691
163	**Psmb2**	12.89	1196	0.955	1.94	0.1287	1.16E-12	11.936	proteasome (prosome, macropain) subunit, beta type 2 (Psmb2)	26445
164	**Gm7336**	12.79	1000	1.052	2.07	0.1434	1.82E-12	11.739	predicted gene 7336 (Gm7336)	654473
165	**Cd302**	12.78	280	1.707	3.26	0.2414	8.46E-12	11.073	CD302 antigen (Cd302)	66205
166	**Dpep1**	12.74	149	2.367	5.16	0.3508	4.19E-11	10.377	dipeptidase 1 (Dpep1)	13479
167	**Ecrg4**	12.66	220	2.386	5.23	0.3537	5.28E-11	10.278	ECRG4 augurin precursor (Ecrg4)	78896
168	**Lrg1**	12.66	309	1.484	2.80	0.2090	6.73E-12	11.172	leucine-rich alpha-2-glycoprotein 1 (Lrg1)	76905
169	**Mybpc2**	12.64	2537	4.217	18.60	0.7156	3.74E-09	8.427	myosin binding protein C, fast-type (Mybpc2)	233199
170	**Cd34**	12.59	2239	0.938	1.92	0.1277	2.24E-12	11.649	CD34 antigen (Cd34)	12490
171	**H3c7**	12.57	973	1.006	2.01	0.1383	2.76E-12	11.560	H3 clustered histone 7 (H3c7)	260423
172	**LOC10816<br>7922**	12.48	401	1.299	2.46	0.1826	6.61E-12	11.180	ribosomal protein L27 pseudogene (LOC108167922)	10816792<br>2
173	**Rpl13**	12.44	4482	0.777	1.71	0.1055	2.18E-12	11.662	ribosomal protein L13 (Rpl13)	270106
174	**Cacng1**	12.40	82	4.221	18.65	0.6870	6.61E-09	8.180	calcium channel, voltage-dependent, gamma subunit 1 (Cacng1)	12299
175	**H2bc8**	12.40	591	1.121	2.17	0.1563	5.30E-12	11.276	H2B clustered histone 8 (H2bc8)	319181
176	**Fgl2**	12.40	709	1.234	2.35	0.1733	6.90E-12	11.161	fibrinogen-like protein 2 (Fgl2)	14190
177	**Sec61g**	12.36	802	1.179	2.26	0.1651	6.58E-12	11.182	SEC61 translocon subunit gamma (Sec61g)	20335
178	**H1f2**	12.34	1723	0.929	1.90	0.1282	3.92E-12	11.406	H1.2 linker histone, cluster member (H1f2)	50708

179	**Reln**	12.21	256	1.787	3.45	0.2630	3.76E-11	10.424	Reelin (Reln)	19699
180	**Psmc3**	12.20	2200	0.786	1.72	0.1079	3.82E-12	11.418	proteasome (prosome, macropain) 26S subunit, ATPase 3 (Psmc3)	19182
181	**Fstl1**	12.15	16616	1.301	2.46	0.1858	1.41E-11	10.851	follistatin-like 1 (Fstl1)	14314
182	**Gm13394**	12.15	702	1.152	2.22	0.1631	1.01E-11	10.994	predicted gene 13394 (Gm13394)	100033452
183	**Olfml3**	12.08	981	1.335	2.52	0.1917	1.82E-11	10.740	olfactomedin-like 3 (Olfml3)	99543
184	**Mylpf**	11.93	1453	5.081	33.84	0.9802	1.40E-07	6.853	myosin light chain, phosphorylatable, fast skeletal muscle (Mylpf)	17907
185	**Rpl34**	11.93	2856	0.886	1.85	0.1242	9.12E-12	11.040	ribosomal protein L34 (Rpl34)	68436
186	**F5**	11.92	342	1.465	2.76	0.2138	3.48E-11	10.458	coagulation factor V (F5)	14067
187	**Abca8a**	11.78	204	2.984	7.91	0.4935	1.61E-09	8.794	ATP-binding cassette, sub-family A member 8a (Abca8a)	217258
188	**Itgbl1**	11.78	275	2.188	4.56	0.3359	2.58E-10	9.589	integrin, beta-like 1 (Itgbl1)	223272
189	**Sod1**	11.76	1460	0.883	1.84	0.1249	1.33E-11	10.877	superoxide dismutase 1, soluble (Sod1)	20655
190	**Rps19**	11.76	2682	0.815	1.76	0.1146	1.14E-11	10.941	ribosomal protein S19 (Rps19)	20085
191	**Rpl27-ps3**	11.65	331	1.329	2.51	0.1952	4.81E-11	10.318	ribosomal protein L27, pseudogene 3 (Rpl27-ps3)	621100
192	**Ccl21d**	11.54	103	2.644	6.25	0.4304	1.26E-09	8.898	C-C motif chemokine ligand 21D (Ccl21d)	100862177
193	**Hmox2**	11.51	605	1.099	2.14	0.1600	3.91E-11	10.408	heme oxygenase 2 (Hmox2)	15369
194	**Gm10591**	11.49	104	2.622	6.16	0.4274	1.35E-09	8.870	No gene name found in the DAVID Knowledgebase	0
195	**Sod3**	11.49	1224	1.015	2.02	0.1468	3.36E-11	10.474	superoxide dismutase 3, extracellular (Sod3)	20657
196	**Atp5pb**	11.48	2468	0.861	1.82	0.1234	2.40E-11	10.620	ATP synthase peripheral stalk-membrane subunit b (Atp5pb)	11950
197	**Bmper**	11.46	169	1.875	3.67	0.2876	2.62E-10	9.581	BMP-binding endothelial regulator (Bmper)	73230
198	**Klhl31**	11.42	473	5.255	38.19	0.9980	6.84E-07	6.165	kelch-like 31 (Klhl31)	244923
199	**Ssr4**	11.41	1124	1.008	2.01	0.1465	3.98E-11	10.400	signal sequence receptor, delta (Ssr4)	20832
200	**Ndufb1**	11.29	681	1.176	2.26	0.1744	7.62E-11	10.118	NADH:ubiquinone oxidoreductase subunit B1 (Ndufb1)	102631912
201	**Myh4**	11.25	18695	5.969	62.66	1.3158	5.30E-06	5.276	myosin, heavy polypeptide 4, skeletal muscle (Myh4)	17884
202	**H2ac24**	11.23	470	1.210	2.31	0.1803	9.57E-11	10.019	H2A clustered histone 24 (H2ac24)	319171
203	**Lrrc17**	11.21	305	1.766	3.40	0.2730	3.61E-10	9.442	leucine rich repeat containing 17 (Lrrc17)	74511
204	**Ctsd**	11.19	14426	0.855	1.81	0.1243	4.68E-11	10.330	cathepsin D (Ctsd)	13033

205	**Rpl27-ps2**	11.17	163	1.827	3.55	0.2853	4.49E-10	9.347	ribosomal protein L27, pseudogene 2 (Rpl27-ps2)	194960
206	**Akr1b8**	11.13	886	1.041	2.06	0.1538	8.07E-11	10.093	aldo-keto reductase family 1, member B8 (Akr1b8)	14187
207	**Myh8**	11.13	594	5.142	35.30	0.9818	1.04E-06	5.985	myosin, heavy polypeptide 8, skeletal muscle, perinatal (Myh8)	17885
208	**C3ar1**	11.10	1334	0.827	1.77	0.1204	5.39E-11	10.268	complement component 3a receptor 1 (C3ar1)	12267
209	**Myh1**	11.02	1409	4.936	30.61	1.0022	8.23E-07	6.084	myosin, heavy polypeptide 1, skeletal muscle, adult (Myh1)	17879
210	**Ccl11**	10.99	399	2.213	4.64	0.3580	1.68E-09	8.774	C-C motif chemokine ligand 11 (Ccl11)	20292
211	**Timp1**	10.92	1993	1.034	2.05	0.1543	1.29E-10	9.889	tissue inhibitor of metalloproteinase 1 (Timp1)	21857
212	**Rps27**	10.88	2079	0.967	1.96	0.1441	1.22E-10	9.913	ribosomal protein S27 (Rps27)	57294
213	**P4ha1**	10.83	1569	0.791	1.73	0.1163	9.09E-11	10.041	procollagen-proline, 2-oxoglutarate 4-dioxygenase (proline 4-hydroxylase), alpha 1 polypeptide (P4ha1)	18451
214	**Cxcl12**	10.81	1600	1.385	2.61	0.2140	3.73E-10	9.428	C-X-C motif chemokine ligand 12 (Cxcl12)	20315
215	**Drp2**	10.81	180	1.937	3.83	0.3131	1.34E-09	8.872	dystrophin related protein 2 (Drp2)	13497
216	**Gm2451**	10.72	530	1.153	2.22	0.1760	2.74E-10	9.563	predicted pseudogene 2451 (Gm2451)	100039840
217	**Neb**	10.70	12687	3.923	15.17	0.7599	1.69E-07	6.773	Nebulin (Neb)	17996
218	**Gm14760**	10.70	502	1.201	2.30	0.1842	3.19E-10	9.496	predicted gene 14760 (Gm14760)	654474
219	**Rarres2**	10.69	145	2.606	6.09	0.4434	8.24E-09	8.084	retinoic acid receptor responder (tazarotene induced) 2 (Rarres2)	71660
220	**Fcna**	10.64	96	2.259	4.79	0.3754	4.11E-09	8.386	ficolin A (Fcna)	14133
221	**Gm12671**	10.61	562	1.124	2.18	0.1722	3.29E-10	9.483	predicted gene 12671 (Gm12671)	654475
222	**Adgre1**	10.60	819	0.951	1.93	0.1434	2.24E-10	9.650	adhesion G protein-coupled receptor E1 (Adgre1)	13733
223	**Slc43a3**	10.56	335	1.459	2.75	0.2303	7.91E-10	9.102	solute carrier family 43, member 3 (Slc43a3)	58207
224	**Dbi**	10.56	638	1.191	2.28	0.1843	4.27E-10	9.370	diazepam binding inhibitor (Dbi)	13167
225	**Angptl4**	10.56	883	0.926	1.90	0.1399	2.35E-10	9.629	angiopoietin-like 4 (Angptl4)	57875
226	**H2-Aa**	10.48	2230	0.946	1.93	0.1442	2.95E-10	9.530	histocompatibility 2, class II antigen A, alpha (H2-Aa)	14960
227	**Cmya5**	10.47	2659	3.820	14.12	0.7494	2.23E-07	6.652	cardiomyopathy associated 5 (Cmya5)	76469
228	**Gm22220**	10.46	157	1.974	3.93	0.3257	3.30E-09	8.481	predicted gene, 22220 (Gm22220)	115488081
229	**Cacna1s**	10.44	455	4.207	18.46	0.8206	5.82E-07	6.235	calcium channel, voltage-dependent, L type, alpha 1S subunit (Cacna1s)	12292

230	**Slfn5**	10.42	1708	0.878	1.84	0.1332	2.88E-10	9.541	schlafen 5 (Slfn5)	327978
231	**1110004F10Rik**	10.41	789	0.971	1.96	0.1485	3.60E-10	9.443	RIKEN cDNA 1110004F10 gene (1110004F10Rik)	56372
232	**Ifi27l2a**	10.38	276	1.867	3.65	0.3090	3.04E-09	8.517	interferon, alpha-inducible protein 27 like 2A (Ifi27l2a)	76933
233	**Gm2574**	10.36	495	1.096	2.14	0.1702	5.39E-10	9.268	predicted pseudogene 2574 (Gm2574)	100040053
234	**Cops4**	10.33	1010	0.887	1.85	0.1353	3.59E-10	9.445	COP9 signalosome subunit 4 (Cops4)	26891
235	**Tnnt3**	10.33	2957	3.993	15.92	0.8082	4.62E-07	6.335	troponin T3, skeletal, fast (Tnnt3)	21957
236	**Fbxo40**	10.28	185	2.936	7.65	0.5277	4.52E-08	7.345	F-box protein 40 (Fbxo40)	207215
237	**Gm10359**	10.27	566	1.134	2.20	0.1777	7.37E-10	9.133	predicted gene 10359 (Gm10359)	100042349
238	**Tma7**	10.24	756	0.933	1.91	0.1438	4.93E-10	9.307	translational machinery associated 7 (Tma7)	66167
239	**Cd163**	10.21	481	1.467	2.76	0.2374	1.81E-09	8.743	CD163 antigen (Cd163)	93671
240	**Kng2**	10.14	189	2.083	4.24	0.3520	8.73E-09	8.059	kininogen 2 (Kng2)	385643
241	**Ryr1**	10.14	3323	3.789	13.83	0.7657	4.48E-07	6.349	ryanodine receptor 1, skeletal muscle (Ryr1)	20190
242	**Psma3**	10.12	2129	0.814	1.76	0.1252	4.93E-10	9.307	proteasome subunit alpha 3 (Psma3)	19167
243	**Gm12537**	10.11	541	1.170	2.25	0.1857	1.16E-09	8.936	predicted gene 12537 (Gm12537)	100041342
244	**Cox7a2**	10.08	693	1.035	2.05	0.1624	9.00E-10	9.046	cytochrome c oxidase subunit 7A2 (Cox7a2)	12866
245	**Capg**	10.07	3910	0.771	1.71	0.1180	5.01E-10	9.300	capping actin protein, gelsolin like (Capg)	12332
246	**Il33**	10.04	1933	0.991	1.99	0.1553	9.03E-10	9.044	interleukin 33 (Il33)	77125
247	**Gm3222**	10.03	256	1.405	2.65	0.2282	2.36E-09	8.626	predicted pseudogene 3222 (Gm3222)	100041236
248	**Tomm7**	10.01	581	1.061	2.09	0.1679	1.13E-09	8.947	translocase of outer mitochondrial membrane 7 (Tomm7)	66169
249	**Pde4b**	9.99	683	1.199	2.30	0.1921	1.61E-09	8.792	phosphodiesterase 4B, cAMP specific (Pde4b)	18578
250	**Gm15772**	9.98	451	1.090	2.13	0.1729	1.30E-09	8.886	predicted gene 15772 (Gm15772)	100034726
251	**Acsl1**	9.95	555	1.222	2.33	0.1962	1.88E-09	8.725	acyl-CoA synthetase long-chain family member 1 (Acsl1)	14081
252	**Pi16**	9.93	879	2.706	6.52	0.4964	5.95E-08	7.226	peptidase inhibitor 16 (Pi16)	74116
253	**Cadm3**	9.90	135	2.604	6.08	0.4789	5.06E-08	7.296	cell adhesion molecule 3 (Cadm3)	94332
254	**Galnt16**	9.88	203	1.804	3.49	0.3078	8.41E-09	8.075	polypeptide N-acetylgalactosaminyltransferase 16 (Galnt16)	108760
255	**Myoz1**	9.87	346	5.424	42.95	1.3123	3.61E-05	4.443	myozenin 1 (Myoz1)	59011

256	**Ndufa9**	9.84	686	1.001	2.00	0.1589	1.45E-09	8.838	NADH:ubiquinone oxidoreductase subunit A9 (Ndufa9)	66108
257	**Arpc3**	9.81	1537	1.126	2.18	0.1817	2.09E-09	8.679	actin related protein 2/3 complex, subunit 3 (Arpc3)	56378
258	**Slc38a4**	9.79	104	3.106	8.61	0.5962	2.05E-07	6.688	solute carrier family 38, member 4 (Slc38a4)	69354
259	**Col3a1**	9.79	76595	1.089	2.13	0.1746	2.00E-09	8.700	collagen, type III, alpha 1 (Col3a1)	12825
260	**Prl2c2**	9.78	3159	1.326	2.51	0.2173	3.48E-09	8.458	prolactin family 2, subfamily c, member 2 (Prl2c2)	18811
261	**Ucp3**	9.76	82	4.150	17.76	0.8392	2.48E-06	5.605	uncoupling protein 3 (mitochondrial, proton carrier) (Ucp3)	22229
262	**Mybpc1**	9.74	578	5.132	35.06	1.2544	2.48E-05	4.606	myosin binding protein C, slow-type (Mybpc1)	109272
263	**Rpl10-ps3**	9.72	823	0.897	1.86	0.1421	1.51E-09	8.820	ribosomal protein L10, pseudogene 3 (Rpl10-ps3)	10004334<br>6
264	**Cd63**	9.65	5337	0.795	1.74	0.1250	1.39E-09	8.858	CD63 antigen (Cd63)	12512
265	**Ugt1a7c**	9.61	404	1.272	2.42	0.2099	4.57E-09	8.340	UDP glucuronosyltransferase 1 family, polypeptide A7C (Ugt1a7c)	394432
266	**Cfp**	9.59	997	0.968	1.96	0.1561	2.36E-09	8.626	complement factor properdin (Cfp)	18636
267	**Clec3b**	9.59	118	3.137	8.79	0.6302	3.49E-07	6.457	C-type lectin domain family 3, member b (Clec3b)	21922
268	**Fap**	9.58	713	0.957	1.94	0.1538	2.36E-09	8.626	fibroblast activation protein (Fap)	14089
269	**Serinc1**	9.54	5052	0.911	1.88	0.1461	2.36E-09	8.626	serine incorporator 1 (Serinc1)	56442
270	**Fxyd6**	9.53	319	1.300	2.46	0.2169	5.91E-09	8.228	FXYD domain-containing ion transport regulator 6 (Fxyd6)	59095
271	**Bche**	9.52	206	1.698	3.24	0.2931	1.52E-08	7.819	Butyrylcholinesterase (Bche)	12038
272	**Medag**	9.49	1495	1.016	2.02	0.1651	3.39E-09	8.470	mesenteric estrogen dependent adipogenesis (Medag)	70717
273	**Psmc4**	9.44	1118	0.813	1.76	0.1299	2.35E-09	8.628	proteasome (prosome, macropain) 26S subunit, ATPase, 4 (Psmc4)	23996
274	**Glipr2**	9.43	487	1.026	2.04	0.1676	3.92E-09	8.407	GLI pathogenesis-related 2 (Glipr2)	384009
275	**Upp1**	9.40	429	1.413	2.66	0.2396	1.04E-08	7.984	uridine phosphorylase 1 (Upp1)	22271
276	**Pdgfrl**	9.35	239	1.965	3.90	0.3511	4.08E-08	7.389	platelet-derived growth factor receptor-like (Pdgfrl)	68797
277	**Fcgr3**	9.28	1832	0.876	1.84	0.1427	3.93E-09	8.406	Fc receptor, IgG, low affinity III (Fcgr3)	14131
278	**Cthrc1**	9.23	547	1.443	2.72	0.2482	1.64E-08	7.786	collagen triple helix repeat containing 1 (Cthrc1)	68588
279	**Gm10136**	9.22	155	1.703	3.26	0.3005	3.03E-08	7.519	predicted pseudogene 10136 (Gm10136)	672214
280	**Trdn**	9.19	611	4.302	19.73	0.9700	1.30E-05	4.887	Triadin (Trdn)	76757
281	**Fcrl2**	9.18	491	1.011	2.02	0.1677	6.74E-09	8.171	Fc receptor like 2 (Fcrl2)	80891
282	**Cpxm2**	9.18	1395	0.890	1.85	0.1461	5.18E-09	8.285	carboxypeptidase X, M14 family member 2 (Cpxm2)	55987

283	**Sec61b**	9.17	797	0.902	1.87	0.1481	5.34E-09	8.273	SEC61 translocon subunit beta (Sec61b)	66212
284	**Aqp1**	9.12	1961	1.140	2.20	0.1922	1.04E-08	7.982	aquaporin 1 (Aqp1)	11826
285	**Bicc1**	9.08	1272	0.772	1.71	0.1257	4.89E-09	8.311	BicC family RNA binding protein 1 (Bicc1)	83675
286	**Myh2**	9.08	626	4.814	28.13	1.1671	5.43E-05	4.265	myosin, heavy polypeptide 2, skeletal muscle, adult (Myh2)	17882
287	**Mfap2**	9.07	292	1.388	2.62	0.2409	2.08E-08	7.682	microfibrillar-associated protein 2 (Mfap2)	17150
288	**Col11a1**	9.06	563	1.535	2.90	0.2700	2.97E-08	7.527	collagen, type XI, alpha 1 (Col11a1)	12814
289	**Ednrb**	9.05	625	1.080	2.11	0.1826	1.07E-08	7.972	endothelin receptor type B (Ednrb)	13618
290	**Apoe**	9.05	14306	0.790	1.73	0.1292	5.47E-09	8.262	apolipoprotein E (Apoe)	11816
291	**Dpp4**	9.03	203	1.652	3.14	0.2937	4.14E-08	7.383	dipeptidylpeptidase 4 (Dpp4)	13482
292	**Gm4609**	9.03	243	1.350	2.55	0.2343	2.10E-08	7.679	predicted gene 4609 (Gm4609)	10004372<br>4
293	**Gm9396**	9.02	227	1.332	2.52	0.2307	2.04E-08	7.691	predicted gene 9396 (Gm9396)	668844
294	**H3c15**	8.98	852	0.964	1.95	0.1616	9.70E-09	8.013	H3 clustered histone 15 (H3c15)	97114
295	**Pvalb**	8.96	1596	5.300	39.38	1.4911	2.17E-04	3.664	Parvalbumin (Pvalb)	19293
296	**Pcna**	8.95	1351	0.789	1.73	0.1299	6.89E-09	8.162	proliferating cell nuclear antigen (Pcna)	18538
297	**Polr2j**	8.89	354	1.147	2.21	0.1970	1.79E-08	7.747	polymerase (RNA) II (DNA directed) polypeptide J (Polr2j)	20022
298	**Selenop**	8.89	3857	1.188	2.28	0.2048	2.00E-08	7.700	selenoprotein P (Selenop)	20363
299	**Gm10291**	8.88	183	1.473	2.78	0.2619	3.90E-08	7.409	predicted pseudogene 10291 (Gm10291)	10004174<br>8
300	**Arsi**	8.88	271	1.430	2.69	0.2523	3.54E-08	7.451	arylsulfatase I (Arsi)	545260
301	**Ctsb**	8.88	14110	0.855	1.81	0.1421	9.43E-09	8.026	cathepsin B (Ctsb)	13030
302	**Hexb**	8.87	963	0.804	1.75	0.1333	8.59E-09	8.066	hexosaminidase B (Hexb)	15212
303	**Cox5b**	8.83	872	0.818	1.76	0.1361	9.66E-09	8.015	cytochrome c oxidase subunit 5B (Cox5b)	12859
304	**H3c4**	8.81	722	0.981	1.97	0.1668	1.49E-08	7.827	H3 clustered histone 4 (H3c4)	319149
305	**Ppp1r3a**	8.68	558	3.875	14.67	0.9460	1.57E-05	4.805	protein phosphatase 1, regulatory subunit 3A (Ppp1r3a)	140491
306	**Kcnj2**	8.68	593	0.949	1.93	0.1621	1.86E-08	7.731	potassium inwardly-rectifying channel, subfamily J, member 2 (Kcnj2)	16518
307	**Mitf**	8.67	297	1.309	2.48	0.2334	4.38E-08	7.359	melanogenesis associated transcription factor (Mitf)	17342
308	**Plat**	8.64	2374	1.064	2.09	0.1846	2.65E-08	7.577	plasminogen activator, tissue (Plat)	18791

309	**H6pd**	8.64	1114	0.772	1.71	0.1293	1.35E-08	7.869	hexose-6-phosphate dehydrogenase (glucose 1-dehydrogenase) (H6pd)	100198
310	**Tcn2**	8.60	898	0.802	1.74	0.1354	1.58E-08	7.802	transcobalamin 2 (Tcn2)	21452
311	**H1f4**	8.53	1476	0.771	1.71	0.1303	1.72E-08	7.763	H1.4 linker histone, cluster member (H1f4)	50709
312	**Gpd1**	8.52	302	3.366	10.31	0.7780	7.09E-06	5.149	glycerol-3-phosphate dehydrogenase 1 (soluble) (Gpd1)	14555
313	**Meox2**	8.51	140	1.660	3.16	0.3100	1.41E-07	6.852	mesenchyme homeobox 2 (Meox2)	17286
314	**Igfbp5**	8.47	5036	1.112	2.16	0.1964	4.36E-08	7.360	insulin-like growth factor binding protein 5 (Igfbp5)	16011
315	**Rpl15-ps3**	8.47	959	0.776	1.71	0.1319	2.05E-08	7.689	ribosomal protein L15, pseudogene 3 (Rpl15-ps3)	10003472<br>4
316	**Penk**	8.46	364	1.252	2.38	0.2258	6.23E-08	7.206	Preproenkephalin (Penk)	18619
317	**Uqcrb**	8.38	859	0.800	1.74	0.1372	2.61E-08	7.583	ubiquinol-cytochrome c reductase binding protein (Uqcrb)	67530
318	**Cd52**	8.37	423	1.215	2.32	0.2195	6.95E-08	7.158	CD52 antigen (Cd52)	23833
319	**Art3**	8.33	133	2.512	5.70	0.5239	1.52E-06	5.819	ADP-ribosyltransferase 3 (Art3)	109979
320	**Car3**	8.31	2103	4.047	16.53	1.0761	5.46E-05	4.263	carbonic anhydrase 3 (Car3)	12350
321	**Prl2c3**	8.22	3262	1.184	2.27	0.2151	9.31E-08	7.031	prolactin family 2, subfamily c, member 3 (Prl2c3)	18812
322	**Scara3**	8.19	508	1.424	2.68	0.2661	1.73E-07	6.763	scavenger receptor class A, member 3 (Scara3)	219151
323	**H2ac11**	8.18	354	1.128	2.19	0.2047	8.90E-08	7.051	H2A clustered histone 11 (H2ac11)	319167
324	**Myl9**	8.13	357	1.148	2.22	0.2094	1.03E-07	6.986	myosin, light polypeptide 9, regulatory (Myl9)	98932
325	**Ebf2**	8.13	215	1.755	3.37	0.3452	4.20E-07	6.377	early B cell factor 2 (Ebf2)	13592
326	**Lpar1**	8.13	898	0.826	1.77	0.1447	4.96E-08	7.305	lysophosphatidic acid receptor 1 (Lpar1)	14745
327	**Asns**	8.11	470	1.028	2.04	0.1851	8.34E-08	7.079	asparagine synthetase (Asns)	27053
328	**H2ac12**	8.11	348	1.098	2.14	0.1996	9.83E-08	7.007	H2A clustered histone 12 (H2ac12)	319168
329	**Rpl18**	8.10	3658	0.753	1.69	0.1313	4.48E-08	7.349	ribosomal protein L18 (Rpl18)	19899
330	**Casq1**	8.10	663	3.838	14.30	1.0331	5.50E-05	4.260	calsequestrin 1 (Casq1)	12372
331	**Uqcrc2**	8.07	1520	0.823	1.77	0.1449	5.71E-08	7.243	ubiquinol cytochrome c reductase core protein 2 (Uqcrc2)	67003
332	**Rbmxl1**	8.03	898	0.769	1.70	0.1349	5.44E-08	7.265	RNA binding motif protein, X-linked like-1 (Rbmxl1)	19656
333	**Cst3**	8.02	2917	0.942	1.92	0.1690	8.37E-08	7.077	cystatin C (Cst3)	13010
334	**Rpl10-ps6**	7.94	478	0.933	1.91	0.1685	9.74E-08	7.011	ribosomal protein L10, pseudogene 6 (Rpl10-ps6)	10004301<br>0
335	**Ttn**	7.91	81195	3.556	11.76	0.9370	4.41E-05	4.356	Titin (Ttn)	22138

336	**Obscn**	7.87	1929	3.442	10.87	0.8856	3.70E-05	4.432	obscurin, cytoskeletal calmodulin and titin-interacting RhoGEF (Obscn)	380698
337	**Asb11**	7.83	82	3.573	11.90	0.8224	5.54E-05	4.256	ankyrin repeat and SOCS box-containing 11 (Asb11)	68854
338	**H3c11**	7.82	441	0.988	1.98	0.1817	1.48E-07	6.829	H3 clustered histone 11 (H3c11)	319153
339	**Tmem45a**	7.81	188	1.468	2.77	0.2852	4.53E-07	6.344	transmembrane protein 45a (Tmem45a)	56277
340	**C1s2**	7.79	127	1.742	3.35	0.3503	8.92E-07	6.050	complement component 1, s subcomponent 2 (C1s2)	317677
341	**Cdh11**	7.79	2762	0.977	1.97	0.1796	1.55E-07	6.809	cadherin 11 (Cdh11)	12552
342	**Fkbp7**	7.78	271	1.184	2.27	0.2235	2.52E-07	6.599	FK506 binding protein 7 (Fkbp7)	14231
343	**Rbfox1**	7.78	150	2.323	5.01	0.4982	3.51E-06	5.455	RNA binding protein, fox-1 homolog (C. elegans) 1 (Rbfox1)	268859
344	**H2ac6**	7.74	253	1.205	2.31	0.2291	2.91E-07	6.537	H2A clustered histone 6 (H2ac6)	319164
345	**Mpc1**	7.74	423	1.126	2.18	0.2119	2.46E-07	6.609	mitochondrial pyruvate carrier 1 (Mpc1)	55951
346	**Ech1**	7.73	662	0.846	1.80	0.1536	1.32E-07	6.879	enoyl coenzyme A hydratase 1, peroxisomal (Ech1)	51798
347	**Col28a1**	7.72	455	1.328	2.51	0.2558	4.03E-07	6.395	collagen, type XXVIII, alpha 1 (Col28a1)	213945
348	**Ndufb4**	7.72	522	0.901	1.87	0.1649	1.53E-07	6.815	NADH:ubiquinone oxidoreductase subunit B4 (Ndufb4)	68194
349	**Rpl37**	7.71	1959	0.924	1.90	0.1702	1.63E-07	6.788	ribosomal protein L37 (Rpl37)	67281
350	**Selp**	7.71	167	1.419	2.67	0.2767	5.12E-07	6.291	selectin, platelet (Selp)	20344
351	**Thbs2**	7.70	9028	0.950	1.93	0.1753	1.80E-07	6.745	thrombospondin 2 (Thbs2)	21826
352	**Rragd**	7.68	154	2.288	4.88	0.4990	4.06E-06	5.392	Ras-related GTP binding D (Rragd)	52187
353	**Ndufa12**	7.66	948	0.811	1.75	0.1473	1.40E-07	6.853	NADH:ubiquinone oxidoreductase subunit A12 (Ndufa12)	66414
354	**Mylk4**	7.64	786	4.377	20.78	1.6708	5.45E-04	3.264	myosin light chain kinase family, member 4 (Mylk4)	238564
355	**Cpxm1**	7.64	732	1.386	2.61	0.2719	5.59E-07	6.252	carboxypeptidase X, M14 family member 1 (Cpxm1)	56264
356	**Tubb2a**	7.63	950	0.757	1.69	0.1368	1.33E-07	6.877	tubulin, beta 2A class IIA (Tubb2a)	22151
357	**Kcne4**	7.60	142	1.664	3.17	0.3401	1.15E-06	5.940	potassium voltage-gated channel, Isk-related subfamily, gene 4 (Kcne4)	57814
358	**Ndufs5**	7.56	332	1.056	2.08	0.2001	3.10E-07	6.508	NADH:ubiquinone oxidoreductase core subunit S5 (Ndufs5)	595136
359	**Alpk3**	7.56	152	2.610	6.11	0.6019	1.12E-05	4.950	alpha-kinase 3 (Alpk3)	116904
360	**Tmod1**	7.54	89	2.461	5.50	0.5609	8.39E-06	5.076	tropomodulin 1 (Tmod1)	21916
361	**Lama4**	7.52	2464	0.838	1.79	0.1545	2.09E-07	6.680	laminin, alpha 4 (Lama4)	16775

362	**Eif3e**	7.44	3210	0.786	1.72	0.1451	2.23E-07	6.653	eukaryotic translation initiation factor 3, subunit E (Eif3e)	16341
363	**Sparc**	7.43	41581	0.873	1.83	0.1629	2.77E-07	6.557	secreted acidic cysteine rich glycoprotein (Sparc)	20692
364	**B3galnt1**	7.41	177	1.662	3.17	0.3442	1.80E-06	5.745	UDP-GalNAc:betaGlcNAc beta 1,3-galactosaminyltransferase, polypeptide 1 (B3galnt1)	26879
365	**Ninj1**	7.38	692	0.895	1.86	0.1686	3.31E-07	6.480	ninjurin 1 (Ninj1)	18081
366	**Ms4a4d**	7.35	198	1.951	3.87	0.4223	3.96E-06	5.403	membrane-spanning 4-domains, subfamily A, member 4D (Ms4a4d)	66607
367	**Ndufa4**	7.33	659	0.886	1.85	0.1676	3.59E-07	6.445	Ndufa4, mitochondrial complex associated (Ndufa4)	17992
368	**Pygm**	7.33	2114	3.630	12.38	1.1101	1.99E-04	3.702	muscle glycogen phosphorylase (Pygm)	19309
369	**Plbd1**	7.32	460	0.978	1.97	0.1877	4.57E-07	6.340	phospholipase B domain containing 1 (Plbd1)	66857
370	**H3c3**	7.31	781	0.851	1.80	0.1602	3.44E-07	6.464	H3 clustered histone 3 (H3c3)	319148
371	**Gm10358**	7.31	294	1.130	2.19	0.2213	6.56E-07	6.183	No gene name found in the DAVID Knowledgebase	0
372	**Mme**	7.28	303	1.450	2.73	0.2966	1.47E-06	5.831	membrane metallo endopeptidase (Mme)	17380
373	**Ackr3**	7.28	935	0.765	1.70	0.1426	3.05E-07	6.515	atypical chemokine receptor 3 (Ackr3)	12778
374	**Sfrp2**	7.26	1212	1.313	2.48	0.2645	1.12E-06	5.952	secreted frizzled-related protein 2 (Sfrp2)	20319
375	**H3c6**	7.25	806	0.854	1.81	0.1619	4.03E-07	6.395	H3 clustered histone 6 (H3c6)	319151
376	**Ost4**	7.24	652	0.842	1.79	0.1592	4.03E-07	6.395	oligosaccharyltransferase complex subunit 4 (non-catalytic) (Ost4)	67695
377	**H2ac7**	7.23	241	1.134	2.19	0.2238	8.00E-07	6.097	H2A clustered histone 7 (H2ac7)	319165
378	**Adam23**	7.20	97	1.719	3.29	0.3666	3.30E-06	5.481	a disintegrin and metallopeptidase domain 23 (Adam23)	23792
379	**Ldb3**	7.16	544	3.319	9.98	0.9636	1.44E-04	3.841	LIM domain binding 3 (Ldb3)	24131
380	**Micos13**	7.15	324	1.090	2.13	0.2154	8.74E-07	6.059	mitochondrial contact site and cristae organizing system subunit 13 (Micos13)	224904
381	**Mcub**	7.13	361	1.076	2.11	0.2126	8.87E-07	6.052	mitochondrial calcium uniporter dominant negative beta subunit (Mcub)	66815
382	**Polr2e**	7.11	739	0.789	1.73	0.1499	4.74E-07	6.325	polymerase (RNA) II (DNA directed) polypeptide E (Polr2e)	66420
383	**Eya4**	7.10	156	1.632	3.10	0.3498	3.39E-06	5.470	EYA transcriptional coactivator and phosphatase 4 (Eya4)	14051
384	**Ptn**	7.10	619	0.847	1.80	0.1622	5.59E-07	6.253	Pleiotrophin (Ptn)	19242
385	**H2ac23**	7.10	283	1.077	2.11	0.2138	9.53E-07	6.021	H2A clustered histone 23 (H2ac23)	665433

386	**Cmah**	7.08	359	1.304	2.47	0.2695	1.68E-06	5.775	cytidine monophospho-N-acetylneuraminic acid hydroxylase (Cmah)	12763
387	**Hsd17b10**	7.06	521	0.894	1.86	0.1735	6.81E-07	6.167	hydroxysteroid (17-beta) dehydrogenase 10 (Hsd17b10)	15108
388	**Col6a1**	7.05	10402	0.805	1.75	0.1539	5.64E-07	6.249	collagen, type VI, alpha 1 (Col6a1)	12833
389	**Gm10293**	7.05	261	1.096	2.14	0.2190	1.11E-06	5.957	predicted pseudogene 10293 (Gm10293)	100039762
390	**Gm13292**	7.05	416	0.983	1.98	0.1935	8.53E-07	6.069	predicted gene 13292 (Gm13292)	100040898
391	**Gm8797**	7.04	180	1.253	2.38	0.2565	1.64E-06	5.786	predicted pseudogene 8797 (Gm8797)	667759
392	**Cd63-ps**	7.02	467	1.111	2.16	0.2233	1.25E-06	5.904	CD63 antigen, pseudogene (Cd63-ps)	626721
393	**Gm5138**	6.99	323	1.045	2.06	0.2090	1.14E-06	5.942	predicted gene 5138 (Gm5138)	380687
394	**Pros1**	6.97	1641	0.960	1.94	0.1893	9.68E-07	6.014	protein S (alpha) (Pros1)	19128
395	**Scn4a**	6.97	307	3.255	9.55	0.9580	1.92E-04	3.716	sodium channel, voltage-gated, type IV, alpha(Scn4a)	110880
396	**Itih5**	6.97	1060	0.874	1.83	0.1710	8.09E-07	6.092	inter-alpha-trypsin inhibitor, heavy chain 5 (Itih5)	209378
397	**Myom2**	6.94	627	3.551	11.72	1.1778	4.05E-04	3.392	myomesin 2 (Myom2)	17930
398	**Gm4691**	6.94	132	1.436	2.71	0.3047	3.15E-06	5.502	predicted gene 4691 (Gm4691)	100043853
399	**Clec11a**	6.93	63	1.984	3.96	0.4542	1.13E-05	4.945	C-type lectin domain family 11, member a (Clec11a)	20256
400	**Ighm**	6.92	119	1.470	2.77	0.3143	3.58E-06	5.446	immunoglobulin heavy constant mu (Ighm)	16019
401	**Prg4**	6.92	241	1.563	2.96	0.3414	4.44E-06	5.353	proteoglycan 4 (megakaryocyte stimulating factor, articular superficial zone protein) (Prg4)	96875
402	**Asb2**	6.90	274	3.054	8.31	0.8747	1.44E-04	3.843	ankyrin repeat and SOCS box-containing 2 (Asb2)	65256
403	**Klhl13**	6.89	129	1.460	2.75	0.3132	3.70E-06	5.432	kelch-like 13 (Klhl13)	67455
404	**Gapdh-ps15**	6.89	442	0.923	1.90	0.1828	1.08E-06	5.968	glyceraldehyde-3-phosphate dehydrogenase, pseudogene 15 (Gapdh-ps15)	100042025
405	**Fbln5**	6.89	497	0.858	1.81	0.1684	9.33E-07	6.030	fibulin 5 (Fbln5)	23876
406	**Krtcap2**	6.88	533	0.821	1.77	0.1602	8.79E-07	6.056	keratinocyte associated protein 2 (Krtcap2)	66059
407	**Pdzrn3**	6.87	724	0.929	1.90	0.1848	1.15E-06	5.939	PDZ domain containing RING finger 3 (Pdzrn3)	55983
408	**Atox1**	6.85	419	1.001	2.00	0.2020	1.43E-06	5.845	antioxidant 1 copper chaperone (Atox1)	11927
409	**Slc2a4**	6.84	134	2.558	5.89	0.6758	5.21E-05	4.283	solute carrier family 2 (facilitated glucose transporter), member 4 (Slc2a4)	20528
410	**Mylk2**	6.81	309	4.084	16.96	1.5066	1.87E-03	2.728	myosin, light polypeptide kinase 2, skeletal muscle (Mylk2)	228785

411	**Ifitm2**	6.79	2204	0.804	1.75	0.1581	1.03E-06	5.989	interferon induced transmembrane protein 2 (Ifitm2)	80876
412	**H2ac15**	6.79	400	0.913	1.88	0.1827	1.32E-06	5.878	H2A clustered histone 15 (H2ac15)	319169
413	**Cped1**	6.76	772	0.878	1.84	0.1748	1.32E-06	5.880	cadherin-like and PC-esterase domain containing 1 (Cped1)	214642
414	**Rps8**	6.76	4533	0.819	1.76	0.1619	1.16E-06	5.937	ribosomal protein S8 (Rps8)	20116
415	**Acacb**	6.74	196	2.215	4.64	0.5541	2.96E-05	4.529	acetyl-Coenzyme A carboxylase beta (Acacb)	100705
416	**Chil3**	6.74	228	1.326	2.51	0.2830	3.82E-06	5.418	chitinase-like 3 (Chil3)	12655
417	**Gm8054**	6.73	57	2.115	4.33	0.5071	2.41E-05	4.618	predicted pseudogene 8054 (Gm8054)	666340
418	**Gm10327**	6.73	116	1.471	2.77	0.3217	5.51E-06	5.259	predicted pseudogene 10327 (Gm10327)	10004139<br>9
419	**Ky**	6.73	83	2.954	7.75	0.8469	1.68E-04	3.774	kyphoscoliosis peptidase (Ky)	16716
420	**Sar1b**	6.71	836	0.806	1.75	0.1594	1.24E-06	5.908	secretion associated Ras related GTPase 1B (Sar1b)	66397
421	**Cse1l**	6.71	2046	0.753	1.68	0.1474	1.10E-06	5.959	chromosome segregation 1 like (Cse1l)	110750
422	**Aip**	6.71	670	0.783	1.72	0.1544	1.18E-06	5.928	aryl-hydrocarbon receptor-interacting protein (Aip)	11632
423	**Fbn1**	6.69	14200	0.900	1.87	0.1811	1.64E-06	5.786	fibrillin 1 (Fbn1)	14118
424	**Cd209g**	6.66	44	2.279	4.85	0.5717	4.18E-05	4.378	CD209g antigen (Cd209g)	70192
425	**Gpx3**	6.61	2956	0.883	1.84	0.1789	1.90E-06	5.722	glutathione peroxidase 3 (Gpx3)	14778
426	**Sgcd**	6.59	103	1.964	3.90	0.4730	2.38E-05	4.623	sarcoglycan, delta (dystrophin-associated glycoprotein) (Sgcd)	24052
427	**Pgm1**	6.54	1231	1.009	2.01	0.2105	2.93E-06	5.534	phosphoglucomutase 1 (Pgm1)	72157
428	**Vtn**	6.54	58	2.190	4.56	0.5536	4.46E-05	4.350	Vitronectin (Vtn)	22370
429	**Nsg1**	6.53	198	1.272	2.42	0.2775	5.53E-06	5.257	neuron specific gene family member 1 (Nsg1)	18196
430	**Plin4**	6.51	735	2.310	4.96	0.6078	6.32E-05	4.199	perilipin 4 (Plin4)	57435
431	**Chchd1**	6.49	322	0.951	1.93	0.1971	2.87E-06	5.542	coiled-coil-helix-coiled-coil-helix domain containing 1 (Chchd1)	66121
432	**Pkia**	6.49	430	1.402	2.64	0.3154	8.25E-06	5.083	protein kinase inhibitor, alpha (Pkia)	18767
433	**Ndufa1**	6.47	278	1.113	2.16	0.2378	4.37E-06	5.360	NADH:ubiquinone oxidoreductase subunit A1 (Ndufa1)	54405
434	**Lifr**	6.47	879	0.775	1.71	0.1561	2.02E-06	5.694	LIF receptor alpha (Lifr)	16880
435	**Tnnc2**	6.45	764	3.907	15.01	1.5404	2.89E-03	2.539	troponin C2, fast (Tnnc2)	21925
436	**Rpl13-ps6**	6.44	302	0.987	1.98	0.2074	3.55E-06	5.449	ribosomal protein L13, pseudogene 6 (Rpl13-ps6)	10004041<br>6

437	**Fam111a**	6.44	2196	0.851	1.80	0.1743	2.60E-06	5.584	family with sequence similarity 111, member A (Fam111a)	107373
438	**Cdk1**	6.42	954	0.751	1.68	0.1514	2.13E-06	5.671	cyclin dependent kinase 1 (Cdk1)	12534
439	**Skic8**	6.40	586	0.849	1.80	0.1745	2.80E-06	5.553	SKI8 subunit of superkiller complex (Skic8)	66317
440	**Hrc**	6.38	209	3.222	9.33	1.0815	6.87E-04	3.163	histidine rich calcium binding protein (Hrc)	15464
441	**Olfml2b**	6.38	1495	0.782	1.72	0.1593	2.55E-06	5.593	olfactomedin-like 2B (Olfml2b)	320078
442	**Gm16374**	6.31	329	0.966	1.95	0.2055	4.58E-06	5.339	predicted pseudogene 16374 (Gm16374)	10003921 4
443	**Fxyd1**	6.29	160	2.031	4.09	0.5275	5.46E-05	4.263	FXYD domain-containing ion transport regulator 1 (Fxyd1)	56188
444	**Creg1**	6.27	567	0.862	1.82	0.1803	3.90E-06	5.409	cellular repressor of E1A-stimulated genes 1 (Creg1)	433375
445	**Ak1**	6.26	833	1.315	2.49	0.3003	1.14E-05	4.943	adenylate kinase 1 (Ak1)	11636
446	**H2ac13**	6.25	232	1.053	2.07	0.2285	6.40E-06	5.194	H2A clustered histone 13 (H2ac13)	319191
447	**Sdhb**	6.24	758	0.823	1.77	0.1715	3.79E-06	5.421	succinate dehydrogenase complex, subunit B, iron sulfur (Ip) (Sdhb)	67680
448	**Shisa2**	6.24	71	2.965	7.81	0.8126	5.26E-04	3.279	shisa family member 2 (Shisa2)	219134
449	**S1pr3**	6.24	406	0.888	1.85	0.1874	4.44E-06	5.352	sphingosine-1-phosphate receptor 3 (S1pr3)	13610
450	**Il11ra1**	6.21	508	1.089	2.13	0.2385	7.55E-06	5.122	interleukin 11 receptor subunit alpha 1 (Il11ra1)	16157
451	**Gpx7**	6.21	118	1.450	2.73	0.3374	1.74E-05	4.759	glutathione peroxidase 7 (Gpx7)	67305
452	**Taf9**	6.19	1025	0.786	1.72	0.1632	3.91E-06	5.407	TATA-box binding protein associated factor 9 (Taf9)	108143
453	**Gm24136**	6.16	249	1.877	3.67	0.4808	5.24E-05	4.281	predicted gene, 24136 (Gm24136)	11548982 1
454	**H1f5**	6.14	640	0.780	1.72	0.1632	4.36E-06	5.361	H1.5 linker histone, cluster member (H1f5)	56702
455	**Gm10481**	6.14	217	1.066	2.09	0.2348	8.43E-06	5.074	predicted gene 10481 (Gm10481)	433845
456	**Podn**	6.13	161	1.720	3.29	0.4271	3.86E-05	4.413	podocin (Podn)	242608
457	**Slc8a3**	6.13	43	3.081	8.46	0.9177	8.99E-04	3.046	solute carrier family 8 (sodium/calcium exchanger), member 3 (Slc8a3)	110893
458	**Fsd2**	6.10	103	2.233	4.70	0.6265	1.35E-04	3.871	fibronectin type III and SPRY domain containing 2 (Fsd2)	244091
459	**Mrps18c**	6.10	219	1.074	2.11	0.2381	9.48E-06	5.023	mitochondrial ribosomal protein S18C (Mrps18c)	68735
460	**Chpt1**	6.10	457	1.042	2.06	0.2295	8.81E-06	5.055	choline phosphotransferase 1 (Chpt1)	212862
461	**Chrdl1**	6.10	747	2.144	4.42	0.5829	1.12E-04	3.951	chordin-like 1 (Chrdl1)	83453
462	**Ms4a4a**	6.08	349	0.989	1.98	0.2162	8.14E-06	5.090	membrane-spanning 4-domains, subfamily A, member 4A (Ms4a4a)	666907

463	**Clic5**	6.07	132	2.097	4.28	0.5715	1.05E-04	3.978	chloride intracellular channel 5 (Clic5)	224796
464	**Sh3bgr**	6.07	77	2.923	7.58	0.8319	7.19E-04	3.143	SH3-binding domain glutamic acid-rich protein (Sh3bgr)	50795
465	**Epdr1**	6.03	313	0.992	1.99	0.2190	9.19E-06	5.037	ependymin related 1 (Epdr1)	105298
466	**Gm13464**	6.03	205	1.131	2.19	0.2560	1.27E-05	4.895	predicted gene 13464 (Gm13464)	10004124<br>5
467	**H3c8**	6.00	508	0.773	1.71	0.1638	5.86E-06	5.232	H3 clustered histone 8 (H3c8)	97908
468	**Hsd11b1**	5.99	160	1.446	2.72	0.3474	2.84E-05	4.546	hydroxysteroid 11-beta dehydrogenase 1 (Hsd11b1)	15483
469	**Abca6**	5.99	70	1.866	3.65	0.4858	7.60E-05	4.119	ATP-binding cassette, sub-family A member 6 (Abca6)	76184
470	**Ptger3**	5.98	95	1.641	3.12	0.4065	4.59E-05	4.339	prostaglandin E receptor 3 (subtype EP3) (Ptger3)	19218
471	**Fgf7**	5.97	366	1.104	2.15	0.2498	1.36E-05	4.865	fibroblast growth factor 7 (Fgf7)	14178
472	**Tspo**	5.97	425	0.870	1.83	0.1885	7.97E-06	5.099	translocator protein (Tspo)	12257
473	**H2bc12**	5.96	272	0.968	1.96	0.2140	1.02E-05	4.993	H2B clustered histone 12 (H2bc12)	319184
474	**Kdelr3**	5.96	366	0.928	1.90	0.2033	9.25E-06	5.034	KDEL (Lys-Asp-Glu-Leu) endoplasmic reticulum protein retention receptor 3 (Kdelr3)	105785
475	**Olfml1**	5.95	55	1.959	3.89	0.5232	1.03E-04	3.989	olfactomedin-like 1 (Olfml1)	244198
476	**Gm22068**	5.95	1262	1.964	3.90	0.5268	1.04E-04	3.983	predicted gene, 22068 (Gm22068)	11548784<br>2
477	**Unc45b**	5.94	159	1.917	3.78	0.5155	9.51E-05	4.022	unc-45 myosin chaperone B (Unc45b)	217012
478	**Selplg**	5.93	544	0.938	1.92	0.2078	1.01E-05	4.994	selectin, platelet (p-selectin) ligand (Selplg)	20345
479	**Smoc2**	5.93	1141	1.172	2.25	0.2707	1.75E-05	4.756	SPARC related modular calcium binding 2 (Smoc2)	64074
480	**Ccn5**	5.92	828	1.122	2.18	0.2566	1.60E-05	4.795	cellular communication network factor 5 (Ccn5)	22403
481	**Uqcr10**	5.88	579	0.938	1.92	0.2092	1.14E-05	4.942	ubiquinol-cytochrome c reductase, complex III subunit X (Uqcr10)	66152
482	**Prnp**	5.87	2143	0.840	1.79	0.1832	9.25E-06	5.034	prion protein (Prnp)	19122
483	**Clec4a3**	5.86	198	1.113	2.16	0.2561	1.79E-05	4.746	C-type lectin domain family 4, member a3 (Clec4a3)	73149
484	**Rora**	5.86	584	1.097	2.14	0.2530	1.74E-05	4.759	RAR-related orphan receptor alpha (Rora)	19883
485	**Mea1**	5.85	508	0.812	1.76	0.1768	9.22E-06	5.035	male enhanced antigen 1 (Mea1)	17256
486	**Rpl10-ps1**	5.83	283	0.939	1.92	0.2103	1.28E-05	4.892	ribosomal protein L10, pseudogene 1 (Rpl10-ps1)	10004339<br>1
487	**Gm4963**	5.83	83	1.619	3.07	0.4122	6.21E-05	4.207	predicted gene 4963 (Gm4963)	243302
488	**Ampd1**	5.82	359	3.168	8.99	1.4038	2.21E-03	2.655	adenosine monophosphate deaminase 1 (Ampd1)	229665
489	**Gm6807**	5.82	356	0.870	1.83	0.1920	1.12E-05	4.952	predicted gene 6807 (Gm6807)	627889

490	**Gm6883**	5.81	49	1.876	3.67	0.5052	1.17E-04	3.932	predicted gene 6883 (Gm6883)	628477
491	**H2ac8**	5.79	234	1.042	2.06	0.2390	1.77E-05	4.752	H2A clustered histone 8 (H2ac8)	319166
492	**Cebpd**	5.78	630	0.906	1.87	0.2025	1.34E-05	4.872	CCAAT/enhancer binding protein delta (Cebpd)	12609
493	**Ecscr**	5.77	269	0.949	1.93	0.2145	1.52E-05	4.817	endothelial cell surface expressed chemotaxis and apoptosis regulator (Ecscr)	68545
494	**Tnxb**	5.74	724	2.749	6.72	0.9984	1.03E-03	2.987	tenascin XB (Tnxb)	81877
495	**Il2rg**	5.73	587	0.879	1.84	0.1968	1.39E-05	4.855	interleukin 2 receptor, gamma chain (Il2rg)	16186
496	**Tfpi**	5.73	312	1.090	2.13	0.2539	2.29E-05	4.641	tissue factor pathway inhibitor (Tfpi)	21788
497	**Npl**	5.73	215	1.111	2.16	0.2597	2.40E-05	4.619	N-acetylneuraminate pyruvate lyase (Npl)	74091
498	**Clec4a1**	5.69	249	1.073	2.10	0.2516	2.39E-05	4.622	C-type lectin domain family 4, member a1 (Clec4a1)	269799
499	**Tspan11**	5.68	387	0.862	1.82	0.1930	1.52E-05	4.818	tetraspanin 11 (Tspan11)	68498
500	**Entpd2**	5.62	116	1.506	2.84	0.3911	7.70E-05	4.113	ectonucleoside triphosphate diphosphohydrolase 2 (Entpd2)	12496
501	**Prkcq**	5.62	102	1.616	3.07	0.4232	9.99E-05	4.001	protein kinase C, theta (Prkcq)	18761
502	**C1galt1c1**	5.61	421	0.869	1.83	0.1968	1.83E-05	4.737	C1GALT1-specific chaperone 1 (C1galt1c1)	59048
503	**Synpo2l**	5.59	90	2.102	4.29	0.6227	3.22E-04	3.492	synaptopodin 2-like (Synpo2l)	68760
504	**Tpsb2**	5.57	67	1.875	3.67	0.5342	2.01E-04	3.696	tryptase beta 2 (Tpsb2)	17229
505	**P2ry12**	5.55	200	1.297	2.46	0.3239	5.61E-05	4.251	purinergic receptor P2Y, G-protein coupled 12 (P2ry12)	70839
506	**Ly9**	5.54	167	1.123	2.18	0.2707	3.83E-05	4.417	lymphocyte antigen 9 (Ly9)	17085
507	**Tmeff2**	5.52	52	2.022	4.06	0.5988	3.15E-04	3.501	transmembrane protein with EGF-like and two follistatin-like domains 2 (Tmeff2)	56363
508	**H2bc7**	5.52	190	1.055	2.08	0.2517	3.41E-05	4.467	H2B clustered histone 7 (H2bc7)	319180
509	**Col6a5**	5.51	77	1.563	2.95	0.4162	1.12E-04	3.952	collagen, type VI, alpha 5 (Col6a5)	665033
510	**Emilin1**	5.51	736	0.769	1.70	0.1725	1.81E-05	4.742	elastin microfibril interfacer 1 (Emilin1)	100952
511	**Clec2d**	5.48	1004	0.964	1.95	0.2264	3.02E-05	4.520	C-type lectin domain family 2, member d (Clec2d)	93694
512	**Ndufv3**	5.48	565	0.819	1.76	0.1870	2.18E-05	4.662	NADH:ubiquinone oxidoreductase core subunit V3 (Ndufv3)	78330
513	**Cd248**	5.48	1333	0.922	1.90	0.2151	2.77E-05	4.557	CD248 antigen, endosialin (Cd248)	70445
514	**Vldlr**	5.47	490	1.148	2.22	0.2814	4.72E-05	4.326	very low density lipoprotein receptor (Vldlr)	22359

515	**Gm22042**	5.46	178	1.922	3.79	0.5707	2.88E-04	3.541	predicted gene, 22042 (Gm22042)	115490033
516	**Tfpi2**	5.44	230	1.352	2.55	0.3479	8.11E-05	4.091	tissue factor pathway inhibitor 2 (Tfpi2)	21789
517	**H3c10**	5.40	428	0.835	1.78	0.1934	2.70E-05	4.569	H3 clustered histone 10 (H3c10)	319152
518	**Trim72**	5.39	124	2.075	4.21	0.6414	4.79E-04	3.319	tripartite motif-containing 72 (Trim72)	434246
519	**Aldh1a1**	5.38	106	2.131	4.38	0.7022	5.65E-04	3.248	aldehyde dehydrogenase family 1, subfamily A1 (Aldh1a1)	11668
520	**Dgat2**	5.38	67	1.574	2.98	0.4306	1.58E-04	3.802	diacylglycerol O-acyltransferase 2 (Dgat2)	67800
521	**Gm11953**	5.37	47	1.796	3.47	0.5165	2.64E-04	3.578	predicted gene 11953 (Gm11953)	100041720
522	**Des**	5.37	1786	2.292	4.90	0.7765	8.28E-04	3.082	Desmin (Des)	13346
523	**Coq8a**	5.36	304	1.295	2.45	0.3346	8.60E-05	4.066	coenzyme Q8A (Coq8a)	67426
524	**Cd300ld3**	5.36	104	1.334	2.52	0.3468	9.50E-05	4.022	CD300 molecule like family member D3 (Cd300ld3)	382551
525	**Emp3**	5.34	1138	0.800	1.74	0.1849	2.88E-05	4.541	epithelial membrane protein 3 (Emp3)	13732
526	**Cyb5a**	5.34	723	0.753	1.69	0.1729	2.61E-05	4.584	cytochrome b5 type A (microsomal) (Cyb5a)	109672
527	**Rpl5-ps2**	5.34	198	1.020	2.03	0.2485	4.84E-05	4.315	ribisomal protein L5, pseudogene 2 (Rpl5-ps2)	668936
528	**Cd300ld5**	5.33	93	1.395	2.63	0.3688	1.15E-04	3.940	CD300 molecule like family member D5 (Cd300ld5)	100043125
529	**H2-Eb1**	5.33	1489	0.859	1.81	0.2026	3.37E-05	4.472	histocompatibility 2, class II antigen E beta (H2-Eb1)	14969
530	**Echdc1**	5.33	313	0.902	1.87	0.2140	3.76E-05	4.425	enoyl Coenzyme A hydratase domain containing 1 (Echdc1)	52665
531	**Mrps27**	5.32	364	0.825	1.77	0.1927	3.21E-05	4.493	mitochondrial ribosomal protein S27 (Mrps27)	218506
532	**Slc25a12**	5.31	687	0.984	1.98	0.2392	4.75E-05	4.324	solute carrier family 25 (mitochondrial carrier, Aralar), member 12 (Slc25a12)	78830
533	**P4ha3**	5.29	326	0.837	1.79	0.1969	3.55E-05	4.450	procollagen-proline, 2-oxoglutarate 4-dioxygenase (proline 4-hydroxylase), alpha polypeptide III (P4ha3)	320452
534	**Gm2308**	5.28	108	1.317	2.49	0.3463	1.09E-04	3.964	predicted gene 2308 (Gm2308)	100039556
535	**Fibin**	5.28	832	0.903	1.87	0.2158	4.20E-05	4.376	fin bud initiation factor homolog (zebrafish) (Fibin)	67606
536	**Mrps22**	5.27	303	0.939	1.92	0.2266	4.68E-05	4.329	mitochondrial ribosomal protein S22 (Mrps22)	64655
537	**Synpo2**	5.25	413	1.396	2.63	0.3812	1.41E-04	3.850	synaptopodin 2 (Synpo2)	118449
538	**Tac1**	5.24	97	2.046	4.13	0.6569	6.36E-04	3.197	tachykinin 1 (Tac1)	21333
539	**Gm3534**	5.23	179	1.081	2.11	0.2712	7.06E-05	4.151	predicted pseudogene 3534 (Gm3534)	100041831

540	**Rpl10-ps5**	5.22	260	0.902	1.87	0.2173	4.76E-05	4.322	ribosomal protein L10, pseudogene 5 (Rpl10-ps5)	100041555
541	**Mt2**	5.22	733	1.072	2.10	0.2714	7.19E-05	4.144	metallothionein 2 (Mt2)	17750
542	**Atp5md**	5.21	1035	0.821	1.77	0.1942	4.10E-05	4.388	#N/A	#N/A
543	**Rpl31-ps14**	5.19	92	1.335	2.52	0.3579	1.39E-04	3.858	ribosomal protein L31, pseudogene 14 (Rpl31-ps14)	100043192
544	**Ano5**	5.18	306	2.269	4.82	0.8084	1.22E-03	2.914	anoctamin 5 (Ano5)	233246
545	**H2ac10**	5.18	242	0.933	1.91	0.2282	5.63E-05	4.250	H2A clustered histone 10 (H2ac10)	319173
546	**Bcat1**	5.18	299	0.871	1.83	0.2094	4.93E-05	4.307	branched chain aminotransferase 1, cytosolic (Bcat1)	12035
547	**Dram1**	5.17	522	0.771	1.71	0.1810	3.98E-05	4.400	DNA-damage regulated autophagy modulator 1 (Dram1)	71712
548	**Naa38**	5.15	322	0.878	1.84	0.2128	5.31E-05	4.275	N(alpha)-acetyltransferase 38, NatC auxiliary subunit (Naa38)	78304
549	**Tsku**	5.13	549	0.810	1.75	0.1932	4.80E-05	4.319	tsukushi, small leucine rich proteoglycan (Tsku)	244152
550	**Gapdh-ps16**	5.10	335	0.867	1.82	0.2112	5.79E-05	4.238	glyceraldehyde-3-phosphate dehydrogenase, pseudogene 16 (Gapdh-ps16)	100041325
551	**Fbln1**	5.10	759	1.020	2.03	0.2578	8.24E-05	4.084	fibulin 1 (Fbln1)	14114
552	**Gm11353**	5.10	148	1.105	2.15	0.2853	1.00E-04	3.999	predicted gene 11353 (Gm11353)	100042180
553	**Gm3200**	5.10	117	1.213	2.32	0.3214	1.30E-04	3.887	predicted pseudogene 3200 (Gm3200)	100041204
554	**Heph**	5.10	56	1.605	3.04	0.4679	3.23E-04	3.491	Hephaestin (Heph)	15203
555	**Dynlt1b**	5.08	393	0.761	1.69	0.1808	4.80E-05	4.319	dynein light chain Tctex-type 1B (Dynlt1b)	21648
556	**Scn4b**	5.08	194	2.581	5.98	1.1548	3.20E-03	2.494	sodium channel, type IV, beta (Scn4b)	399548
557	**Mlip**	5.06	126	2.008	4.02	0.6865	8.98E-04	3.047	muscular LMNA-interacting protein (Mlip)	69642
558	**Itga11**	5.05	148	1.232	2.35	0.3302	1.51E-04	3.822	integrin alpha 11 (Itga11)	319480
559	**Gstm1**	5.05	277	1.153	2.22	0.3058	1.26E-04	3.900	glutathione S-transferase, mu 1 (Gstm1)	14862
560	**Hmcn2**	5.05	246	2.698	6.49	1.3964	4.48E-03	2.349	hemicentin 2 (Hmcn2)	665700
561	**Myh11**	5.05	208	1.179	2.26	0.3149	1.36E-04	3.867	myosin, heavy polypeptide 11, smooth muscle (Myh11)	17880
562	**Mmp10**	5.04	154	1.426	2.69	0.4033	2.45E-04	3.612	matrix metallopeptidase 10 (Mmp10)	17384
563	**Serpinb1a**	5.03	231	1.058	2.08	0.2739	1.06E-04	3.977	serine (or cysteine) peptidase inhibitor, clade B, member 1a (Serpinb1a)	66222
564	**Pirb**	5.03	715	0.782	1.72	0.1890	5.68E-05	4.246	paired Ig-like receptor B (Pirb)	18733

565	**Ccl12**	5.01	104	1.353	2.55	0.3758	2.20E-04	3.658	C-C motif chemokine ligand 12 (Ccl12)	20293
566	**Ccl17**	5.00	49	1.830	3.55	0.5804	6.81E-04	3.167	C-C motif chemokine ligand 17 (Ccl17)	20295
567	**Gm14130**	4.98	51	1.712	3.28	0.5291	5.36E-04	3.271	predicted gene 14130 (Gm14130)	667048
568	**Krt27**	4.98	86	1.678	3.20	0.5224	4.98E-04	3.303	keratin 27 (Krt27)	16675
569	**Lama2**	4.97	711	0.952	1.93	0.2437	9.66E-05	4.015	laminin, alpha 2 (Lama2)	16773
570	**Tmem119**	4.95	640	0.981	1.97	0.2519	1.08E-04	3.968	transmembrane protein 119 (Tmem119)	231633
571	**Gfpt2**	4.92	565	0.906	1.87	0.2286	9.63E-05	4.016	glutamine fructose-6-phosphate transaminase 2 (Gfpt2)	14584
572	**H2ac22**	4.91	222	0.919	1.89	0.2337	1.01E-04	3.995	H2A clustered histone 22 (H2ac22)	319170
573	**Ciao2b**	4.91	312	0.814	1.76	0.2012	7.99E-05	4.097	cytosolic iron-sulfur assembly component 2B (Ciao2b)	68523
574	**Snrpf**	4.89	587	0.791	1.73	0.1955	8.03E-05	4.095	small nuclear ribonucleoprotein polypeptide F (Snrpf)	69878
575	**Asb15**	4.88	94	2.934	7.64	1.6995	1.13E-02	1.947	ankyrin repeat and SOCS box-containing 15 (Asb15)	78910
576	**Atp5mpl**	4.88	290	0.885	1.85	0.2244	1.01E-04	3.994	No gene name found in the DAVID Knowledgebase	0
577	**Myom1**	4.86	854	2.419	5.35	1.1222	3.61E-03	2.442	myomesin 1 (Myom1)	17929
578	**Trarg1**	4.82	61	1.494	2.82	0.4514	4.73E-04	3.325	trafficking regulator of GLUT4 (SLC2A4) 1 (Trarg1)	237858
579	**Gm4841**	4.81	43	1.838	3.57	0.6008	1.07E-03	2.971	predicted gene 4841 (Gm4841)	225594
580	**Ppp1r3c**	4.80	254	2.582	5.99	1.5105	6.01E-03	2.221	protein phosphatase 1, regulatory subunit 3C (Ppp1r3c)	53412
581	**Nqo1**	4.80	43	1.794	3.47	0.6006	9.85E-04	3.007	NAD(P)H dehydrogenase, quinone 1 (Nqo1)	18104
582	**Ptcd2**	4.80	347	0.873	1.83	0.2233	1.19E-04	3.923	pentatricopeptide repeat domain 2 (Ptcd2)	68927
583	**Ndufc1**	4.76	249	0.887	1.85	0.2296	1.33E-04	3.875	NADH:ubiquinone oxidoreductase subunit C1 (Ndufc1)	66377
584	**Ptx3**	4.76	203	1.528	2.88	0.4808	5.83E-04	3.234	pentraxin related gene (Ptx3)	19288
585	**Csrp2**	4.76	922	0.873	1.83	0.2246	1.30E-04	3.888	cysteine and glycine-rich protein 2 (Csrp2)	13008
586	**Cd300ld4**	4.76	78	1.367	2.58	0.4027	4.04E-04	3.393	CD300 molecule like family member D4 (Cd300ld4)	10004312<br>3
587	**Gm2546**	4.75	202	0.951	1.93	0.2511	1.58E-04	3.802	predicted gene 2546 (Gm2546)	10004000<br>1
588	**Crabp1**	4.75	1882	0.865	1.82	0.2224	1.30E-04	3.888	cellular retinoic acid binding protein I (Crabp1)	12903
589	**Vegfd**	4.75	201	0.981	1.97	0.2608	1.70E-04	3.769	vascular endothelial growth factor D (Vegfd)	14205
590	**Rnu1a1**	4.75	979	1.649	3.14	0.5341	7.98E-04	3.098	U1a1 small nuclear RNA (Rnu1a1)	19842
591	**Chrna1**	4.74	83	1.555	2.94	0.4794	6.48E-04	3.188	cholinergic receptor nicotinic alpha 1 subunit (Chrna1)	11435
592	**Ssc5d**	4.74	314	0.820	1.77	0.2085	1.19E-04	3.923	scavenger receptor cysteine rich family, 5 domains (Ssc5d)	269855

593	**Gm6394**	4.74	87	1.317	2.49	0.3860	3.80E-04	3.420	predicted gene 6394 (Gm6394)	623114
594	**Gabra3**	4.73	44	1.719	3.29	0.5699	9.64E-04	3.016	gamma-aminobutyric acid type A receptor subunit alpha 3 (Gabra3)	14396
595	**Gm4654**	4.73	143	1.061	2.09	0.2896	2.13E-04	3.672	predicted gene 4654 (Gm4654)	100043797
596	**Gm15501**	4.73	197	0.989	1.98	0.2652	1.83E-04	3.738	predicted pseudogene 15501 (Gm15501)	100040298
597	**Abca9**	4.73	807	0.779	1.72	0.1972	1.13E-04	3.947	ATP-binding cassette, sub-family A member 9 (Abca9)	217262
598	**Slc16a3**	4.72	487	0.771	1.71	0.1940	1.12E-04	3.952	solute carrier family 16 (monocarboxylic acid transporters), member 3 (Slc16a3)	80879
599	**Nox4**	4.72	194	1.010	2.01	0.2731	1.94E-04	3.712	NADPH oxidase 4 (Nox4)	50490
600	**Layn**	4.72	115	1.455	2.74	0.4432	5.42E-04	3.266	Layilin (Layn)	244864
601	**Ms4a6b**	4.71	655	0.768	1.70	0.1932	1.14E-04	3.944	membrane-spanning 4-domains, subfamily A, member 6B (Ms4a6b)	69774
602	**Klhl41**	4.70	276	2.419	5.35	1.2005	5.19E-03	2.285	kelch-like 41 (Klhl41)	228003
603	**Smyd1**	4.69	169	2.505	5.68	1.4521	6.50E-03	2.187	SET and MYND domain containing 1 (Smyd1)	12180
604	**Fzd4**	4.69	582	0.812	1.76	0.2081	1.33E-04	3.877	frizzled class receptor 4 (Fzd4)	14366
605	**Steap1**	4.67	349	0.758	1.69	0.1916	1.22E-04	3.914	six transmembrane epithelial antigen of the prostate 1 (Steap1)	70358
606	**A4galt**	4.66	213	0.946	1.93	0.2531	1.92E-04	3.716	alpha 1,4-galactosyltransferase (A4galt)	239559
607	**Abcd2**	4.66	196	1.112	2.16	0.3143	2.84E-04	3.547	ATP-binding cassette, sub-family D member 2 (Abcd2)	26874
608	**Angptl1**	4.65	269	0.894	1.86	0.2365	1.75E-04	3.758	angiopoietin-like 1 (Angptl1)	72713
609	**H2bc6**	4.64	288	0.838	1.79	0.2182	1.59E-04	3.798	H2B clustered histone 6 (H2bc6)	319179
610	**Slc28a2**	4.61	60	1.556	2.94	0.5102	8.89E-04	3.051	solute carrier family 28 (sodium-coupled nucleoside transporter), member 2 (Slc28a2)	269346
611	**Hagh**	4.59	264	0.841	1.79	0.2207	1.77E-04	3.752	hydroxyacyl glutathione hydrolase (Hagh)	14651
612	**Prkag3**	4.59	43	2.502	5.66	1.0969	8.16E-03	2.088	protein kinase, AMP-activated, gamma 3 non-catalytic subunit (Prkag3)	241113
613	**Pcdh20**	4.58	45	1.620	3.07	0.5384	1.08E-03	2.965	protocadherin 20 (Pcdh20)	219257
614	**Sell**	4.58	242	1.169	2.25	0.3377	3.89E-04	3.410	selectin, lymphocyte (Sell)	20343
615	**Nop10**	4.57	332	0.832	1.78	0.2191	1.82E-04	3.740	NOP10 ribonucleoprotein (Nop10)	66181

616	**Ces2g**	4.56	61	1.654	3.15	0.5732	1.25E-03	2.905	carboxylesterase 2G (Ces2g)	72361
617	**Pknox2**	4.55	128	1.522	2.87	0.5002	9.33E-04	3.030	Pbx/knotted 1 homeobox 2 (Pknox2)	208076
618	**H2ac4**	4.54	463	0.783	1.72	0.2047	1.75E-04	3.756	H2A clustered histone 4 (H2ac4)	319172
619	**Ms4a6c**	4.50	680	0.923	1.90	0.2525	2.64E-04	3.578	membrane-spanning 4-domains, subfamily A, member 6C (Ms4a6c)	73656
620	**Mthfd2**	4.50	350	0.757	1.69	0.1971	1.81E-04	3.742	methylenetetrahydrofolate dehydrogenase (NAD+ dependent), methenyltetrahydrofolate cyclohydrolase (Mthfd2)	17768
621	**Plpp7**	4.50	42	1.995	3.98	0.8226	3.14E-03	2.503	phospholipid phosphatase 7 (inactive) (Plpp7)	227721
622	**Prkar2b**	4.50	615	0.852	1.81	0.2285	2.27E-04	3.645	protein kinase, cAMP dependent regulatory, type II beta (Prkar2b)	19088
623	**Rgs7bp**	4.50	49	1.553	2.93	0.5187	1.14E-03	2.944	regulator of G-protein signalling 7 binding protein (Rgs7bp)	52882
624	**Aldh6a1**	4.49	401	0.837	1.79	0.2240	2.20E-04	3.657	aldehyde dehydrogenase family 6, subfamily A1 (Aldh6a1)	104776
625	**Mfsd4a**	4.48	811	1.905	3.74	0.7438	2.68E-03	2.573	major facilitator superfamily domain containing 4A (Mfsd4a)	213006
626	**Nxpe5**	4.47	248	1.044	2.06	0.3005	3.77E-04	3.424	neurexophilin and PC-esterase domain family, member 5 (Nxpe5)	381680
627	**Stard8**	4.46	397	0.833	1.78	0.2245	2.36E-04	3.628	StAR related lipid transfer domain containing 8 (Stard8)	236920
628	**Ndufa11**	4.46	340	0.840	1.79	0.2268	2.40E-04	3.620	NADH:ubiquinone oxidoreductase subunit A11 (Ndufa11)	69875
629	**Mrpl22**	4.46	344	0.818	1.76	0.2191	2.29E-04	3.640	mitochondrial ribosomal protein L22 (Mrpl22)	216767
630	**S100a8**	4.45	171	1.501	2.83	0.5004	1.11E-03	2.953	S100 calcium binding protein A8 (calgranulin A)(S100a8)	20201
631	**Zfp984**	4.45	395	0.808	1.75	0.2154	2.27E-04	3.645	zinc finger protein 984 (Zfp984)	10004167<br>7
632	**S1pr1**	4.45	557	0.932	1.91	0.2585	3.03E-04	3.518	sphingosine-1-phosphate receptor 1 (S1pr1)	13609
633	**S100a9**	4.43	139	1.598	3.03	0.5549	1.45E-03	2.837	S100 calcium binding protein A9 (calgranulin B) (S100a9)	20202
634	**Scn3a**	4.42	57	1.515	2.86	0.5164	1.24E-03	2.906	sodium channel, voltage-gated, type III, alpha (Scn3a)	20269
635	**Abcb1a**	4.42	402	0.786	1.72	0.2091	2.34E-04	3.630	ATP-binding cassette, sub-family B member 1A (Abcb1a)	18671
636	**Gm5619**	4.39	234	0.838	1.79	0.2284	2.81E-04	3.551	predicted gene 5619 (Gm5619)	434426
637	**Sspn**	4.38	355	0.933	1.91	0.2618	3.53E-04	3.452	Sarcospan (Sspn)	16651
638	**Eln**	4.38	472	1.153	2.22	0.3517	5.90E-04	3.229	Elastin (Eln)	13717
639	**Aldh1l1**	4.38	118	1.121	2.17	0.3364	5.50E-04	3.259	aldehyde dehydrogenase 1 family, member L1 (Aldh1l1)	107747

640	**Dpy30**	4.37	315	0.754	1.69	0.2007	2.43E-04	3.615	dpy-30, histone methyltransferase complex regulatory subunit (Dpy30)	66310
641	**Apoo**	4.36	140	1.011	2.01	0.2926	4.45E-04	3.351	apolipoprotein O (Apoo)	68316
642	**Aldh1l2**	4.35	236	1.029	2.04	0.3002	4.82E-04	3.317	aldehyde dehydrogenase 1 family, member L2 (Aldh1l2)	216188
643	**Apobec2**	4.33	102	2.321	5.00	1.7484	9.82E-03	2.008	apolipoprotein B mRNA editing enzyme, catalytic polypeptide 2 (Apobec2)	11811
644	**Cpz**	4.31	69	1.385	2.61	0.4615	1.18E-03	2.928	carboxypeptidase Z (Cpz)	242939
645	**Slc11a1**	4.31	312	0.893	1.86	0.2510	3.81E-04	3.419	solute carrier family 11 (proton-coupled divalent metal ion transporters), member 1 (Slc11a1)	18173
646	**Rnu1b2**	4.31	778	1.981	3.95	0.8951	4.68E-03	2.330	U1b2 small nuclear RNA (Rnu1b2)	19845
647	**Tubb3**	4.29	162	0.987	1.98	0.2891	5.00E-04	3.301	tubulin, beta 3 class III (Tubb3)	22152
648	**Il16**	4.28	262	0.832	1.78	0.2315	3.54E-04	3.451	interleukin 16 (Il16)	16170
649	**Tnfaip6**	4.28	132	1.115	2.17	0.3425	6.82E-04	3.166	tumor necrosis factor alpha induced protein 6 (Tnfaip6)	21930
650	**Pcdh18**	4.27	774	0.866	1.82	0.2434	3.95E-04	3.403	protocadherin 18 (Pcdh18)	73173
651	**Cd59a**	4.26	77	1.279	2.43	0.4176	1.05E-03	2.979	CD59a antigen (Cd59a)	12509
652	**Adamts20**	4.25	28	2.240	4.72	0.9945	9.73E-03	2.012	ADAM metallopeptidase with thrombospondin type 1 motif 20 (Adamts20)	223838
653	**Clec10a**	4.23	139	1.421	2.68	0.5051	1.53E-03	2.814	C-type lectin domain family 10, member A (Clec10a)	17312
654	**Prelp**	4.23	571	1.599	3.03	0.6071	2.33E-03	2.632	proline arginine-rich end leucine-rich repeat (Prelp)	116847
655	**Gria3**	4.23	180	0.923	1.90	0.2673	4.97E-04	3.304	glutamate receptor, ionotropic, AMPA3 (alpha 3) (Gria3)	53623
656	**Mef2c**	4.23	1196	0.936	1.91	0.2749	5.14E-04	3.289	myocyte enhancer factor 2C (Mef2c)	17260
657	**Clec4a2**	4.22	185	0.919	1.89	0.2656	5.01E-04	3.300	C-type lectin domain family 4, member a2 (Clec4a2)	26888
658	**Prrg3**	4.20	108	1.134	2.19	0.3570	8.62E-04	3.065	proline rich Gla (G-carboxyglutamic acid) 3 (transmembrane) (Prrg3)	208748
659	**Myoz3**	4.19	65	1.868	3.65	0.8698	4.71E-03	2.327	myozenin 3 (Myoz3)	170947
660	**Kbtbd12**	4.19	44	2.269	4.82	1.0765	1.19E-02	1.924	kelch repeat and BTB (POZ) domain containing 12 (Kbtbd12)	74589
661	**Rpl31-ps16**	4.19	97	1.134	2.19	0.3562	8.79E-04	3.056	ribosomal protein L31, pseudogene 16 (Rpl31-ps16)	668850
662	**Naalad2**	4.17	194	0.946	1.93	0.2787	6.00E-04	3.222	N-acetylated alpha-linked acidic dipeptidase 2 (Naalad2)	72560
663	**Mrps36**	4.15	217	0.825	1.77	0.2343	4.71E-04	3.327	mitochondrial ribosomal protein S36 (Mrps36)	66128

664	**Adm**	4.15	87	1.347	2.54	0.4690	1.57E-03	2.804	Adrenomedullin (Adm)	11535
665	**C1qtnf2**	4.15	58	1.438	2.71	0.5118	1.95E-03	2.709	C1q and tumor necrosis factor related protein 2 (C1qtnf2)	69183
666	**Zfp268**	4.13	220	0.830	1.78	0.2375	4.97E-04	3.304	zinc finger protein 268 (Zfp268)	433801
667	**Ear2**	4.13	83	1.317	2.49	0.4556	1.53E-03	2.815	eosinophil-associated, ribonuclease A family, member 2 (Ear2)	13587
668	**Cd37**	4.12	129	1.092	2.13	0.3454	9.33E-04	3.030	CD37 antigen (Cd37)	12493
669	**Cdo1**	4.11	106	1.099	2.14	0.3478	9.68E-04	3.014	cysteine dioxygenase 1, cytosolic (Cdo1)	12583
670	**Zfp521**	4.09	424	0.851	1.80	0.2481	5.73E-04	3.242	zinc finger protein 521 (Zfp521)	225207
671	**Cyp2j6**	4.08	172	0.985	1.98	0.3015	7.95E-04	3.100	cytochrome P450, family 2, subfamily j, polypeptide 6 (Cyp2j6)	13110
672	**Adamts15**	4.07	323	1.040	2.06	0.3256	9.33E-04	3.030	ADAM metallopeptidase with thrombospondin type 1 motif 15 (Adamts15)	235130
673	**Armh4**	4.05	32	1.614	3.06	0.6463	3.65E-03	2.437	armadillo-like helical domain containing 4 (Armh4)	67419
674	**Boc**	4.04	223	0.830	1.78	0.2420	6.18E-04	3.209	BOC cell adhesion associated, oncogene regulated (Boc)	117606
675	**B3gnt9**	4.02	260	1.059	2.08	0.3383	1.08E-03	2.965	UDP-GlcNAc:betaGal beta-1,3-N-acetylglucosaminyltransferase 9 (B3gnt9)	97440
676	**Ndrg2**	4.02	747	1.753	3.37	0.8199	5.41E-03	2.267	N-myc downstream regulated gene 2 (Ndrg2)	29811
677	**Pf4**	4.02	332	0.800	1.74	0.2315	6.05E-04	3.219	platelet factor 4 (Pf4)	56744
678	**Mcoln2**	4.01	82	1.290	2.45	0.4530	1.92E-03	2.717	mucolipin 2 (Mcoln2)	68279
679	**Lrrn4cl**	4.01	331	0.797	1.74	0.2309	6.18E-04	3.209	LRRN4 C-terminal like (Lrrn4cl)	68852
680	**Tnfrsf11b**	4.00	344	1.041	2.06	0.3326	1.09E-03	2.963	tumor necrosis factor receptor superfamily, member 11b (osteoprotegerin) (Tnfrsf11b)	18383
681	**Gm4217**	3.99	84	1.275	2.42	0.4476	1.91E-03	2.719	predicted gene 4217 (Gm4217)	10004308<br>4
682	**Gm4815**	3.99	120	1.016	2.02	0.3217	1.07E-03	2.972	predicted gene 4815 (Gm4815)	218501
683	**Cxcl13**	3.97	39	2.019	4.05	1.2711	1.11E-02	1.953	C-X-C motif chemokine ligand 13 (Cxcl13)	55985
684	**Nova1**	3.96	224	1.065	2.09	0.3474	1.28E-03	2.893	NOVA alternative splicing regulator 1 (Nova1)	664883
685	**Fbxo32**	3.93	1274	0.775	1.71	0.2279	6.93E-04	3.159	F-box protein 32 (Fbxo32)	67731
686	**Gm26397**	3.93	234	0.821	1.77	0.2440	7.71E-04	3.113	predicted gene, 26397 (Gm26397)	11548704<br>3
687	**Lrrc2**	3.92	53	1.610	3.05	0.6719	4.87E-03	2.312	leucine rich repeat containing 2 (Lrrc2)	74249

688	**Ccl7**	3.92	283	0.974	1.96	0.3084	1.14E-03	2.944	C-C motif chemokine ligand 7 (Ccl7)	20306
689	**Styxl2**	3.91	40	1.762	3.39	0.8070	7.13E-03	2.147	serine/threonine/tyrosine interacting like 2 (Styxl2)	240892
690	**Kmo**	3.90	91	1.161	2.24	0.3990	1.83E-03	2.737	kynurenine 3-monooxygenase (Kmo)	98256
691	**Aoah**	3.90	272	0.800	1.74	0.2372	8.02E-04	3.096	acyloxyacyl hydrolase (Aoah)	27052
692	**Colec12**	3.89	1242	1.314	2.49	0.4850	2.63E-03	2.580	collectin sub-family member 12 (Colec12)	140792
693	**Txlnb**	3.89	206	1.845	3.59	1.0569	9.08E-03	2.042	taxilin beta (Txlnb)	378431
694	**Glipr1**	3.86	292	1.008	2.01	0.3297	1.40E-03	2.854	GLI pathogenesis related 1 (Glipr1)	73690
695	**Ndn**	3.86	81	1.147	2.21	0.3975	1.94E-03	2.713	necdin, MAGE family member (Ndn)	17984
696	**Mmp23**	3.85	127	0.961	1.95	0.3103	1.30E-03	2.885	matrix metallopeptidase 23 (Mmp23)	26561
697	**Dio2**	3.84	343	0.939	1.92	0.3000	1.25E-03	2.903	deiodinase, iodothyronine, type II (Dio2)	13371
698	**Wif1**	3.82	79	1.236	2.36	0.4491	2.63E-03	2.581	Wnt inhibitory factor 1 (Wif1)	24117
699	**Lsm5**	3.80	148	0.894	1.86	0.2841	1.24E-03	2.907	LSM5 homolog, U6 small nuclear RNA and mRNA degradation associated (Lsm5)	66373
700	**Ripk3**	3.80	174	0.850	1.80	0.2647	1.13E-03	2.947	receptor-interacting serine-threonine kinase 3 (Ripk3)	56532
701	**Pi15**	3.80	985	0.772	1.71	0.2317	9.48E-04	3.023	peptidase inhibitor 15 (Pi15)	94227
702	**Cdh10**	3.79	103	1.266	2.41	0.4744	2.97E-03	2.527	cadherin 10 (Cdh10)	320873
703	**Mgl2**	3.79	217	0.833	1.78	0.2583	1.10E-03	2.960	macrophage galactose N-acetyl-galactosamine specific lectin 2 (Mgl2)	216864
704	**Mcpt2**	3.76	48	1.478	2.79	0.6385	5.27E-03	2.278	mast cell protease 2 (Mcpt2)	17225
705	**Pira2**	3.75	130	0.947	1.93	0.3110	1.57E-03	2.803	paired-Ig-like receptor A2 (Pira2)	18725
706	**Sox6**	3.75	187	1.842	3.59	1.3644	1.24E-02	1.907	SRY (sex determining region Y)-box 6 (Sox6)	20679
707	**Esm1**	3.74	364	1.097	2.14	0.3896	2.29E-03	2.640	endothelial cell-specific molecule 1 (Esm1)	71690
708	**Stmn2**	3.73	537	0.925	1.90	0.3032	1.57E-03	2.803	stathmin-like 2 (Stmn2)	20257
709	**Scn1b**	3.72	275	1.152	2.22	0.4285	2.70E-03	2.569	sodium channel, voltage-gated, type I, beta (Scn1b)	20266
710	**Ptgir**	3.72	63	1.221	2.33	0.4619	3.19E-03	2.497	prostaglandin I receptor (IP) (Ptgir)	19222
711	**Gm12286**	3.72	175	0.898	1.86	0.2917	1.53E-03	2.817	predicted gene 12286 (Gm12286)	10004279<br>9
712	**Cavin2**	3.71	374	0.766	1.70	0.2364	1.14E-03	2.942	caveolae associated 2 (Cavin2)	20324
713	**Mcpt1**	3.70	66	1.316	2.49	0.5267	4.08E-03	2.389	mast cell protease 1 (Mcpt1)	17224
714	**Mylip**	3.69	313	0.755	1.69	0.2314	1.15E-03	2.939	myosin regulatory light chain interacting protein (Mylip)	218203

715	**Ptafr**	3.69	314	0.796	1.74	0.2486	1.28E-03	2.894	platelet-activating factor receptor (Ptafr)	19204
716	**Homer2**	3.67	35	1.563	2.95	0.7156	7.74E-03	2.111	homer scaffolding protein 2 (Homer2)	26557
717	**Casp1**	3.67	451	0.837	1.79	0.2674	1.46E-03	2.836	caspase 1 (Casp1)	12362
718	**Filip1**	3.67	192	1.054	2.08	0.3765	2.45E-03	2.611	filamin A interacting protein 1 (Filip1)	70598
719	**Rgs18**	3.66	49	1.333	2.52	0.5524	4.77E-03	2.322	regulator of G-protein signaling 18 (Rgs18)	64214
720	**Prkaa2**	3.65	349	1.657	3.15	0.9565	1.02E-02	1.991	protein kinase, AMP-activated, alpha 2 catalytic subunit (Prkaa2)	108079
721	**Gm16470**	3.65	73	1.121	2.17	0.4127	2.97E-03	2.527	predicted pseudogene 16470 (Gm16470)	676923
722	**Rps19-ps6**	3.65	72	1.140	2.20	0.4237	3.12E-03	2.506	ribosomal protein S19, pseudogene 6 (Rps19-ps6)	100503302
723	**Ugt1a6a**	3.64	179	0.986	1.98	0.3388	2.20E-03	2.657	UDP glucuronosyltransferase 1 family, polypeptide A6A (Ugt1a6a)	94284
724	**Hspb7**	3.64	221	1.718	3.29	1.0794	1.20E-02	1.922	heat shock protein family, member 7 (cardiovascular) (Hspb7)	29818
725	**Ankrd23**	3.63	1193	1.641	3.12	0.9444	1.03E-02	1.989	ankyrin repeat domain 23 (Ankrd23)	78321
726	**H2ac14-ps**	3.62	216	0.764	1.70	0.2397	1.41E-03	2.852	H2A clustered histone 14, pseudogene (H2ac14-ps)	319174
727	**Abcb4**	3.60	65	1.500	2.83	0.7225	7.94E-03	2.100	ATP-binding cassette, sub-family B member 4 (Abcb4)	18670
728	**Rps18-ps5**	3.59	209	0.790	1.73	0.2539	1.60E-03	2.796	ribosomal protein S18, pseudogene 5 (Rps18-ps5)	100042388
729	**Gm22634**	3.58	2259	1.478	2.79	0.7044	7.86E-03	2.104	predicted gene, 22634 (Gm22634)	115488060
730	**Cks2**	3.58	257	0.773	1.71	0.2451	1.55E-03	2.809	CDC28 protein kinase regulatory subunit 2 (Cks2)	66197
731	**Nnmt**	3.58	74	1.145	2.21	0.4399	3.71E-03	2.430	nicotinamide N-methyltransferase (Nnmt)	18113
732	**Chl1**	3.57	1648	1.191	2.28	0.4703	4.17E-03	2.380	cell adhesion molecule L1-like (Chl1)	12661
733	**Agtr2**	3.56	68	1.340	2.53	0.5746	6.00E-03	2.222	angiotensin II receptor, type 2 (Agtr2)	11609
734	**Prl2c5**	3.55	120	1.072	2.10	0.3975	3.29E-03	2.483	prolactin family 2, subfamily c, member 5 (Prl2c5)	107849
735	**Klra4**	3.54	482	0.939	1.92	0.3280	2.51E-03	2.601	killer cell lectin-like receptor, subfamily A, member 4 (Klra4)	16635
736	**Ulbp1**	3.53	413	0.791	1.73	0.2564	1.81E-03	2.741	UL16 binding protein 1 (Ulbp1)	77777
737	**Rps19-ps2**	3.53	72	1.089	2.13	0.4128	3.65E-03	2.437	ribosomal protein S19, pseudogene 2 (Rps19-ps2)	667525
738	**Gm22614**	3.52	765	1.581	2.99	0.9119	1.14E-02	1.943	predicted gene, 22614 (Gm22614)	115489778

739	**Raet1a**	3.50	115	1.005	2.01	0.3656	3.18E-03	2.498	retinoic acid early transcript 1, alpha (Raet1a)	19368
740	**Plpp1**	3.48	256	0.786	1.72	0.2592	2.01E-03	2.696	phospholipid phosphatase 1 (Plpp1)	19012
741	**Gm26447**	3.48	167	1.121	2.17	0.4437	4.35E-03	2.361	predicted gene, 26447 (Gm26447)	11548868 9
742	**Fas**	3.48	64	1.199	2.30	0.4908	5.21E-03	2.283	Fas cell surface death receptor (Fas)	14102
743	**Clcn1**	3.47	89	1.403	2.64	0.7132	8.57E-03	2.067	chloride channel, voltage-sensitive 1 (Clcn1)	12723
744	**Plac8**	3.47	324	0.993	1.99	0.3711	3.34E-03	2.476	placenta-specific 8 (Plac8)	231507
745	**Gm17936**	3.46	85	1.039	2.05	0.3932	3.83E-03	2.417	predicted gene, 17936 (Gm17936)	10041613 8
746	**Plac9**	3.44	243	0.877	1.84	0.3082	2.74E-03	2.562	placenta specific 9 (Plac9)	211623
747	**Adgre4**	3.43	29	1.444	2.72	0.7359	1.02E-02	1.989	adhesion G protein-coupled receptor E4 (Adgre4)	52614
748	**Gm13215**	3.43	133	0.881	1.84	0.3076	2.83E-03	2.549	predicted gene 13215 (Gm13215)	664894
749	**Cd4**	3.43	105	0.983	1.98	0.3627	3.59E-03	2.444	CD4 antigen (Cd4)	12504
750	**Scn3b**	3.42	31	1.537	2.90	0.8434	1.31E-02	1.882	sodium channel, voltage-gated, type III, beta (Scn3b)	235281
751	**Usp13**	3.41	460	0.890	1.85	0.3163	3.00E-03	2.523	ubiquitin specific peptidase 13 (isopeptidase T-3) (Usp13)	72607
752	**Il1b**	3.41	669	0.789	1.73	0.2646	2.39E-03	2.621	interleukin 1 beta (Il1b)	16176
753	**Gm8942**	3.40	71	1.079	2.11	0.4275	4.74E-03	2.324	predicted gene 8942 (Gm8942)	668041
754	**Miat**	3.39	41	1.416	2.67	0.7494	1.06E-02	1.976	myocardial infarction associated transcript (non-protein coding) (Miat)	330166
755	**A530064D 06Rik**	3.38	58	1.132	2.19	0.4677	5.61E-03	2.251	RIKEN cDNA A530064D06 gene (A530064D06Rik)	328830
756	**Rps19-ps1**	3.37	69	1.065	2.09	0.4236	4.93E-03	2.307	ribosomal protein S19, pseudogene 1 (Rps19-ps1)	10004326 9
757	**Gm24924**	3.37	1038	1.393	2.63	0.7306	1.06E-02	1.976	predicted gene, 24924 (Gm24924)	11548899 1
758	**Gria1**	3.36	105	0.970	1.96	0.3650	4.04E-03	2.393	glutamate receptor, ionotropic, AMPA1 (alpha 1) (Gria1)	14799
759	**Gm26244**	3.35	1056	1.409	2.66	0.7615	1.14E-02	1.945	predicted gene, 26244 (Gm26244)	11548900 0
760	**Mr1**	3.35	261	0.827	1.77	0.2878	3.02E-03	2.520	major histocompatibility complex, class I-related (Mr1)	15064
761	**Btla**	3.35	51	1.192	2.28	0.5205	7.00E-03	2.155	B and T lymphocyte associated (Btla)	208154
762	**Kif1a**	3.34	103	0.996	1.99	0.3873	4.52E-03	2.345	kinesin family member 1A (Kif1a)	16560
763	**Fam13a**	3.34	54	1.163	2.24	0.4988	6.64E-03	2.178	family with sequence similarity 13, member A (Fam13a)	58909
764	**Omd**	3.33	73	1.064	2.09	0.4305	5.41E-03	2.267	Osteomodulin (Omd)	27047

765	**G0s2**	3.33	44	1.264	2.40	0.5904	8.64E-03	2.064	G0/G1 switch gene 2 (G0s2)	14373
766	**Hs6st2**	3.32	479	0.795	1.74	0.2754	2.97E-03	2.527	heparan sulfate 6-O-sulfotransferase 2 (Hs6st2)	50786
767	**Cd48**	3.32	154	0.819	1.76	0.2867	3.16E-03	2.500	CD48 antigen (Cd48)	12506
768	**Cacnb1**	3.31	100	1.129	2.19	0.4841	6.53E-03	2.185	calcium channel, voltage-dependent, beta 1 subunit (Cacnb1)	12295
769	**LOC115489800**	3.31	752	1.492	2.81	0.9855	1.51E-02	1.821	No gene name found in the DAVID Knowledgebase	0
770	**Gm22513**	3.30	2704	1.347	2.54	0.7139	1.11E-02	1.954	predicted gene, 22513 (Gm22513)	115488051
771	**Kcnma1**	3.29	330	1.011	2.02	0.4025	5.25E-03	2.280	potassium large conductance calcium-activated channel, subfamily M, alpha member 1 (Kcnma1)	16531
772	**Gm26110**	3.29	37	1.363	2.57	0.7309	1.19E-02	1.923	predicted gene, 26110 (Gm26110)	115487642
773	**Fez1**	3.28	70	1.074	2.11	0.4450	6.22E-03	2.206	fasciculation and elongation protein zeta 1 (Fez1)	235180
774	**Mrpl14**	3.27	189	0.762	1.70	0.2628	3.08E-03	2.512	mitochondrial ribosomal protein L14 (Mrpl14)	68463
775	**Lipt1**	3.27	104	0.993	1.99	0.3962	5.30E-03	2.276	lipoyltransferase 1 (Lipt1)	623661
776	**Matn2**	3.27	814	0.769	1.70	0.2662	3.16E-03	2.500	matrilin 2 (Matn2)	17181
777	**Eda2r**	3.26	157	0.875	1.83	0.3231	4.09E-03	2.388	ectodysplasin A2 receptor (Eda2r)	245527
778	**Sec16b**	3.26	205	0.773	1.71	0.2689	3.24E-03	2.490	SEC16 homolog B, endoplasmic reticulum export factor (Sec16b)	89867
779	**Alox5ap**	3.25	359	0.800	1.74	0.2856	3.52E-03	2.453	arachidonate 5-lipoxygenase activating protein (Alox5ap)	11690
780	**Jph1**	3.25	404	0.989	1.99	0.4012	5.53E-03	2.257	junctophilin 1 (Jph1)	57339
781	**Slc2a13**	3.24	316	0.792	1.73	0.2793	3.53E-03	2.452	solute carrier family 2 (facilitated glucose transporter), member 13 (Slc2a13)	239606
782	**Mlf1**	3.24	63	1.302	2.47	0.6991	1.15E-02	1.939	myeloid leukemia factor 1 (Mlf1)	17349
783	**Klf15**	3.22	25	1.671	3.19	1.2780	2.86E-02	1.544	Kruppel-like transcription factor 15 (Klf15)	66277
784	**Ndufb4c**	3.21	80	1.002	2.00	0.4084	6.14E-03	2.212	NADH:ubiquinone oxidoreductase subunit B4C (Ndufb4c)	100041273
785	**Chodl**	3.20	34	1.452	2.74	1.3651	1.79E-02	1.747	Chondrolectin (Chodl)	246048
786	**Snx22**	3.20	205	0.773	1.71	0.2752	3.76E-03	2.425	sorting nexin 22 (Snx22)	382083
787	**Cyp2j9**	3.19	35	1.504	2.84	1.0932	2.06E-02	1.686	cytochrome P450, family 2, subfamily j, polypeptide 9 (Cyp2j9)	74519

788	**Gm5854**	3.18	90	0.924	1.90	0.3631	5.51E-03	2.258	predicted gene 5854 (Gm5854)	545567
789	**Sult1a1**	3.17	103	0.930	1.90	0.3706	5.73E-03	2.242	sulfotransferase family 1A, phenol-preferring, member 1 (Sult1a1)	20887
790	**Zfp943**	3.17	418	0.904	1.87	0.3537	5.42E-03	2.266	zinc finger prtoein 943 (Zfp943)	74670
791	**Ecm2**	3.17	171	0.813	1.76	0.2994	4.43E-03	2.354	extracellular matrix protein 2, female organ and adipocyte specific (Ecm2)	407800
792	**Prxl2b**	3.16	54	1.223	2.33	0.6238	1.15E-02	1.940	peroxiredoxin like 2B (Prxl2b)	66469
793	**Cyp4b1**	3.16	29	1.414	2.66	0.8406	1.79E-02	1.747	cytochrome P450, family 4, subfamily b, polypeptide 1 (Cyp4b1)	13120
794	**Chp2**	3.15	30	1.314	2.49	0.7531	1.47E-02	1.833	calcineurin-like EF hand protein 2 (Chp2)	70261
795	**Rnu1b1**	3.14	1100	1.448	2.73	1.3911	2.02E-02	1.694	U1b1 small nuclear RNA (Rnu1b1)	19844
796	**Gm4335**	3.12	145	0.809	1.75	0.3017	4.93E-03	2.307	predicted gene 4335 (Gm4335)	100043283
797	**Gm54215**	3.11	57	1.223	2.33	0.6398	1.29E-02	1.889	predicted gene, 54215 (Gm54215)	118568644
798	**Gm46344**	3.11	88	1.176	2.26	0.5889	1.16E-02	1.935	predicted gene, 46344 (Gm46344)	108167994
799	**Gm23238**	3.10	574	1.351	2.55	0.9177	1.78E-02	1.750	predicted gene, 23238 (Gm23238)	115489085
800	**Gm10290**	3.10	60	1.029	2.04	0.4539	8.52E-03	2.070	predicted pseudogene 10290 (Gm10290)	100039258
801	**Lgi3**	3.09	36	1.427	2.69	1.1799	2.19E-02	1.660	leucine-rich repeat LGI family, member 3 (Lgi3)	213469
802	**Asb5**	3.08	89	1.246	2.37	0.7458	1.48E-02	1.831	ankyrin repeat and SOCs box-containing 5 (Asb5)	76294
803	**Acss3**	3.07	102	0.998	2.00	0.4353	8.49E-03	2.071	acyl-CoA synthetase short-chain family member 3 (Acss3)	380660
804	**Serpina3g**	3.07	61	1.059	2.08	0.4847	9.78E-03	2.010	serine (or cysteine) peptidase inhibitor, clade A, member 3G (Serpina3g)	20715
805	**Il1r2**	3.06	253	0.751	1.68	0.2748	4.86E-03	2.313	interleukin 1 receptor, type II (Il1r2)	16178
806	**Aff3**	3.06	70	1.009	2.01	0.4468	8.81E-03	2.055	AF4/FMR2 family, member 3 (Aff3)	16764
807	**Slc43a1**	3.05	76	1.047	2.07	0.4841	9.81E-03	2.008	solute carrier family 43, member 1 (Slc43a1)	72401
808	**Gm24830**	3.04	610	1.344	2.54	1.0486	2.02E-02	1.694	predicted gene, 24830 (Gm24830)	115489789
809	**Slc10a6**	3.04	41	1.216	2.32	0.6997	1.51E-02	1.821	solute carrier family 10 (sodium/bile acid cotransporter family), member 6 (Slc10a6)	75750

810	**Arhgap20**	3.04	84	1.196	2.29	0.7014	1.45E-02	1.840	Rho GTPase activating protein 20 (Arhgap20)	244867
811	**Pcdhga3**	3.02	150	0.782	1.72	0.2980	5.73E-03	2.242	protocadherin gamma subfamily A, 3 (Pcdhga3)	93711
812	**P2ry10**	3.02	120	1.060	2.09	0.5039	1.09E-02	1.963	purinergic receptor P2Y, G-protein coupled 10 (P2ry10)	78826
813	**AW551984**	3.02	184	0.880	1.84	0.3590	7.30E-03	2.136	expressed sequence AW551984 (AW551984)	244810
814	**Galnt17**	3.00	172	0.834	1.78	0.3326	6.77E-03	2.170	polypeptide N-acetylgalactosaminyltransferase 17 (Galnt17)	212996
815	**Gm10177**	2.99	69	1.004	2.01	0.4592	1.02E-02	1.990	predicted gene 10177 (Gm10177)	108167643
816	**Ank1**	2.99	188	1.091	2.13	0.5764	1.26E-02	1.901	ankyrin 1, erythroid (Ank1)	11733
817	**Rpl18-ps2**	2.97	123	0.810	1.75	0.3212	6.85E-03	2.164	ribosomal protein L18, pseudogene 2 (Rpl18-ps2)	652986
818	**Enpep**	2.96	96	0.924	1.90	0.4052	9.12E-03	2.040	glutamyl aminopeptidase (Enpep)	13809
819	**Gapt**	2.96	41	1.136	2.20	0.6183	1.50E-02	1.824	Grb2-binding adaptor, transmembrane (Gapt)	238875
820	**Hsd3b7**	2.95	534	0.840	1.79	0.3437	7.72E-03	2.112	hydroxy-delta-5-steroid dehydrogenase, 3 beta- and steroid delta-isomerase 7 (Hsd3b7)	101502
821	**Tpbgl**	2.95	58	1.261	2.40	1.0043	2.05E-02	1.687	trophoblast glycoprotein-like (Tpbgl)	100503386
822	**Gm12416**	2.94	98	0.892	1.86	0.3810	8.89E-03	2.051	predicted gene 12416 (Gm12416)	668010
823	**Dio3**	2.94	32	1.182	2.27	0.6876	1.74E-02	1.761	deiodinase, iodothyronine type III (Dio3)	107585
824	**Fgf10**	2.94	65	0.991	1.99	0.4634	1.13E-02	1.949	fibroblast growth factor 10 (Fgf10)	14165
825	**Gm12791**	2.94	48	1.051	2.07	0.5260	1.30E-02	1.885	predicted gene 12791 (Gm12791)	665362
826	**Ccl2**	2.94	319	0.805	1.75	0.3217	7.41E-03	2.130	C-C motif chemokine ligand 2 (Ccl2)	20296
827	**Aqp2**	2.93	28	1.198	2.29	0.7336	1.87E-02	1.729	aquaporin 2 (Aqp2)	11827
828	**Galnt5**	2.93	28	1.269	2.41	0.8875	2.21E-02	1.656	polypeptide N-acetylgalactosaminyltransferase 5 (Galnt5)	241391
829	**Slc36a2**	2.91	38	1.172	2.25	0.7082	1.83E-02	1.738	solute carrier family 36 (proton/amino acid symporter), member 2 (Slc36a2)	246049
830	**Rtn4rl2**	2.90	66	0.995	1.99	0.4794	1.24E-02	1.906	reticulon 4 receptor-like 2 (Rtn4rl2)	269295
831	**Lilra6**	2.88	71	0.977	1.97	0.4645	1.24E-02	1.907	leukocyte immunoglobulin-like receptor, subfamily A (with TM domain), member 6 (Lilra6)	18726
832	**Crispld2**	2.88	505	0.806	1.75	0.3322	8.51E-03	2.070	cysteine-rich secretory protein LCCL domain containing 2 (Crispld2)	78892
833	**Jam3**	2.87	111	0.858	1.81	0.3708	9.68E-03	2.014	junction adhesion molecule 3 (Jam3)	83964

834	**Ccdc8**	2.87	114	0.791	1.73	0.3229	8.37E-03	2.077	coiled-coil domain containing 8 (Ccdc8)	434130
835	**Lmod3**	2.85	48	1.255	2.39	1.1534	2.53E-02	1.597	leiomodin 3 (fetal) (Lmod3)	320502
836	**Sytl2**	2.84	137	0.770	1.71	0.3137	8.50E-03	2.071	synaptotagmin-like 2 (Sytl2)	83671
837	**Hspb6**	2.83	412	0.870	1.83	0.3976	1.10E-02	1.957	heat shock protein, alpha-crystallin-related, B6 (Hspb6)	243912
838	**Macrod1**	2.79	101	0.899	1.86	0.4256	1.28E-02	1.893	mono-ADP ribosylhydrolase 1 (Macrod1)	107227
839	**Gm13882**	2.79	91	0.846	1.80	0.3770	1.14E-02	1.945	predicted gene 13882 (Gm13882)	100043349
840	**Il6**	2.77	78	0.945	1.93	0.4702	1.50E-02	1.823	interleukin 6 (Il6)	16193
841	**Sorcs1**	2.77	58	1.006	2.01	0.5437	1.74E-02	1.759	sortilin-related VPS10 domain containing receptor 1 (Sorcs1)	58178
842	**Cd209a**	2.75	51	1.068	2.10	0.6824	2.06E-02	1.686	CD209a antigen (Cd209a)	170786
843	**Sptbn5**	2.75	33	1.258	2.39	1.2406	3.23E-02	1.491	spectrin beta, non-erythrocytic 5 (Sptbn5)	640524
844	**Scin**	2.72	119	0.838	1.79	0.3865	1.32E-02	1.881	Scinderin (Scin)	20259
845	**Rpl19-ps12**	2.72	27	1.180	2.27	1.0145	2.89E-02	1.539	ribosomal protein L19, pseudogene 12 (Rpl19-ps12)	208428
846	**Avpr1a**	2.71	47	0.990	1.99	0.5550	1.89E-02	1.724	arginine vasopressin receptor 1A (Avpr1a)	54140
847	**Npr2**	2.69	131	0.806	1.75	0.3651	1.30E-02	1.887	natriuretic peptide receptor 2 (Npr2)	230103
848	**1110065P20Rik**	2.68	68	0.889	1.85	0.4505	1.64E-02	1.786	RIKEN cDNA 1110065P20 gene (1110065P20Rik)	68920
849	**Pcdh10**	2.67	82	0.857	1.81	0.4160	1.56E-02	1.808	protocadherin 10 (Pcdh10)	18526
850	**Jph2**	2.66	264	1.101	2.15	1.1663	2.74E-02	1.562	junctophilin 2 (Jph2)	59091
851	**Saa3**	2.66	73	0.883	1.84	0.4478	1.66E-02	1.779	serum amyloid A 3 (Saa3)	20210
852	**Adcy2**	2.66	130	0.815	1.76	0.3837	1.43E-02	1.846	adenylate cyclase 2 (Adcy2)	210044
853	**Ackr1**	2.65	44	1.037	2.05	0.7043	2.43E-02	1.614	atypical chemokine receptor 1 (Duffy blood group) (Ackr1)	13349
854	**Gpr84**	2.65	34	1.041	2.06	0.6774	2.45E-02	1.611	G protein-coupled receptor 84 (Gpr84)	80910
855	**Aldh1a2**	2.63	140	0.839	1.79	0.4154	1.63E-02	1.787	aldehyde dehydrogenase family 1, subfamily A2 (Aldh1a2)	19378
856	**Gm5507**	2.63	50	0.957	1.94	0.5510	2.14E-02	1.669	predicted gene 5507 (Gm5507)	433184
857	**Maob**	2.62	45	1.023	2.03	0.7056	2.51E-02	1.600	monoamine oxidase B (Maob)	109731
858	**Lgr5**	2.61	296	0.861	1.82	0.4413	1.78E-02	1.750	leucine rich repeat containing G protein coupled receptor 5 (Lgr5)	14160

859	**Gm3695**	2.60	75	0.861	1.82	0.4424	1.81E-02	1.741	predicted gene 3695 (Gm3695)	100042148
860	**Cavin4**	2.59	71	0.952	1.93	0.5713	2.28E-02	1.643	caveolae associated 4 (Cavin4)	68016
861	**Gm8349**	2.59	45	0.955	1.94	0.5715	2.30E-02	1.638	predicted gene 8349 (Gm8349)	666891
862	**Chac1**	2.59	88	0.960	1.95	0.5997	2.34E-02	1.631	ChaC, cation transport regulator 1 (Chac1)	69065
863	**Fancb**	2.59	120	0.755	1.69	0.3433	1.47E-02	1.834	Fanconi anemia, complementation group B (Fancb)	237211
864	**Ndufb4b**	2.59	93	0.780	1.72	0.3663	1.55E-02	1.809	NADH:ubiquinone oxidoreductase subunit B4B (Ndufb4b)	100042503
865	**Gldn**	2.58	151	0.908	1.88	0.5180	2.11E-02	1.675	Gliomedin (Gldn)	235379
866	**Adssl1**	2.57	389	1.028	2.04	0.9014	2.89E-02	1.539	No gene name found in the DAVID Knowledgebase	0
867	**Chchd10**	2.55	118	0.952	1.93	0.6478	2.54E-02	1.595	coiled-coil-helix-coiled-coil-helix domain containing 10 (Chchd10)	103172
868	**Tll1**	2.55	288	0.798	1.74	0.3936	1.79E-02	1.748	tolloid-like (Tll1)	21892
869	**Nfasc**	2.51	110	0.983	1.98	0.7261	2.95E-02	1.531	Neurofascin (Nfasc)	269116
870	**Gm7819**	2.51	53	0.931	1.91	0.5815	2.64E-02	1.579	predicted gene 7819 (Gm7819)	665845
871	**Rpl21-ps7**	2.51	62	0.916	1.89	0.5574	2.57E-02	1.591	ribosomal protein L21, pseudogene 7 (Rpl21-ps7)	545226
872	**Cryaa**	2.51	261	1.020	2.03	1.6009	3.27E-02	1.486	crystallin, alpha A (Cryaa)	12954
873	**Gnpnat1**	2.49	116	0.912	1.88	0.5996	2.63E-02	1.580	glucosamine-phosphate N-acetyltransferase 1 (Gnpnat1)	54342
874	**Htr2a**	2.49	151	0.781	1.72	0.3939	1.97E-02	1.705	5-hydroxytryptamine (serotonin) receptor 2A (Htr2a)	15558
875	**Pdk4**	2.48	537	0.980	1.97	1.9529	3.14E-02	1.504	pyruvate dehydrogenase kinase, isoenzyme 4 (Pdk4)	27273
876	**Commd5**	2.48	142	0.808	1.75	0.4311	2.12E-02	1.673	COMM domain containing 5 (Commd5)	66398
877	**Gm6316**	2.48	77	0.797	1.74	0.4124	2.09E-02	1.680	predicted pseudogene 6316 (Gm6316)	622339
878	**Fgf14**	2.47	29	1.046	2.06	1.1685	3.76E-02	1.425	fibroblast growth factor 14 (Fgf14)	14169
879	**Il11ra2**	2.47	50	0.883	1.84	0.5307	2.59E-02	1.587	interleukin 11 receptor subunit alpha 2 (Il11ra2)	16158
880	**A2m**	2.47	82	0.895	1.86	0.5606	2.67E-02	1.573	alpha-2-macroglobulin (A2m)	232345
881	**Papss2**	2.45	237	0.764	1.70	0.3863	2.06E-02	1.686	3'-phosphoadenosine 5'-phosphosulfate synthase 2 (Papss2)	23972
882	**Gm4865**	2.44	37	0.936	1.91	0.6654	3.10E-02	1.509	predicted gene 4865(Gm4865)	231069
883	**Ccr7**	2.44	62	0.864	1.82	0.5312	2.67E-02	1.573	C-C motif chemokine receptor 7 (Ccr7)	12775
884	**Nr1h3**	2.42	81	0.796	1.74	0.4299	2.37E-02	1.625	nuclear receptor subfamily 1, group H, member 3 (Nr1h3)	22259
885	**Klhl33**	2.41	94	0.896	1.86	0.6524	3.09E-02	1.510	kelch-like 33 (Klhl33)	546611

886	**Slc16a9**	2.40	62	0.867	1.82	0.5501	2.96E-02	1.529	solute carrier family 16 (monocarboxylic acid transporters), member 9(Slc16a9)	66859
887	**Il2ra**	2.39	72	0.790	1.73	0.4355	2.52E-02	1.598	interleukin 2 receptor, alpha chain (Il2ra)	16184
888	**F830016B08Rik**	2.38	340	0.774	1.71	0.4331	2.50E-02	1.603	RIKEN cDNA F830016B08 gene (F830016B08Rik)	240328
889	**Mmp2**	2.38	5934	0.835	1.78	0.5037	2.88E-02	1.541	matrix metallopeptidase 2 (Mmp2)	17390
890	**Acvr1c**	2.37	45	0.924	1.90	0.7318	3.55E-02	1.449	activin A receptor, type IC (Acvr1c)	269275
891	**Aldoa**	2.36	13349	0.798	1.74	0.4716	2.75E-02	1.561	aldolase A, fructose-bisphosphate (Aldoa)	11674
892	**Trex1**	2.35	115	0.751	1.68	0.4000	2.50E-02	1.603	three prime repair exonuclease 1 (Trex1)	22040
893	**Igsf10**	2.35	1912	0.901	1.87	0.7146	3.58E-02	1.446	immunoglobulin superfamily, member 10 (Igsf10)	242050
894	**Rab3b**	2.35	66	0.838	1.79	0.5357	3.11E-02	1.507	RAB3B, member RAS oncogene family (Rab3b)	69908
895	**Cth**	2.34	28	0.920	1.89	0.7936	3.77E-02	1.423	cystathionine gamma lyase (Cth)	107869
896	**Slamf7**	2.34	75	0.885	1.85	0.6500	3.51E-02	1.454	SLAM family member 7 (Slamf7)	75345
897	**Tlcd4**	2.31	98	0.803	1.74	0.5031	3.09E-02	1.510	TLC domain containing 4 (Tlcd4)	99887
898	**Rai2**	2.31	30	0.910	1.88	0.8867	4.01E-02	1.397	retinoic acid induced 2 (Rai2)	24004
899	**Slc13a3**	2.30	69	0.814	1.76	0.5179	3.29E-02	1.483	solute carrier family 13 (sodium-dependent dicarboxylate transporter), member 3 (Slc13a3)	114644
900	**Rps15a-ps7**	2.29	46	0.838	1.79	0.5729	3.50E-02	1.456	ribosomal protein S15A, pseudogene 7 (Rps15a-ps7)	100043843
901	**Pparg**	2.28	69	0.783	1.72	0.4746	3.17E-02	1.499	peroxisome proliferator activated receptor gamma (Pparg)	19016
902	**Siglecg**	2.26	31	0.887	1.85	0.7972	4.19E-02	1.377	sialic acid binding Ig-like lectin G (Siglecg)	243958
903	**Gfra1**	2.24	1068	0.806	1.75	0.5591	3.68E-02	1.434	glial cell line derived neurotrophic factor family receptor alpha 1 (Gfra1)	14585
904	**Bmpr1b**	2.23	79	0.807	1.75	0.5577	3.75E-02	1.426	bone morphogenetic protein receptor, type 1B (Bmpr1b)	12167
905	**Rps4l**	2.21	59	0.757	1.69	0.4735	3.50E-02	1.456	ribosomal protein S4-like (Rps4l)	66184
906	**C1qtnf9**	2.20	31	0.829	1.78	1.1647	4.27E-02	1.369	C1q and tumor necrosis factor related protein 9 (C1qtnf9)	239126
907	**Limch1**	2.19	381	0.824	1.77	0.9311	4.28E-02	1.369	LIM and calponin homology domains 1 (Limch1)	77569
908	**Rbm20**	2.18	96	0.794	1.73	0.5902	4.11E-02	1.386	RNA binding motif protein 20 (Rbm20)	73713
909	**Rpl36-ps12**	2.17	37	0.810	1.75	0.6693	4.37E-02	1.360	ribosomal protein L36, pseudogene 12 (Rpl36-ps12)	100043718
910	**Rps19-ps7**	2.16	21	0.836	1.78	1.1470	4.72E-02	1.326	ribosomal protein S19, pseudogene 7 (Rps19-ps7)	269365

911	**Angpt1**	2.15	586	0.792	1.73	0.6534	4.44E-02	1.353	angiopoietin 1 (Angpt1)	11600
912	**Gm7027**	2.11	46	0.763	1.70	0.5805	4.52E-02	1.344	predicted gene 7027 (Gm7027)	630138
913	**Snora73b**	2.08	51	0.761	1.69	0.6218	4.83E-02	1.316	small nucleolar RNA, H/ACA box 73b (Snora73b)	10030694<br>5
914	**Mexis**	2.07	47	0.753	1.69	0.5754	4.77E-02	1.322	macrophage expressed LXRa(NR1H3)-dependent amplifier of Abca1 transcription lncRNA (Mexis)	381524

Downregulated DEGs

Rank	Gene	Manhattan distance	baseMean	log2FoldChange	FC	lfcSE	padj	minus-log10_padj	Gene Name found in the DAVID Knowledgebase	ENTREZ Gene ID NO.
		|ΔX| +|ΔY|		ΔX				ΔY		
1	**Snord15b**	31.09	205	-3.317	0.10	0.2912	1.69E-28	27.771	small nucleolar RNA, C/D box 15B (Snord15b)	449631
2	**Nav2**	26.01	5809	-1.170	0.44	0.1082	1.45E-25	24.839	neuron navigator 2 (Nav2)	78286
3	**Neat1**	25.31	92593	-1.439	0.37	0.1361	1.33E-24	23.876	nuclear paraspeckle assembly transcript 1 (non-protein coding) (Neat1)	66961
4	**Chka**	24.72	3146	-1.173	0.44	0.1116	2.81E-24	23.552	choline kinase alpha (Chka)	12660
5	**9230105E05Rik**	22.84	944	-1.422	0.37	0.1424	3.83E-22	21.417	RIKEN cDNA 9230105E05 gene (9230105E05Rik)	320626
6	**Ltbp3**	22.49	9976	-0.965	0.51	0.0960	2.98E-22	21.526	latent transforming growth factor beta binding protein 3 (Ltbp3)	16998
7	**Firre**	21.60	2150	-1.084	0.47	0.1107	3.01E-21	20.521	functional intergenic repeating RNA element (Firre)	103012
8	**Clcf1**	20.31	2245	-1.043	0.49	0.1101	5.43E-20	19.265	cardiotrophin-like cytokine factor 1 (Clcf1)	56708
9	**Irs1**	19.45	9192	-0.832	0.56	0.0890	2.41E-19	18.617	insulin receptor substrate 1 (Irs1)	16367
10	**Zfhx3**	18.90	12355	-0.902	0.54	0.0981	9.93E-19	18.003	zinc finger homeobox 3 (Zfhx3)	11906
11	**Elf3**	17.40	521	-1.427	0.37	0.1662	1.06E-16	15.974	E74-like factor 3 (Elf3)	13710
12	**Muc4**	17.21	232	-1.979	0.25	0.2369	5.90E-16	15.229	mucin 4 (Muc4)	140474
13	**Pitpnm3**	17.12	1915	-1.500	0.35	0.1771	2.38E-16	15.624	PITPNM family member 3 (Pitpnm3)	327958
14	**Gm24265**	17.03	3228	-1.148	0.45	0.1340	1.31E-16	15.881	predicted gene, 24265 (Gm24265)	115490317
15	**Mgat3**	16.74	2021	-1.031	0.49	0.1207	1.94E-16	15.712	mannoside acetylglucosaminyltransferase 3 (Mgat3)	17309
16	**2610035D17Rik**	16.44	501	-1.669	0.31	0.2032	1.71E-15	14.766	RIKEN cDNA 2610035D17 gene (2610035D17Rik)	72386
17	**Als2cl**	16.39	2457	-1.141	0.45	0.1358	5.59E-16	15.253	ALS2 C-terminal like (Als2cl)	235633
18	**Tnnt2**	15.86	1611	-1.423	0.37	0.1750	3.64E-15	14.439	troponin T2, cardiac (Tnnt2)	21956
19	**Kcnq1ot1**	15.68	9652	-1.090	0.47	0.1329	2.60E-15	14.585	KCNQ1 overlapping transcript 1 (Kcnq1ot1)	63830
20	**Spns2**	15.64	2523	-0.967	0.51	0.1172	2.14E-15	14.669	SPNS lysolipid transporter 2, sphingosine-1-phosphate (Spns2)	216892
21	**Strc**	15.35	927	-1.188	0.44	0.1471	6.87E-15	14.163	Stereocilin (Strc)	140476
22	**Fat2**	15.27	813	-1.867	0.27	0.2403	3.97E-14	13.401	FAT atypical cadherin 2 (Fat2)	245827

23	**Col7a1**	15.14	8390	-1.191	0.44	0.1487	1.13E-14	13.947	collagen, type VII, alpha 1 (Col7a1)	12836
24	**Lama5**	14.88	9966	-1.196	0.44	0.1511	2.09E-14	13.679	laminin, alpha 5 (Lama5)	16776
25	**Gm25939**	14.87	2681	-1.582	0.33	0.2034	5.14E-14	13.289	predicted gene, 25939 (Gm25939)	115487691
26	**Clmn**	14.67	1790	-1.356	0.39	0.1742	4.87E-14	13.312	Calmin (Clmn)	94040
27	**Rapgef3**	14.58	1545	-1.010	0.50	0.1276	2.69E-14	13.570	Rap guanine nucleotide exchange factor (GEF) 3 (Rapgef3)	223864
28	**Nbeal2**	14.16	3119	-1.149	0.45	0.1490	9.71E-14	13.013	neurobeachin-like 2 (Nbeal2)	235627
29	**Mir100hg**	14.15	12297	-0.830	0.56	0.1052	4.73E-14	13.325	Mir100 Mirlet7a-2 Mir125b-1 cluster host gene (Mir100hg)	73144
30	**S100a14**	13.95	704	-1.513	0.35	0.2020	3.67E-13	12.436	S100 calcium binding protein A14 (S100a14)	66166
31	**Wwc1**	13.84	1970	-1.147	0.45	0.1505	2.03E-13	12.692	WW, C2 and coiled-coil domain containing 1 (Wwc1)	211652
32	**Gan**	13.68	2294	-0.861	0.55	0.1115	1.50E-13	12.824	giant axonal neuropathy (Gan)	209239
33	**Rbm4**	13.51	2064	-0.832	0.56	0.1084	2.12E-13	12.674	RNA binding motif protein 4 (Rbm4)	19653
34	**Myh14**	13.25	1136	-1.848	0.28	0.2600	3.92E-12	11.406	myosin, heavy polypeptide 14 (Myh14)	71960
35	**Lrrtm2**	13.16	785	-1.033	0.49	0.1385	7.39E-13	12.131	leucine rich repeat transmembrane neuronal 2 (Lrrtm2)	107065
36	**Ltf**	13.15	685	-1.354	0.39	0.1854	1.60E-12	11.796	Lactotransferrin (Ltf)	17002
37	**Agrn**	12.92	7812	-1.285	0.41	0.1773	2.29E-12	11.639	Agrin (Agrn)	11603
38	**Cwh43**	12.76	1505	-0.980	0.51	0.1332	1.67E-12	11.777	cell wall biogenesis 43 C-terminal homolog (Cwh43)	231293
39	**Unc5b**	12.76	3303	-0.767	0.59	0.1025	1.02E-12	11.989	unc-5 netrin receptor B (Unc5b)	107449
40	**Nebl**	12.68	999	-0.969	0.51	0.1320	1.94E-12	11.712	Nebulette (Nebl)	74103
41	**Tslp**	12.40	806	-1.086	0.47	0.1511	4.82E-12	11.317	thymic stromal lymphopoietin (Tslp)	53603
42	**Slc4a4**	12.28	4338	-0.876	0.55	0.1208	3.92E-12	11.406	solute carrier family 4 (anion exchanger), member 4 (Slc4a4)	54403
43	**Arhgap27**	12.00	1330	-1.311	0.40	0.1898	2.07E-11	10.684	Rho GTPase activating protein 27 (Arhgap27)	544817
44	**Gm10389**	11.93	719	-1.026	0.49	0.1454	1.25E-11	10.904	predicted gene 10389 (Gm10389)	100038608
45	**Ppbp**	11.81	384	-1.331	0.40	0.1938	3.32E-11	10.479	pro-platelet basic protein (Ppbp)	57349
46	**Gm44502**	11.79	124	-4.289	0.05	0.7597	3.13E-08	7.505	predicted readthrough transcript (NMD candidate), 44502 (Gm44502)	100134861
47	**Cldn4**	11.79	2691	-0.921	0.53	0.1307	1.35E-11	10.869	claudin 4 (Cldn4)	12740
48	**Tns4**	11.76	7475	-0.862	0.55	0.1219	1.27E-11	10.896	tensin 4 (Tns4)	217169
49	**Col4a4**	11.55	1149	-1.008	0.50	0.1454	2.84E-11	10.546	collagen, type IV, alpha 4 (Col4a4)	12829
50	**Dnmt3a**	11.54	2857	-0.765	0.59	0.1083	1.68E-11	10.774	DNA methyltransferase 3A (Dnmt3a)	13435
51	**Prr15l**	11.49	261	-1.636	0.32	0.2479	1.41E-10	9.852	proline rich 15-like (Prr15l)	217138

52	**Szt2**	11.47	1365	-0.938	0.52	0.1353	2.96E-11	10.529	SZT2 subunit of KICSTOR complex (Szt2)	230676
53	**Kcnq1**	11.44	699	-1.107	0.46	0.1621	4.68E-11	10.330	potassium voltage-gated channel, subfamily Q, member 1 (Kcnq1)	16535
54	**Plekha6**	11.28	1035	-1.029	0.49	0.1511	5.60E-11	10.252	pleckstrin homology domain containing, family A member 6 (Plekha6)	240753
55	**Plekhs1**	11.25	432	-1.654	0.32	0.2544	2.53E-10	9.597	pleckstrin homology domain containing, family S member 1 (Plekhs1)	226245
56	**Pappa**	11.16	7828	-0.835	0.56	0.1214	4.68E-11	10.330	pregnancy-associated plasma protein A (Pappa)	18491
57	**Esrp1**	11.13	1404	-1.103	0.47	0.1642	9.46E-11	10.024	epithelial splicing regulatory protein 1 (Esrp1)	207920
58	**Plcd3**	11.13	1191	-1.205	0.43	0.1807	1.20E-10	9.922	phospholipase C, delta 3 (Plcd3)	72469
59	**Kmt2b**	11.12	2505	-0.802	0.57	0.1167	4.80E-11	10.319	lysine (K)-specific methyltransferase 2B (Kmt2b)	75410
60	**Pkhd1**	10.99	516	-1.501	0.35	0.2320	3.24E-10	9.489	polycystic kidney and hepatic disease 1 (Pkhd1)	241035
61	**Atg9b**	10.96	2620	-1.160	0.45	0.1754	1.60E-10	9.796	autophagy related 9B (Atg9b)	213948
62	**Zic2**	10.90	556	-1.105	0.46	0.1664	1.62E-10	9.791	zinc finger protein of the cerebellum 2 (Zic2)	22772
63	**Alox12**	10.61	343	-1.316	0.40	0.2052	5.11E-10	9.291	arachidonate 12-lipoxygenase (Alox12)	11684
64	**Spint2**	10.57	2507	-1.243	0.42	0.1933	4.68E-10	9.330	serine protease inhibitor, Kunitz type 2 (Spint2)	20733
65	**Plxnb1**	10.52	1271	-1.422	0.37	0.2253	7.90E-10	9.103	plexin B1 (Plxnb1)	235611
66	**Rbm47**	10.44	2213	-0.822	0.57	0.1238	2.38E-10	9.623	RNA binding motif protein 47 (Rbm47)	245945
67	**2900026A02Rik**	10.44	4388	-0.971	0.51	0.1486	3.37E-10	9.473	RIKEN cDNA 2900026A02 gene (2900026A02Rik)	243219
68	**St14**	10.42	1230	-1.165	0.45	0.1817	5.56E-10	9.255	suppression of tumorigenicity 14 (colon carcinoma) (St14)	19143
69	**Mab21l4**	10.41	554	-1.428	0.37	0.2274	1.06E-09	8.977	mab-21-like 4 (Mab21l4)	71874
70	**Adgrg2**	10.37	1865	-0.770	0.59	0.1158	2.53E-10	9.597	adhesion G protein-coupled receptor G2 (Adgrg2)	237175
71	**Syt8**	10.34	1005	-1.308	0.40	0.2075	9.21E-10	9.036	synaptotagmin VIII (Syt8)	55925
72	**Arhgef17**	10.31	3356	-0.755	0.59	0.1139	2.80E-10	9.553	Rho guanine nucleotide exchange factor 17 (Arhgef17)	207212
73	**Kmt2d**	10.20	9892	-0.761	0.59	0.1156	3.64E-10	9.439	lysine (K)-specific methyltransferase 2D (Kmt2d)	381022
74	**Mafg**	10.17	3029	-0.772	0.59	0.1177	4.02E-10	9.396	v-maf musculoaponeurotic fibrosarcoma oncogene family, protein G (avian) (Mafg)	17134
75	**Tmprss2**	10.17	219	-1.638	0.32	0.2695	2.95E-09	8.530	transmembrane protease, serine 2 (Tmprss2)	50528
76	**Gabbr1**	10.12	1060	-0.830	0.56	0.1275	5.11E-10	9.291	gamma-aminobutyric acid type B receptor subunit 1 (Gabbr1)	54393
77	**Safb2**	10.10	1674	-0.903	0.53	0.1401	6.40E-10	9.194	scaffold attachment factor B2 (Safb2)	224902
78	**Evpl**	9.85	988	-1.463	0.36	0.2432	4.09E-09	8.388	Envoplakin (Evpl)	14027
79	**Prom2**	9.69	265	-1.706	0.31	0.2925	1.04E-08	7.982	prominin 2 (Prom2)	192212

80	**Myo1d**	9.69	2769	-0.861	0.55	0.1365	1.49E-09	8.826	myosin ID (Myo1d)	338367
81	**Sh3rf1**	9.44	3244	-0.782	0.58	0.1247	2.21E-09	8.655	SH3 domain containing ring finger 1 (Sh3rf1)	59009
82	**Celsr2**	9.36	815	-1.354	0.39	0.2303	9.82E-09	8.008	cadherin, EGF LAG seven-pass G-type receptor 2 (Celsr2)	53883
83	**Jup**	9.36	5407	-0.795	0.58	0.1278	2.75E-09	8.561	junction plakoglobin (Jup)	16480
84	**Zfp827**	9.33	1776	-0.766	0.59	0.1225	2.73E-09	8.564	zinc finger protein 827 (Zfp827)	622675
85	**Sipa1l3**	9.30	4445	-0.756	0.59	0.1212	2.84E-09	8.546	signal-induced proliferation-associated 1 like 3 (Sipa1l3)	74206
86	**Celsr1**	9.28	1565	-1.124	0.46	0.1877	6.92E-09	8.160	cadherin, EGF LAG seven-pass G-type receptor 1 (Celsr1)	12614
87	**Ano9**	9.25	300	-1.639	0.32	0.2886	2.42E-08	7.616	anoctamin 9 (Ano9)	71345
88	**Gabrp**	9.23	1082	-1.011	0.50	0.1673	6.02E-09	8.221	gamma-aminobutyric acid type A receptor subunit pi (Gabrp)	216643
89	**Ehf**	9.14	320	-1.201	0.44	0.2040	1.14E-08	7.943	ets homologous factor (Ehf)	13661
90	**AI661453**	9.11	1320	-0.958	0.51	0.1590	6.99E-09	8.155	expressed sequence AI661453 (AI661453)	224833
91	**Elmo3**	9.06	555	-1.001	0.50	0.1675	8.72E-09	8.059	engulfment and cell motility 3 (Elmo3)	234683
92	**Eps8l2**	9.02	1148	-1.028	0.49	0.1733	1.01E-08	7.994	EPS8-like 2 (Eps8l2)	98845
93	**Cdh3**	9.01	897	-1.149	0.45	0.1965	1.38E-08	7.860	cadherin 3 (Cdh3)	12560
94	**Ppl**	9.00	1524	-1.007	0.50	0.1696	1.01E-08	7.995	Periplakin (Ppl)	19041
95	**Lratd2**	8.89	1017	-1.002	0.50	0.1701	1.31E-08	7.883	LRAT domain containing 1 (Lratd2)	399603
96	**Dsp**	8.86	4583	-1.203	0.43	0.2094	2.21E-08	7.655	Desmoplakin (Dsp)	109620
97	**B4galnt3**	8.85	1409	-1.008	0.50	0.1718	1.43E-08	7.843	beta-1,4-N-acetyl-galactosaminyl transferase 3 (B4galnt3)	330406
98	**Hap1**	8.85	668	-1.233	0.43	0.2154	2.43E-08	7.615	huntingtin-associated protein 1 (Hap1)	15114
99	**Cep170b**	8.79	3685	-0.763	0.59	0.1269	9.38E-09	8.028	centrosomal protein 170B (Cep170b)	217882
100	**Dsg2**	8.71	1953	-0.776	0.58	0.1299	1.17E-08	7.930	desmoglein 2 (Dsg2)	13511
101	**Irf6**	8.68	1190	-1.170	0.44	0.2056	3.10E-08	7.508	interferon regulatory factor 6 (Irf6)	54139
102	**Cldn3**	8.67	1187	-1.032	0.49	0.1787	2.33E-08	7.633	claudin 3 (Cldn3)	12739
103	**Lad1**	8.66	3664	-0.984	0.51	0.1691	2.11E-08	7.675	Ladinin (Lad1)	16763
104	**4930594C11Rik**	8.57	223	-1.466	0.36	0.2674	7.80E-08	7.108	RIKEN cDNA 4930594C11 gene (4930594C11Rik)	77633
105	**Jak3**	8.54	1065	-0.797	0.58	0.1351	1.80E-08	7.745	Janus kinase 3 (Jak3)	16453
106	**Srgap3**	8.53	1823	-0.793	0.58	0.1345	1.85E-08	7.732	SLIT-ROBO Rho GTPase activating protein 3 (Srgap3)	259302
107	**4933405E24Rik**	8.47	114	-1.776	0.29	0.3369	2.04E-07	6.691	RIKEN cDNA 4933405E24 gene (4933405E24Rik)	71056
108	**4933417O13Rik**	8.39	93	-2.068	0.24	0.4084	4.72E-07	6.326	RIKEN cDNA 4933417O13 gene (4933417O13Rik)	71153
109	**Ttll10**	8.39	95	-1.975	0.25	0.3850	3.83E-07	6.417	tubulin tyrosine ligase-like family, member 10 (Ttll10)	330010

110	Epn3	8.38	218	-1.623	0.32	0.3066	1.77E-07	6.752	epsin 3 (Epn3)	71889
111	Tmc4	8.36	987	-1.137	0.45	0.2041	6.02E-08	7.220	transmembrane channel-like gene family 4 (Tmc4)	353499
112	Notch1	8.35	2577	-0.851	0.55	0.1472	3.14E-08	7.503	notch 1 (Notch1)	18128
113	Ccn2	8.33	7473	-0.779	0.58	0.1339	2.78E-08	7.556	cellular communication network factor 2 (Ccn2)	14219
114	Itgb4	8.33	11709	-0.796	0.58	0.1371	2.90E-08	7.538	integrin beta 4 (Itgb4)	192897
115	Ptprf	8.27	2488	-0.985	0.51	0.1749	5.18E-08	7.286	protein tyrosine phosphatase receptor type F (Ptprf)	19268
116	Shank2	8.23	667	-1.183	0.44	0.2155	8.95E-08	7.048	SH3 and multiple ankyrin repeat domains 2 (Shank2)	210274
117	Tecpr1	8.23	1747	-0.822	0.57	0.1433	3.95E-08	7.403	tectonin beta-propeller repeat containing 1 (Tecpr1)	70381
118	A930037H05Rik	8.22	424	-1.017	0.49	0.1817	6.33E-08	7.198	RIKEN cDNA A930037H05 gene (A930037H05Rik)	78439
119	Usp43	8.17	461	-1.087	0.47	0.1964	8.20E-08	7.086	ubiquitin specific peptidase 43 (Usp43)	216835
120	Eps8l1	8.17	287	-1.248	0.42	0.2298	1.19E-07	6.925	EPS8-like 1 (Eps8l1)	67425
121	Erbb2	8.14	1680	-0.884	0.54	0.1565	5.54E-08	7.256	erb-b2 receptor tyrosine kinase 2 (Erbb2)	13866
122	Clca3b	8.13	686	-1.264	0.42	0.2347	1.35E-07	6.870	chloride channel accessory 3B (Clca3b)	229927
123	Skap1	8.08	549	-1.020	0.49	0.1843	8.68E-08	7.062	src family associated phosphoprotein 1 (Skap1)	78473
124	Cilp2	8.05	220	-1.614	0.33	0.3133	3.70E-07	6.432	cartilage intermediate layer protein 2 (Cilp2)	68709
125	AU018091	8.02	243	-1.651	0.32	0.3232	4.23E-07	6.373	expressed sequence AU018091 (AU018091)	245128
126	Patj	8.01	1683	-0.883	0.54	0.1578	7.38E-08	7.132	PATJ, crumbs cell polarity complex component (Patj)	12695
127	Unc13b	7.98	1270	-0.947	0.52	0.1711	9.25E-08	7.034	unc-13 homolog B (Unc13b)	22249
128	Sox11	7.95	1896	-0.972	0.51	0.1766	1.05E-07	6.979	SRY (sex determining region Y)-box 11 (Sox11)	20666
129	Cdh1	7.93	9980	-0.899	0.54	0.1622	9.31E-08	7.031	cadherin 1 (Cdh1)	12550
130	2610307P16Rik	7.91	646	-0.835	0.56	0.1491	8.34E-08	7.079	RIKEN cDNA 2610307P16 gene (2610307P16Rik)	72518
131	Mal2	7.89	448	-1.030	0.49	0.1894	1.39E-07	6.857	mal, T cell differentiation protein 2 (Mal2)	105853
132	Arhgap27os3	7.86	149	-1.908	0.27	0.3927	1.11E-06	5.957	Rho GTPase activating protein 27, opposite strand 3 (Arhgap27os3)	653030
133	Cdc42bpg	7.83	1185	-0.929	0.53	0.1693	1.25E-07	6.901	CDC42 binding protein kinase gamma (Cdc42bpg)	240505
134	Tacstd2	7.82	479	-0.987	0.50	0.1814	1.47E-07	6.833	tumor-associated calcium signal transducer 2 (Tacstd2)	56753
135	Sptbn2	7.78	684	-0.913	0.53	0.1667	1.37E-07	6.864	spectrin beta, non-erythrocytic 2 (Sptbn2)	20743
136	Cdk18	7.78	684	-0.915	0.53	0.1672	1.38E-07	6.860	cyclin dependent kinase 18 (Cdk18)	18557
137	Plekhn1	7.73	970	-0.854	0.55	0.1554	1.32E-07	6.879	pleckstrin homology domain containing, family N member 1 (Plekhn1)	231002
138	Car2	7.71	603	-0.956	0.52	0.1768	1.76E-07	6.755	carbonic anhydrase 2 (Car2)	12349

139	**Inava**	7.67	803	-0.802	0.57	0.1453	1.37E-07	6.864	innate immunity activator (Inava)	67313
140	**Samd5**	7.64	725	-0.825	0.56	0.1505	1.52E-07	6.817	sterile alpha motif domain containing 5 (Samd5)	320825
141	**Scn5a**	7.62	3826	-0.878	0.54	0.1623	1.81E-07	6.741	sodium channel, voltage-gated, type V, alpha (Scn5a)	20271
142	**Tgm1**	7.61	463	-1.132	0.46	0.2163	3.32E-07	6.479	transglutaminase 1, K polypeptide (Tgm1)	21816
143	**Ahdc1**	7.60	2377	-0.786	0.58	0.1432	1.55E-07	6.810	AT hook, DNA binding motif, containing 1 (Ahdc1)	230793
144	**Klhdc7a**	7.59	347	-1.422	0.37	0.2831	6.82E-07	6.166	kelch domain containing 7A (Klhdc7a)	242721
145	**Muc6**	7.57	367	-1.151	0.45	0.2212	3.83E-07	6.417	mucin 6, gastric (Muc6)	353328
146	**Gm10277**	7.56	167	-1.525	0.35	0.3075	9.23E-07	6.035	predicted gene 10277 (Gm10277)	791303
147	**Zfp579**	7.56	516	-1.083	0.47	0.2067	3.37E-07	6.473	zinc finger protein 579 (Zfp579)	68490
148	**Lmtk3**	7.49	289	-1.106	0.46	0.2124	4.16E-07	6.380	lemur tyrosine kinase 3 (Lmtk3)	381983
149	**Il17re**	7.45	189	-1.542	0.34	0.3157	1.22E-06	5.913	interleukin 17 receptor E (Il17re)	57890
150	**Gm23971**	7.41	1093	-1.025	0.49	0.1964	4.08E-07	6.389	predicted gene, 23971 (Gm23971)	115487918
151	**Wnk4**	7.36	153	-1.417	0.37	0.2875	1.15E-06	5.940	WNK lysine deficient protein kinase 4 (Wnk4)	69847
152	**Kctd14**	7.34	129	-1.961	0.26	0.4321	4.20E-06	5.377	potassium channel tetramerisation domain containing 14 (Kctd14)	233529
153	**Fam83h**	7.31	912	-0.985	0.51	0.1895	4.74E-07	6.325	family with sequence similarity 83, member H (Fam83h)	105732
154	**Prom1**	7.29	1642	-0.899	0.54	0.1712	4.05E-07	6.392	prominin 1 (Prom1)	19126
155	**Mroh3**	7.28	73	-1.886	0.27	0.4094	4.02E-06	5.395	maestro heat-like repeat family member 3 (Mroh3)	76422
156	**Grb7**	7.22	609	-1.045	0.48	0.2047	6.69E-07	6.174	growth factor receptor bound protein 7 (Grb7)	14786
157	**Plch2**	7.21	122	-1.855	0.28	0.4079	4.39E-06	5.358	phospholipase C, eta 2 (Plch2)	269615
158	**Zfp697**	7.20	578	-0.814	0.57	0.1539	4.08E-07	6.389	zinc finger protein 697 (Zfp697)	242109
159	**Adamtsl4**	7.18	1454	-0.899	0.54	0.1726	5.18E-07	6.285	ADAMTS-like 4 (Adamtsl4)	229595
160	**Sh2d4b**	7.17	247	-1.408	0.38	0.2916	1.72E-06	5.765	SH2 domain containing 4B (Sh2d4b)	328381
161	**Vsig10**	7.16	747	-0.872	0.55	0.1673	5.20E-07	6.284	V-set and immunoglobulin domain containing 10 (Vsig10)	231668
162	**Grhl2**	7.14	372	-1.234	0.43	0.2503	1.24E-06	5.908	grainyhead like transcription factor 2 (Grhl2)	252973
163	**Pard6b**	7.13	489	-1.048	0.48	0.2068	8.30E-07	6.081	par-6 family cell polarity regulator beta (Pard6b)	58220
164	**Hook2**	7.12	743	-0.844	0.56	0.1618	5.32E-07	6.274	hook microtubule tethering protein 2 (Hook2)	170833
165	**Tfcp2l1**	7.11	980	-1.272	0.41	0.2611	1.45E-06	5.840	transcription factor CP2-like 1 (Tfcp2l1)	81879
166	**Map3k9**	7.08	367	-1.178	0.44	0.2387	1.26E-06	5.899	mitogen-activated protein kinase kinase kinase 9 (Map3k9)	338372
167	**Erbb3**	7.04	1010	-0.873	0.55	0.1693	6.75E-07	6.171	erb-b2 receptor tyrosine kinase 3 (Erbb3)	13867

168	**Shroom3**	7.03	836	-1.058	0.48	0.2118	1.07E-06	5.970	shroom family member 3 (Shroom3)	27428
169	**Marveld3**	7.01	426	-1.176	0.44	0.2394	1.47E-06	5.831	MARVEL (membrane-associating) domain containing 3 (Marveld3)	73608
170	**4933439C10Rik**	6.93	335	-1.016	0.49	0.2037	1.22E-06	5.913	RIKEN cDNA 4933439C10 gene (4933439C10Rik)	74476
171	**Tgfa**	6.91	814	-0.885	0.54	0.1743	9.53E-07	6.021	transforming growth factor alpha (Tgfa)	21802
172	**Krt87**	6.86	105	-1.631	0.32	0.3610	5.96E-06	5.225	keratin 87 (Krt87)	406219
173	**Sema3f**	6.83	975	-0.879	0.54	0.1744	1.12E-06	5.951	sema domain, immunoglobulin domain (Ig), short basic domain, secreted, (semaphorin) 3F (Sema3f)	20350
174	**Ddi1**	6.83	237	-1.138	0.45	0.2345	2.04E-06	5.690	DNA-damage inducible 1 (Ddi1)	71829
175	**Cdcp1**	6.82	1443	-0.809	0.57	0.1587	9.82E-07	6.008	CUB domain containing protein 1 (Cdcp1)	109332
176	**Kdf1**	6.81	166	-1.358	0.39	0.2898	3.56E-06	5.449	keratinocyte differentiation factor 1 (Kdf1)	69073
177	**Tenm1**	6.80	768	-0.867	0.55	0.1722	1.18E-06	5.928	teneurin transmembrane protein 1 (Tenm1)	23963
178	**Trp63**	6.77	318	-1.198	0.44	0.2517	2.66E-06	5.576	transformation related protein 63 (Trp63)	22061
179	**Mast1**	6.77	256	-1.144	0.45	0.2377	2.38E-06	5.624	microtubule associated serine/threonine kinase 1 (Mast1)	56527
180	**Gm24888**	6.76	70	-1.822	0.28	0.4190	1.16E-05	4.937	predicted gene, 24888 (Gm24888)	115485611
181	**Mylk**	6.76	1264	-0.828	0.56	0.1639	1.18E-06	5.928	myosin, light polypeptide kinase (Mylk)	107589
182	**Map7**	6.72	303	-0.990	0.50	0.2017	1.85E-06	5.734	microtubule-associated protein 7 (Map7)	17761
183	**Lpcat4**	6.67	971	-0.869	0.55	0.1751	1.60E-06	5.797	lysophosphatidylcholine acyltransferase 4 (Lpcat4)	99010
184	**Iqce**	6.63	931	-0.777	0.58	0.1545	1.41E-06	5.850	IQ motif containing E (Iqce)	74239
185	**Dsc2**	6.53	806	-1.075	0.47	0.2274	3.51E-06	5.454	desmocollin 2 (Dsc2)	13506
186	**Ifnlr1**	6.47	187	-1.298	0.41	0.2867	6.74E-06	5.171	interferon lambda receptor 1 (Ifnlr1)	242700
187	**Hoxb5**	6.46	325	-1.346	0.39	0.3012	7.72E-06	5.112	homeobox B5 (Hoxb5)	15413
188	**Foxq1**	6.46	301	-1.261	0.42	0.2777	6.37E-06	5.196	forkhead box Q1 (Foxq1)	15220
189	**Llgl2**	6.46	517	-1.006	0.50	0.2120	3.55E-06	5.449	LLGL2 scribble cell polarity complex component (Llgl2)	217325
190	**Tmem184a**	6.45	232	-1.313	0.40	0.2914	7.31E-06	5.136	transmembrane protein 184a (Tmem184a)	231832
191	**Esrp2**	6.44	409	-1.100	0.47	0.2362	4.57E-06	5.340	epithelial splicing regulatory protein 2 (Esrp2)	77411
192	**Gm17227**	6.44	94	-1.608	0.33	0.3741	1.48E-05	4.831	predicted gene 17227 (Gm17227)	102639888
193	**Cldn2**	6.43	268	-1.225	0.43	0.2687	6.18E-06	5.209	claudin 2 (Cldn2)	12738
194	**D930048N14Rik**	6.43	365	-1.189	0.44	0.2598	5.73E-06	5.242	RIKEN cDNA D930048N14 gene (D930048N14Rik)	97775
195	**Wnt10b**	6.43	284	-1.098	0.47	0.2351	4.68E-06	5.330	wingless-type MMTV integration site family, member 10B (Wnt10b)	22410
196	**Neurl1b**	6.42	669	-0.941	0.52	0.1969	3.33E-06	5.478	neuralized E3 ubiquitin protein ligase 1B (Neurl1b)	240055

197	**Ceacam1**	6.42	682	-0.804	0.57	0.1640	2.44E-06	5.612	CEA cell adhesion molecule 1 (Ceacam1)	26365
198	**Mall**	6.38	167	-1.398	0.38	0.3177	1.03E-05	4.986	mal, T cell differentiation protein-like (Mall)	228576
199	**Prss22**	6.32	820	-0.872	0.55	0.1822	3.55E-06	5.449	serine protease 22 (Prss22)	70835
200	**Lrrc26**	6.31	57	-2.089	0.23	0.5429	5.96E-05	4.225	leucine rich repeat containing 26 (Lrrc26)	227618
201	**Ltbp2**	6.30	868	-0.780	0.58	0.1602	3.02E-06	5.519	latent transforming growth factor beta binding protein 2 (Ltbp2)	16997
202	**Fer1l4**	6.28	45	-2.134	0.23	0.5568	7.11E-05	4.148	fer-1 like family member 4 (Fer1l4)	74562
203	**Prss48**	6.28	145	-1.379	0.38	0.3162	1.26E-05	4.901	serine protease 48 (Prss48)	368202
204	**Nkx2-3**	6.25	274	-1.233	0.43	0.2774	9.68E-06	5.014	NK2 homeobox 3 (Nkx2-3)	18089
205	**Ajuba**	6.22	1641	-0.768	0.59	0.1591	3.51E-06	5.454	ajuba LIM protein (Ajuba)	16475
206	**Ap5z1**	6.20	533	-0.839	0.56	0.1763	4.39E-06	5.357	adaptor-related protein complex 5, zeta 1 subunit (Ap5z1)	231855
207	**Trpv4**	6.19	1213	-0.917	0.53	0.1964	5.34E-06	5.273	transient receptor potential cation channel, subfamily V, member 4 (Trpv4)	63873
208	**Cobl**	6.18	874	-0.924	0.53	0.1982	5.55E-06	5.256	cordon-bleu WH2 repeat (Cobl)	12808
209	**Ppp2r2cos**	6.13	46	-2.010	0.25	0.5239	7.58E-05	4.120	protein phosphatase 2, regulatory subunit B, gamma, opposite strand (Ppp2r2cos)	71054
210	**Nipal2**	6.10	253	-1.001	0.50	0.2192	7.88E-06	5.103	NIPA-like domain containing 2 (Nipal2)	223473
211	**Ucn2**	6.04	115	-1.419	0.37	0.3384	2.38E-05	4.623	urocortin 2 (Ucn2)	171530
212	**Jag2**	5.99	757	-1.016	0.49	0.2271	1.05E-05	4.977	jagged 2 (Jag2)	16450
213	**Sart1**	5.97	1485	-0.754	0.59	0.1602	6.10E-06	5.214	squamous cell carcinoma antigen recognized by T cells 1 (Sart1)	20227
214	**Hunk**	5.96	276	-1.011	0.50	0.2261	1.13E-05	4.946	hormonally upregulated Neu-associated kinase (Hunk)	26559
215	**Plekhg6**	5.96	196	-1.180	0.44	0.2727	1.67E-05	4.777	pleckstrin homology domain containing, family G (with RhoGef domain) member 6 (Plekhg6)	213522
216	**1700001D01Rik**	5.91	90	-1.476	0.36	0.3616	3.69E-05	4.433	RIKEN cDNA 1700001D01 gene (1700001D01Rik)	69279
217	**Pkp3**	5.87	825	-0.846	0.56	0.1855	9.45E-06	5.024	plakophilin 3 (Pkp3)	56460
218	**Unc13a**	5.86	269	-1.007	0.50	0.2275	1.40E-05	4.853	unc-13 homolog A (Unc13a)	382018
219	**Arhgef16**	5.86	166	-1.263	0.42	0.3004	2.56E-05	4.592	Rho guanine nucleotide exchange factor 16 (Arhgef16)	230972
220	**4933407K13Rik**	5.84	477	-0.784	0.58	0.1700	8.90E-06	5.051	RIKEN cDNA 4933407K13 gene (4933407K13Rik)	74396
221	**Ppp1r9a**	5.82	557	-0.823	0.57	0.1804	1.01E-05	4.997	protein phosphatase 1, regulatory subunit 9A (Ppp1r9a)	243725
222	**Gm23472**	5.80	2070	-0.837	0.56	0.1845	1.08E-05	4.965	predicted gene, 23472 (Gm23472)	115487687
223	**Gm35853**	5.80	174	-1.174	0.44	0.2768	2.37E-05	4.625	predicted gene, 35853 (Gm35853)	102639573
224	**Cgn**	5.77	819	-0.964	0.51	0.2192	1.55E-05	4.809	Cingulin (Cgn)	70737

225	**Tincr**	5.76	92	-1.589	0.33	0.4100	6.80E-05	4.168	TINCR ubiquitin domain containing (Tincr)	100504425
226	**Gm50045**	5.75	134	-1.275	0.41	0.3084	3.33E-05	4.478	predicted gene, 50045 (Gm50045)	115488794
227	**Mslnl**	5.67	70	-1.622	0.32	0.4263	8.86E-05	4.052	mesothelin-like (Mslnl)	328783
228	**Krt4**	5.67	136	-1.612	0.33	0.4270	8.72E-05	4.060	keratin 4 (Krt4)	16682
229	**Tnk1**	5.67	218	-1.094	0.47	0.2585	2.66E-05	4.575	tyrosine kinase, non-receptor, 1 (Tnk1)	83813
230	**Ppp1r1b**	5.66	152	-1.587	0.33	0.4182	8.42E-05	4.075	protein phosphatase 1, regulatory inhibitor subunit 1B (Ppp1r1b)	19049
231	**Ccdc120**	5.66	409	-1.046	0.48	0.2458	2.43E-05	4.615	coiled-coil domain containing 120 (Ccdc120)	54648
232	**Adam4**	5.64	182	-1.072	0.48	0.2526	2.69E-05	4.570	a disintegrin and metallopeptidase domain 4 (Adam4)	11498
233	**Krt17**	5.55	7831	-0.885	0.54	0.2041	2.15E-05	4.667	keratin 17 (Krt17)	16667
234	**Pak6**	5.55	221	-1.502	0.35	0.3973	9.05E-05	4.043	p21 (RAC1) activated kinase 6 (Pak6)	214230
235	**Rassf7**	5.52	433	-0.884	0.54	0.2038	2.29E-05	4.639	Ras association (RalGDS/AF-6) domain family (N-terminal) member 7 (Rassf7)	66985
236	**Raver1**	5.49	1187	-0.931	0.52	0.2180	2.78E-05	4.557	ribonucleoprotein, PTB-binding 1 (Raver1)	71766
237	**Atp10b**	5.48	131	-1.237	0.42	0.3094	5.72E-05	4.242	ATPase, class V, type 10B (Atp10b)	319767
238	**Proser2**	5.45	663	-0.852	0.55	0.1972	2.53E-05	4.597	proline and serine rich 2 (Proser2)	227545
239	**Ppp1r13l**	5.42	565	-0.970	0.51	0.2313	3.52E-05	4.453	protein phosphatase 1, regulatory subunit 13 like (Ppp1r13l)	333654
240	**Csf3**	5.42	240	-1.240	0.42	0.3149	6.61E-05	4.180	colony stimulating factor 3 (granulocyte) (Csf3)	12985
241	**Tmem132a**	5.41	1351	-0.770	0.59	0.1754	2.29E-05	4.641	transmembrane protein 132A (Tmem132a)	98170
242	**Phxr4**	5.40	292	-1.008	0.50	0.2433	4.02E-05	4.395	per-hexamer repeat gene 4 (Phxr4)	18689
243	**Hnf1b**	5.38	384	-0.884	0.54	0.2077	3.22E-05	4.492	HNF1 homeobox B (Hnf1b)	21410
244	**Hoxb6**	5.37	562	-1.124	0.46	0.2812	5.63E-05	4.250	homeobox B6 (Hoxb6)	15414
245	**Cdh24**	5.35	280	-1.027	0.49	0.2509	4.73E-05	4.325	cadherin-like 24 (Cdh24)	239096
246	**Trim29**	5.33	122	-1.656	0.32	0.4741	2.10E-04	3.677	tripartite motif-containing 29 (Trim29)	72169
247	**Myo5b**	5.33	873	-0.867	0.55	0.2052	3.48E-05	4.458	myosin VB (Myo5b)	17919
248	**Plekhh1**	5.26	440	-0.815	0.57	0.1918	3.61E-05	4.443	pleckstrin homology domain containing, family H (with MyTH4 domain) member 1 (Plekhh1)	211945
249	**Muc21**	5.25	37	-2.081	0.24	0.6793	6.85E-04	3.164	mucin 21 (Muc21)	672682
250	**Tmc8**	5.24	186	-1.069	0.48	0.2679	6.79E-05	4.168	transmembrane channel-like gene family 8 (Tmc8)	217356
251	**Pex26**	5.23	464	-0.856	0.55	0.2043	4.22E-05	4.375	peroxisomal biogenesis factor 26 (Pex26)	74043
252	**Dennd2d**	5.20	250	-0.899	0.54	0.2175	4.98E-05	4.303	DENN domain containing 2D (Dennd2d)	72121
253	**Igsf9**	5.20	755	-0.831	0.56	0.1983	4.30E-05	4.367	immunoglobulin superfamily, member 9 (Igsf9)	93842

254	**Mapk11**	5.19	471	-0.756	0.59	0.1767	3.67E-05	4.435	mitogen-activated protein kinase 11 (Mapk11)	19094
255	**Camsap3**	5.18	208	-1.153	0.45	0.2987	9.47E-05	4.024	calmodulin regulated spectrin-associated protein family, member 3 (Camsap3)	69697
256	**Slc9a5**	5.16	455	-0.758	0.59	0.1780	3.94E-05	4.405	solute carrier family 9 (sodium/hydrogen exchanger), member 5 (Slc9a5)	277973
257	**Rgl3**	5.13	241	-0.916	0.53	0.2248	6.07E-05	4.217	ral guanine nucleotide dissociation stimulator-like 3 (Rgl3)	71746
258	**Sp6**	5.13	102	-2.381	0.19	0.9023	1.80E-03	2.746	trans-acting transcription factor 6 (Sp6)	83395
259	**Ttll3**	5.05	314	-0.983	0.51	0.2496	8.57E-05	4.067	tubulin tyrosine ligase-like family, member 3 (Ttll3)	101100
260	**Rims2**	5.04	431	-0.827	0.56	0.2015	6.15E-05	4.211	regulating synaptic membrane exocytosis 2 (Rims2)	116838
261	**Mapk10**	5.04	117	-1.324	0.40	0.3675	1.94E-04	3.711	mitogen-activated protein kinase 10 (Mapk10)	26414
262	**Elovl7**	5.02	872	-0.774	0.58	0.1870	5.71E-05	4.244	ELOVL fatty acid elongase 7 (Elovl7)	74559
263	**Aoc2**	5.01	151	-1.112	0.46	0.2933	1.25E-04	3.902	amine oxidase copper containing 2 (Aoc2)	237940
264	**Slurp1**	4.99	56	-1.725	0.30	0.5345	5.41E-04	3.267	secreted Ly6/Plaur domain containing 1 (Slurp1)	57277
265	**Cblc**	4.94	170	-1.247	0.42	0.3452	2.01E-04	3.696	Casitas B-lineage lymphoma c (Cblc)	80794
266	**Gm15222**	4.88	81	-1.321	0.40	0.3762	2.74E-04	3.563	predicted gene 15222 (Gm15222)	102638002
267	**Foxl2os**	4.86	160	-1.113	0.46	0.3014	1.78E-04	3.750	forkhead box L2, opposite strand (Foxl2os)	768252
268	**Col17a1**	4.86	1052	-1.595	0.33	0.5000	5.48E-04	3.261	collagen, type XVII, alpha 1 (Col17a1)	12821
269	**Gm16159**	4.85	169	-1.078	0.47	0.2895	1.70E-04	3.769	predicted gene 16159 (Gm16159)	102633315
270	**Gm25835**	4.81	303	-1.072	0.48	0.2897	1.84E-04	3.734	predicted gene, 25835 (Gm25835)	115487914
271	**Tle2**	4.80	435	-0.837	0.56	0.2132	1.10E-04	3.960	transducin-like enhancer of split 2 (Tle2)	21886
272	**Ripk4**	4.79	397	-0.840	0.56	0.2138	1.12E-04	3.951	receptor-interacting serine-threonine kinase 4 (Ripk4)	72388
273	**Grin1os**	4.78	76	-1.503	0.35	0.4640	5.22E-04	3.282	glutamate receptor, ionotropic, NMDA1 (zeta 1), opposite strand (Grin1os)	320354
274	**Slc26a9**	4.77	163	-1.060	0.48	0.2881	1.97E-04	3.705	solute carrier family 26, member 9 (Slc26a9)	320718
275	**Trpm4**	4.76	816	-0.788	0.58	0.1993	1.08E-04	3.967	transient receptor potential cation channel, subfamily M, member 4 (Trpm4)	68667
276	**Mapk13**	4.73	77	-1.331	0.40	0.3922	3.95E-04	3.403	mitogen-activated protein kinase 13 (Mapk13)	26415
277	**Serpinb10**	4.69	218	-1.003	0.50	0.2723	2.04E-04	3.691	serine (or cysteine) peptidase inhibitor, clade B (ovalbumin), member 10 (Serpinb10)	241197
278	**Epb41l4b**	4.65	350	-0.800	0.57	0.2062	1.42E-04	3.849	erythrocyte membrane protein band 4.1 like 4b (Epb41l4b)	54357
279	**Omg**	4.60	173	-0.985	0.51	0.2698	2.41E-04	3.618	oligodendrocyte myelin glycoprotein (Omg)	18377
280	**Fzd5**	4.60	581	-0.758	0.59	0.1945	1.44E-04	3.842	frizzled class receptor 5 (Fzd5)	14367
281	**Foxj1**	4.59	350	-1.037	0.49	0.2897	2.80E-04	3.553	forkhead box J1 (Foxj1)	15223

282	**Gm11714**	4.57	129	-1.065	0.48	0.3009	3.10E-04	3.509	predicted gene 11714 (Gm11714)	108167930
283	**Gm11767**	4.55	190	-0.936	0.52	0.2557	2.44E-04	3.612	predicted gene 11767 (Gm11767)	102640451
284	**Bcam**	4.50	1013	-0.806	0.57	0.2140	2.01E-04	3.697	basal cell adhesion molecule (Bcam)	57278
285	**Klhdc8b**	4.50	178	-1.050	0.48	0.3006	3.52E-04	3.454	kelch domain containing 8B (Klhdc8b)	78267
286	**Unc13d**	4.49	789	-0.805	0.57	0.2141	2.08E-04	3.682	unc-13 homolog D (Unc13d)	70450
287	**AA986860**	4.48	324	-0.984	0.51	0.2778	3.20E-04	3.494	expressed sequence AA986860 (AA986860)	212439
288	**Zfp612**	4.47	191	-0.935	0.52	0.2596	2.95E-04	3.530	zinc finger protein 612 (Zfp612)	234725
289	**Snhg16**	4.47	365	-0.756	0.59	0.1982	1.95E-04	3.710	small nucleolar RNA host gene 16 (Snhg16)	66293
290	**Plekha7**	4.45	910	-0.844	0.56	0.2294	2.47E-04	3.608	pleckstrin homology domain containing, family A member 7 (Plekha7)	233765
291	**Vtcn1**	4.41	65	-1.434	0.37	0.4773	1.05E-03	2.979	V-set domain containing T cell activation inhibitor 1 (Vtcn1)	242122
292	**Rab11fip1**	4.41	1144	-0.754	0.59	0.2003	2.22E-04	3.654	RAB11 family interacting protein 1 (class I) (Rab11fip1)	75767
293	**Slc25a48**	4.40	430	-0.831	0.56	0.2262	2.68E-04	3.572	solute carrier family 25, member 48 (Slc25a48)	328258
294	**Pcdhga9**	4.40	287	-0.786	0.58	0.2103	2.44E-04	3.613	protocadherin gamma subfamily A, 9 (Pcdhga9)	93717
295	**Card10**	4.36	870	-0.764	0.59	0.2052	2.54E-04	3.596	caspase recruitment domain family, member 10 (Card10)	105844
296	**Slc44a3**	4.34	200	-1.038	0.49	0.3068	4.96E-04	3.305	solute carrier family 44, member 3 (Slc44a3)	213603
297	**Lypd2**	4.28	51	-1.410	0.38	0.4804	1.35E-03	2.869	Ly6/Plaur domain containing 2 (Lypd2)	68311
298	**Nkd2**	4.28	906	-0.805	0.57	0.2231	3.39E-04	3.470	naked cuticle 2 (Nkd2)	72293
299	**Ffar4**	4.27	128	-1.111	0.46	0.3423	6.97E-04	3.157	free fatty acid receptor 4 (Ffar4)	107221
300	**Ifnk**	4.25	29	-1.745	0.30	0.6989	3.13E-03	2.504	interferon kappa (Ifnk)	387510
301	**1700034J05Rik**	4.24	234	-0.812	0.57	0.2260	3.73E-04	3.428	RIKEN cDNA 1700034J05 gene (1700034J05Rik)	73344
302	**1300002E11Rik**	4.23	269	-0.768	0.59	0.2111	3.47E-04	3.460	RIKEN cDNA 1300002E11 gene (1300002E11Rik)	100043489
303	**Uchl4**	4.21	117	-1.088	0.47	0.3347	7.47E-04	3.127	ubiquitin carboxyl-terminal esterase L4 (Uchl4)	93841
304	**Ptpru**	4.18	111	-1.332	0.40	0.4594	1.41E-03	2.850	protein tyrosine phosphatase receptor type U (Ptpru)	19273
305	**Gm4651**	4.18	106	-1.118	0.46	0.3526	8.69E-04	3.061	predicted gene 4651 (Gm4651)	100043793
306	**Oas3**	4.17	415	-1.097	0.47	0.3469	8.43E-04	3.074	2'-5' oligoadenylate synthetase 3 (Oas3)	246727
307	**Gcnt4**	4.16	492	-0.765	0.59	0.2127	4.00E-04	3.398	glucosaminyl (N-acetyl) transferase 4, core 2 (beta-1,6-N-acetylglucosaminyltransferase) (Gcnt4)	218476
308	**Krtcap3**	4.15	277	-0.866	0.55	0.2510	5.24E-04	3.281	keratinocyte associated protein 3 (Krtcap3)	69815
309	**Hoxb5os**	4.15	97	-1.451	0.37	0.5377	2.02E-03	2.695	homeobox B5 and homeobox B6, opposite strand (Hoxb5os)	75395
310	**Ap1m2**	4.14	142	-0.975	0.51	0.2940	6.85E-04	3.164	adaptor protein complex AP-1, mu 2 subunit (Ap1m2)	11768

311	**9330151L19Rik**	4.14	187	-0.884	0.54	0.2583	5.60E-04	3.252	RIKEN cDNA 9330151L19 gene (9330151L19Rik)	414085
312	**9130230L23Rik**	4.12	57	-1.323	0.40	0.4575	1.61E-03	2.795	RIKEN cDNA 9130230L23 gene (9130230L23Rik)	231253
313	**Gm5148**	4.11	170	-0.935	0.52	0.2801	6.62E-04	3.179	predicted gene 5148 (Gm5148)	381438
314	**Cfap47**	4.11	39	-1.988	0.25	1.0205	7.63E-03	2.117	cilia and flagella associated protein 47 (Cfap47)	636104
315	**Psg17**	4.10	61	-1.294	0.41	0.4439	1.55E-03	2.810	pregnancy specific beta-1-glycoprotein 17 (Psg17)	26437
316	**Crb3**	4.10	126	-1.048	0.48	0.3278	8.89E-04	3.051	crumbs family member 3 (Crb3)	224912
317	**Dnhd1**	4.09	163	-0.932	0.52	0.2805	6.93E-04	3.159	dynein heavy chain domain 1 (Dnhd1)	77505
318	**Barx2**	4.08	323	-0.858	0.55	0.2523	6.03E-04	3.219	BarH-like homeobox 2 (Barx2)	12023
319	**Slc5a9**	4.04	47	-1.594	0.33	0.6580	3.59E-03	2.445	solute carrier family 5 (sodium/glucose cotransporter), member 9 (Slc5a9)	230612
320	**Gm12592**	3.99	121	-1.015	0.49	0.3224	1.06E-03	2.973	predicted gene 12592 (Gm12592)	791413
321	**Cldn7**	3.99	924	-0.766	0.59	0.2217	6.02E-04	3.221	claudin 7 (Cldn7)	53624
322	**Ckmt1**	3.96	98	-1.155	0.45	0.3926	1.57E-03	2.804	creatine kinase, mitochondrial 1, ubiquitous (Ckmt1)	12716
323	**Omp**	3.93	60	-1.379	0.38	0.5276	2.81E-03	2.551	olfactory marker protein (Omp)	18378
324	**Arfgef3**	3.89	219	-0.851	0.55	0.2600	9.25E-04	3.034	ARFGEF family member 3 (Arfgef3)	215821
325	**Tjp3**	3.87	106	-1.101	0.47	0.3765	1.72E-03	2.764	tight junction protein 3 (Tjp3)	27375
326	**Hid1**	3.84	335	-0.872	0.55	0.2730	1.07E-03	2.971	HID1 domain containing (Hid1)	217310
327	**Gm19221**	3.81	211	-0.787	0.58	0.2378	9.43E-04	3.025	predicted gene, 19221 (Gm19221)	100418453
328	**G630016G05Rik**	3.79	119	-0.952	0.52	0.3110	1.44E-03	2.841	RIKEN cDNA G630016G05 gene (G630016G05Rik)	108168785
329	**Bspry**	3.75	250	-0.787	0.58	0.2416	1.09E-03	2.964	B-box and SPRY domain containing (Bspry)	192120
330	**Prkcz**	3.74	270	-0.840	0.56	0.2655	1.25E-03	2.904	protein kinase C, zeta (Prkcz)	18762
331	**Gm2710**	3.71	88	-1.076	0.47	0.3827	2.33E-03	2.632	predicted gene 2710 (Gm2710)	108167606
332	**Mlph**	3.68	232	-0.804	0.57	0.2540	1.34E-03	2.874	Melanophilin (Mlph)	171531
333	**Hecw1**	3.66	51	-1.337	0.40	0.5597	4.72E-03	2.326	HECT, C2 and WW domain containing E3 ubiquitin protein ligase 1 (Hecw1)	94253
334	**Sowahb**	3.64	116	-1.006	0.50	0.3525	2.30E-03	2.639	sosondowah ankyrin repeat domain family member B (Sowahb)	78088
335	**Sorbs2os**	3.64	227	-0.942	0.52	0.3214	1.99E-03	2.700	sorbin and SH3 domain containing 2, opposite strand (Sorbs2os)	319940
336	**Vmn1r43**	3.63	121	-0.918	0.53	0.3090	1.95E-03	2.710	vomeronasal 1 receptor 43 (Vmn1r43)	113847
337	**Large2**	3.63	190	-0.939	0.52	0.3214	2.06E-03	2.687	LARGE xylosyl- and glucuronyltransferase 2 (Large2)	228366
338	**Artn**	3.62	214	-0.966	0.51	0.3368	2.21E-03	2.656	Artemin (Artn)	11876

339	**Rab17**	3.60	23	-1.568	0.34	0.7918	9.21E-03	2.036	RAB17, member RAS oncogene family (Rab17)	19329
340	**Dqx1**	3.58	88	-1.144	0.45	0.4422	3.68E-03	2.435	DEAQ RNA-dependent ATPase (Dqx1)	93838
341	**9430037G07Rik**	3.57	208	-0.783	0.58	0.2518	1.63E-03	2.787	RIKEN cDNA 9430037G07 gene(9430037G07Rik)	320692
342	**Kcnh7**	3.55	167	-0.870	0.55	0.2933	2.09E-03	2.679	potassium voltage-gated channel, subfamily H (eag-related), member 7(Kcnh7)	170738
343	**Gm13648**	3.54	62	-1.181	0.44	0.4701	4.33E-03	2.364	predicted gene 13648 (Gm13648)	100415901
344	**AU040972**	3.54	112	-0.949	0.52	0.3340	2.58E-03	2.589	expressed sequence AU040972 (AU040972)	104522
345	**Rn7s1**	3.53	#####	-1.272	0.41	0.5516	5.55E-03	2.256	7S RNA 1 (Rn7s1)	103948
346	**Dsg3**	3.51	503	-0.781	0.58	0.2556	1.86E-03	2.730	desmoglein 3 (Dsg3)	13512
347	**Crnde**	3.50	178	-0.783	0.58	0.2554	1.90E-03	2.722	colorectal neoplasia differentially expressed, non-protein coding (Crnde)	71296
348	**Gm7162**	3.50	88	-1.053	0.48	0.3949	3.56E-03	2.448	predicted gene 7162 (Gm7162)	635617
349	**Tmie**	3.50	108	-0.946	0.52	0.3357	2.80E-03	2.553	transmembrane inner ear (Tmie)	20776
350	**Gm18942**	3.49	69	-1.098	0.47	0.4257	4.08E-03	2.390	predicted gene, 18942 (Gm18942)	100418004
351	**Lemd1**	3.47	162	-0.824	0.56	0.2780	2.28E-03	2.642	LEM domain containing 1 (Lemd1)	213409
352	**Snora57**	3.45	149	-0.841	0.56	0.2872	2.45E-03	2.611	small nucleolar RNA, H/ACA box 57 (Snora57)	118027464
353	**2700054A10Rik**	3.45	134	-0.912	0.53	0.3241	2.90E-03	2.537	RIKEN cDNA 2700054A10 gene (2700054A10Rik)	72578
354	**1700016C15Rik**	3.44	76	-1.114	0.46	0.4444	4.70E-03	2.328	#N/A	#N/A
355	**Echdc2**	3.42	286	-0.780	0.58	0.2608	2.27E-03	2.644	enoyl Coenzyme A hydratase domain containing 2 (Echdc2)	52430
356	**Grhl1**	3.42	104	-0.927	0.53	0.3350	3.21E-03	2.493	grainyhead like transcription factor 1 (Grhl1)	195733
357	**Acrbp**	3.42	106	-0.917	0.53	0.3298	3.16E-03	2.501	proacrosin binding protein (Acrbp)	54137
358	**4930592I03Rik**	3.39	104	-0.952	0.52	0.3527	3.67E-03	2.435	RIKEN cDNA 4930592I03 gene (4930592I03Rik)	75853
359	**Kazn**	3.38	288	-0.807	0.57	0.2782	2.70E-03	2.569	kazrin, periplakin interacting protein (Kazn)	71529
360	**Tchh**	3.36	102	-0.927	0.53	0.3416	3.67E-03	2.435	Trichohyalin (Tchh)	99681
361	**Col9a3**	3.34	45	-1.250	0.42	0.5840	8.10E-03	2.091	collagen, type IX, alpha 3 (Col9a3)	12841
362	**Pclo**	3.34	166	-0.836	0.56	0.2950	3.15E-03	2.501	piccolo (presynaptic cytomatrix protein) (Pclo)	26875
363	**AI463170**	3.31	71	-1.019	0.49	0.4060	5.16E-03	2.287	expressed sequence AI463170 (AI463170)	100504549
364	**Trpv3**	3.30	94	-0.977	0.51	0.3791	4.70E-03	2.328	transient receptor potential cation channel, subfamily V, member 3 (Trpv3)	246788
365	**A230103J11Rik**	3.30	132	-0.829	0.56	0.2947	3.39E-03	2.470	RIKEN cDNA A230103J11 gene (A230103J11Rik)	320466
366	**4930412M03Rik**	3.30	152	-0.911	0.53	0.3423	4.11E-03	2.386	RIKEN cDNA 4930412M03 gene (4930412M03Rik)	100504140
367	**Gm16617**	3.30	97	-0.923	0.53	0.3475	4.24E-03	2.373	predicted gene, 16617 (Gm16617)	100502764

368	Rptoros	3.29	61	-1.093	0.47	0.4614	6.33E-03	2.199	regulatory associated protein of MTOR, complex 1, opposite strand (Rptoros)	319454
369	Hoxb4	3.28	400	-0.817	0.57	0.2925	3.41E-03	2.468	homeobox B4 (Hoxb4)	15412
370	Rab11fip4	3.28	181	-0.839	0.56	0.3031	3.60E-03	2.444	RAB11 family interacting protein 4 (class II) (Rab11fip4)	268451
371	Adam1a	3.27	120	-0.867	0.55	0.3191	3.97E-03	2.401	a disintegrin and metallopeptidase domain 1a (Adam1a)	280668
372	Kirrel3	3.26	141	-0.816	0.57	0.2928	3.63E-03	2.441	kirre like nephrin family adhesion molecule 3 (Kirrel3)	67703
373	Pof1b	3.25	201	-0.755	0.59	0.2618	3.21E-03	2.494	premature ovarian failure 1B (Pof1b)	69693
374	Map3k12	3.22	908	-0.773	0.59	0.2759	3.60E-03	2.444	mitogen-activated protein kinase kinase kinase 12 (Map3k12)	26404
375	Pitpnm2os2	3.18	55	-1.070	0.48	0.4701	7.77E-03	2.110	phosphatidylinositol transfer protein, membrane-associated 2, opposite strand 2 (Pitpnm2os2)	100047482
376	Clca1	3.18	129	-0.876	0.54	0.3364	4.98E-03	2.303	chloride channel accessory 1 (Clca1)	23844
377	Gm15708	3.18	168	-0.852	0.55	0.3228	4.71E-03	2.327	predicted gene 15708 (Gm15708)	100504231
378	2310002F09Rik	3.14	103	-0.873	0.55	0.3382	5.39E-03	2.268	RIKEN cDNA 2310002F09 gene (2310002F09Rik)	100504720
379	Rgs11	3.14	110	-0.929	0.53	0.3761	6.14E-03	2.212	regulator of G-protein signaling 11 (Rgs11)	50782
380	Gm35612	3.14	57	-1.048	0.48	0.4614	8.09E-03	2.092	predicted gene, 35612 (Gm35612)	102639259
381	Kif27	3.12	104	-0.978	0.51	0.4150	7.13E-03	2.147	kinesin family member 27 (Kif27)	75050
382	Gm3764	3.11	42	-1.201	0.44	0.6311	1.24E-02	1.905	predicted gene 3764 (Gm3764)	100042277
383	Trpv6	3.06	59	-1.165	0.45	0.6160	1.27E-02	1.897	transient receptor potential cation channel, subfamily V, member 6 (Trpv6)	64177
384	Gjb2	3.06	199	-0.875	0.55	0.3545	6.57E-03	2.182	gap junction protein, beta 2 (Gjb2)	14619
385	Gm49221	3.06	31	-1.238	0.42	0.7140	1.52E-02	1.819	predicted gene, 49221 (Gm49221)	115488806
386	Slc5a5	3.04	61	-1.104	0.47	0.5542	1.15E-02	1.939	solute carrier family 5 (sodium iodide symporter), member 5 (Slc5a5)	114479
387	Gpa33	3.04	163	-0.806	0.57	0.3111	5.80E-03	2.236	glycoprotein A33 transmembrane (Gpa33)	59290
388	Slc37a1	3.04	186	-0.802	0.57	0.3096	5.76E-03	2.240	solute carrier family 37 (glycerol-3-phosphate transporter), member 1 (Slc37a1)	224674
389	Lypd6b	3.03	26	-1.295	0.41	0.8480	1.83E-02	1.739	LY6/PLAUR domain containing 6B (Lypd6b)	71897
390	Gm12216	3.03	99	-0.856	0.55	0.3431	6.66E-03	2.176	predicted gene 12216 (Gm12216)	622459
391	Btn1a1	3.03	137	-0.797	0.58	0.3072	5.87E-03	2.231	butyrophilin, subfamily 1, member A1 (Btn1a1)	12231
392	Fut9	3.01	221	-0.892	0.54	0.3756	7.67E-03	2.115	fucosyltransferase 9 (Fut9)	14348
393	Grin1	3.00	35	-1.156	0.45	0.6223	1.42E-02	1.849	glutamate receptor, ionotropic, NMDA1 (zeta 1) (Grin1)	14810
394	Snord3b3	3.00	4154	-0.926	0.53	0.3993	8.39E-03	2.076	small nucleolar RNA, C/D box 3B3 (Snord3b3)	19860
395	Ksr2	2.99	64	-0.970	0.51	0.4369	9.66E-03	2.015	kinase suppressor of ras 2 (Ksr2)	333050

396	**Camk1g**	2.98	64	-0.974	0.51	0.4412	9.97E-03	2.001	calcium/calmodulin-dependent protein kinase I gamma (Camk1g)	215303
397	**Gm19461**	2.97	38	-1.159	0.45	0.6510	1.55E-02	1.809	predicted gene, 19461 (Gm19461)	100502933
398	**Bcas3os1**	2.96	131	-0.826	0.56	0.3335	7.27E-03	2.138	BCAS3 microtubule associated cell migration factor, opposite strand 1 (Bcas3os1)	71489
399	**Mab21l2**	2.96	49	-1.054	0.48	0.5259	1.24E-02	1.906	mab-21-like 2 (Mab21l2)	23937
400	**Clic3**	2.95	87	-0.886	0.54	0.3766	8.55E-03	2.068	chloride intracellular channel 3 (Clic3)	69454
401	**Slc44a4**	2.95	61	-0.971	0.51	0.4451	1.04E-02	1.982	solute carrier family 44, member 4 (Slc44a4)	70129
402	**Lmntd2**	2.95	82	-0.937	0.52	0.4194	9.78E-03	2.009	lamin tail domain containing 2 (Lmntd2)	72000
403	**Gm24497**	2.95	1431	-1.109	0.46	0.6056	1.46E-02	1.836	predicted gene, 24497 (Gm24497)	115487688
404	**Srd5a2**	2.93	49	-1.095	0.47	0.5931	1.47E-02	1.832	steroid 5 alpha-reductase 2 (Srd5a2)	94224
405	**Islr2**	2.92	217	-0.764	0.59	0.3013	7.03E-03	2.153	immunoglobulin superfamily containing leucine-rich repeat 2 (Islr2)	320563
406	**Iqck**	2.90	145	-0.824	0.56	0.3433	8.43E-03	2.074	IQ motif containing K (Iqck)	434232
407	**Prkcg**	2.90	77	-0.904	0.53	0.4030	1.01E-02	1.994	protein kinase C, gamma (Prkcg)	18752
408	**Gm35715**	2.89	72	-0.903	0.53	0.4045	1.04E-02	1.984	predicted gene, 35715 (Gm35715)	102639389
409	**Gm18398**	2.89	26	-1.203	0.43	0.8030	2.07E-02	1.684	predicted gene, 18398 (Gm18398)	100417103
410	**Fam227a**	2.88	97	-0.879	0.54	0.3869	1.00E-02	1.999	family with sequence similarity 227, member A (Fam227a)	75729
411	**AU022754**	2.87	81	-0.921	0.53	0.4259	1.11E-02	1.953	expressed sequence AU022754 (AU022754)	105975
412	**Nron**	2.86	70	-0.912	0.53	0.4186	1.12E-02	1.950	non-protein coding RNA, repressor of NFAT (Nron)	320482
413	**Wdr46-ps**	2.86	104	-0.852	0.55	0.3691	9.79E-03	2.009	Wdr46 retrotransposed pseudogene (Wdr46-ps)	100416160
414	**Pla2g4b**	2.85	66	-1.055	0.48	0.5860	1.60E-02	1.796	phospholipase A2, group IVB (cytosolic) (Pla2g4b)	211429
415	**Niban3**	2.84	66	-1.043	0.49	0.5768	1.58E-02	1.802	niban apoptosis regulator 3 (Niban3)	100037278
416	**Snord3b1**	2.82	4465	-0.914	0.53	0.4354	1.25E-02	1.903	small nucleolar RNA, C/D box 3B1 (Snord3b1)	19858
417	**Slc2a4rg-ps**	2.81	139	-0.768	0.59	0.3180	9.03E-03	2.044	Slc2a4 regulator, pseudogene (Slc2a4rg-ps)	329584
418	**Bc1**	2.80	198	-0.823	0.57	0.3577	1.05E-02	1.979	brain cytoplasmic RNA 1 (Bc1)	100568459
419	**Nectin4**	2.80	162	-0.777	0.58	0.3273	9.58E-03	2.019	nectin cell adhesion molecule 4 (Nectin4)	71740
420	**Doc2g**	2.79	66	-0.919	0.53	0.4469	1.36E-02	1.867	double C2, gamma (Doc2g)	60425
421	**Gcnt3**	2.77	110	-0.826	0.56	0.3689	1.15E-02	1.940	glucosaminyl (N-acetyl) transferase 3, mucin type (Gcnt3)	72077
422	**Gm7241**	2.74	82	-0.857	0.55	0.4000	1.29E-02	1.888	predicted pseudogene 7241 (Gm7241)	638532
423	**Phldb3**	2.74	287	-0.794	0.58	0.3525	1.14E-02	1.944	pleckstrin homology like domain, family B, member 3 (Phldb3)	232970

424	**Cdk5r1**	2.73	121	-0.764	0.59	0.3273	1.07E-02	1.970	cyclin dependent kinase 5, regulatory subunit 1 (Cdk5r1)	12569
425	**Lenep**	2.72	44	-0.980	0.51	0.5413	1.83E-02	1.737	lens epithelial protein (Lenep)	57275
426	**B230206H07Rik**	2.70	96	-0.784	0.58	0.3477	1.22E-02	1.915	RIKEN cDNA B230206H07 gene (B230206H07Rik)	320871
427	**1810019D21Rik**	2.69	107	-0.921	0.53	0.4934	1.71E-02	1.766	RIKEN cDNA 1810019D21 gene (1810019D21Rik)	69771
428	**Exph5**	2.68	123	-0.753	0.59	0.3278	1.18E-02	1.929	exophilin 5 (Exph5)	320051
429	**Ly6g**	2.67	38	-1.043	0.49	0.6734	2.36E-02	1.627	lymphocyte antigen 6 family member G (Ly6g)	546644
430	**Ttc6**	2.65	46	-0.952	0.52	0.5419	2.02E-02	1.695	tetratricopeptide repeat domain 6 (Ttc6)	70846
431	**Tas1r3**	2.64	112	-0.847	0.56	0.4246	1.62E-02	1.789	taste receptor, type 1, member 3 (Tas1r3)	83771
432	**F830208F22Rik**	2.63	56	-0.904	0.53	0.4880	1.90E-02	1.722	RIKEN cDNA F830208F22 gene (F830208F22Rik)	619308
433	**Muc5b**	2.62	47	-0.968	0.51	0.5853	2.24E-02	1.650	mucin 5, subtype B, tracheobronchial (Muc5b)	74180
434	**Slc12a5**	2.61	76	-0.826	0.56	0.4071	1.66E-02	1.780	solute carrier family 12, member 5 (Slc12a5)	57138
435	**Klc3**	2.58	213	-0.786	0.58	0.3804	1.60E-02	1.795	kinesin light chain 3 (Klc3)	232943
436	**Xkr6**	2.58	99	-0.795	0.58	0.3864	1.65E-02	1.783	X-linked Kx blood group related 6 (Xkr6)	219149
437	**Mm2pr**	2.56	58	-0.928	0.53	0.5599	2.34E-02	1.630	macrophage M2 polarization regulator (Mm2pr)	433966
438	**Sprr2a2**	2.55	150	-0.798	0.58	0.4002	1.77E-02	1.753	small proline-rich protein 2A2 (Sprr2a2)	100303744
439	**Parvaos**	2.55	45	-0.925	0.53	0.5522	2.38E-02	1.623	parvin, alpha, opposite strand (Parvaos)	102636183
440	**Tcte2**	2.54	77	-0.803	0.57	0.4019	1.81E-02	1.741	t-complex-associated testis expressed 2 (Tcte2)	21646
441	**Otop1**	2.54	186	-0.787	0.58	0.3930	1.78E-02	1.750	otopetrin 1 (Otop1)	21906
442	**Nscme3l**	2.54	42	-0.951	0.52	0.6108	2.60E-02	1.584	NSE3 homolog, SMC5-SMC6 complex component like (Nscme3l)	75555
443	**Nradd**	2.51	106	-0.791	0.58	0.4033	1.91E-02	1.720	neurotrophin receptor associated death domain (Nradd)	67169
444	**Aifm3**	2.51	44	-0.922	0.53	0.5785	2.58E-02	1.588	apoptosis-inducing factor, mitochondrion-associated 3 (Aifm3)	72168
445	**Gm43915**	2.50	105	-0.787	0.58	0.4002	1.92E-02	1.718	predicted gene, 43915 (Gm43915)	215458
446	**1700007J10Rik**	2.50	105	-0.751	0.59	0.3618	1.78E-02	1.750	RIKEN cDNA 1700007J10 gene (1700007J10Rik)	69320
447	**Sprr2a1**	2.49	131	-0.781	0.58	0.4005	1.97E-02	1.706	small proline-rich protein 2A1 (Sprr2a1)	20755
448	**3830408C21Rik**	2.43	78	-0.809	0.57	0.4493	2.38E-02	1.624	RIKEN cDNA 3830408C21 gene (3830408C21Rik)	100040322
449	**Serpinc1**	2.42	85	-0.780	0.58	0.4168	2.27E-02	1.644	serine (or cysteine) peptidase inhibitor, clade C (antithrombin), member 1 (Serpinc1)	11905
450	**Gm23444**	2.41	682	-0.909	0.53	0.6559	3.14E-02	1.504	predicted gene, 23444 (Gm23444)	115487915
451	**Gpr52**	2.40	62	-0.797	0.58	0.4423	2.48E-02	1.605	G protein-coupled receptor 52 (Gpr52)	620246
452	**Arhgap40**	2.40	55	-0.882	0.54	0.6054	3.03E-02	1.519	Rho GTPase activating protein 40 (Arhgap40)	545481

453	**Krt42**	2.40	72	-0.794	0.58	0.4443	2.51E-02	1.601	keratin 42 (Krt42)	68239
454	**Gm25203**	2.39	29	-0.943	0.52	0.7930	3.55E-02	1.450	predicted gene, 25203 (Gm25203)	115488090
455	**Iqank1**	2.39	48	-0.857	0.55	0.5499	2.94E-02	1.532	IQ motif and ankyrin repeat containing 1 (Iqank1)	432964
456	**Mir5125**	2.38	44	-0.872	0.55	0.5799	3.08E-02	1.512	microRNA 5125 (Mir5125)	100628593
457	**Gm17907**	2.38	67	-0.779	0.58	0.4256	2.49E-02	1.604	predicted gene, 17907 (Gm17907)	100416076
458	**1500015L24Rik**	2.38	64	-0.790	0.58	0.4418	2.56E-02	1.591	RIKEN cDNA 1500015L24 gene (1500015L24Rik)	68994
459	**Fam187b**	2.34	58	-0.867	0.55	0.6418	3.36E-02	1.473	family with sequence similarity 187, member B (Fam187b)	76415
460	**Syne4**	2.33	67	-0.820	0.57	0.5247	3.09E-02	1.510	spectrin repeat containing, nuclear envelope family member 4 (Syne4)	233066
461	**Muc2**	2.30	42	-0.851	0.55	0.6166	3.53E-02	1.453	mucin 2 (Muc2)	17831
462	**Tnk2os**	2.30	61	-0.793	0.58	0.4877	3.08E-02	1.511	tyrosine kinase, non-receptor 2, opposite strand (Tnk2os)	675921
463	**Slc5a8**	2.30	33	-0.879	0.54	0.7115	3.81E-02	1.419	solute carrier family 5 (iodide transporter), member 8 (Slc5a8)	216225
464	**Ildr1**	2.28	81	-0.790	0.58	0.5098	3.26E-02	1.486	immunoglobulin-like domain containing receptor 1 (Ildr1)	106347
465	**Wfikkn1**	2.25	48	-0.807	0.57	0.5574	3.60E-02	1.443	WAP, FS, Ig, KU, and NTR-containing protein 1 (Wfikkn1)	215001
466	**Cxcl17**	2.24	68	-0.775	0.58	0.4977	3.39E-02	1.470	C-X-C motif chemokine ligand 17 (Cxcl17)	232983
467	**Gm41609**	2.23	77	-0.780	0.58	0.5245	3.57E-02	1.448	predicted gene, 41609 (Gm41609)	105246306
468	**Bicdl2**	2.20	70	-0.752	0.59	0.4812	3.54E-02	1.451	BICD family like cargo adaptor 2 (Bicdl2)	212733
469	**Gm11557**	2.20	47	-0.787	0.58	0.5572	3.88E-02	1.412	predicted gene 11557 (Gm11557)	665922
470	**Cbs**	2.19	42	-0.815	0.57	0.7095	4.22E-02	1.375	cystathionine beta-synthase (Cbs)	12411
471	**Cntd1**	2.16	45	-0.772	0.59	0.5562	4.11E-02	1.386	cyclin N-terminal domain containing 1 (Cntd1)	68107
472	**Psca**	2.14	31	-0.802	0.57	0.7177	4.59E-02	1.338	prostate stem cell antigen (Psca)	72373
473	**Gm18829**	2.13	39	-0.780	0.58	0.6230	4.47E-02	1.350	predicted gene, 18829 (Gm18829)	100417792
474	**4732490B19Rik**	2.11	32	-0.784	0.58	0.7071	4.77E-02	1.322	RIKEN cDNA 4732490B19 gene (4732490B19Rik)	319871
475	**2310043M15Rik**	2.10	34	-0.783	0.58	0.8282	4.76E-02	1.322	RIKEN cDNA 2310043M15 gene (2310043M15Rik)	102633000
476	**Snora78**	2.10	48	-0.753	0.59	0.5856	4.52E-02	1.345	small nucleolar RNA, H/ACA box 7 (Snora78)	100306952
